# Protoporphyrin IX-Derived Ruthenium(II) Complexes for Photodynamic Therapy in Gastric Cancer Cells

**DOI:** 10.1021/acs.inorgchem.5c00896

**Published:** 2025-05-02

**Authors:** Andrés Restrepo-Acevedo, María Isabel Murillo, Christophe Orvain, Chloé Thibaudeau, Sevda Recberlik, Lucas Verget, Virginia Gómez Vidales, Christian Gaiddon, Georg Mellitzer, Ronan Le Lagadec

**Affiliations:** † Universidad Nacional Autonoma de México, Instituto de Química UNAM, Circuito Exterior s/n Ciudad Universitaria, 04510 Ciudad de México, Mexico; ‡ Inserm UMR_S U1113; IRFAC, 3 Avenue Molière, 67200 Strasbourg, France; § Faculté de Chimie, Sorbonne Université, 4 place Jussieu, 75005 Paris, France

## Abstract

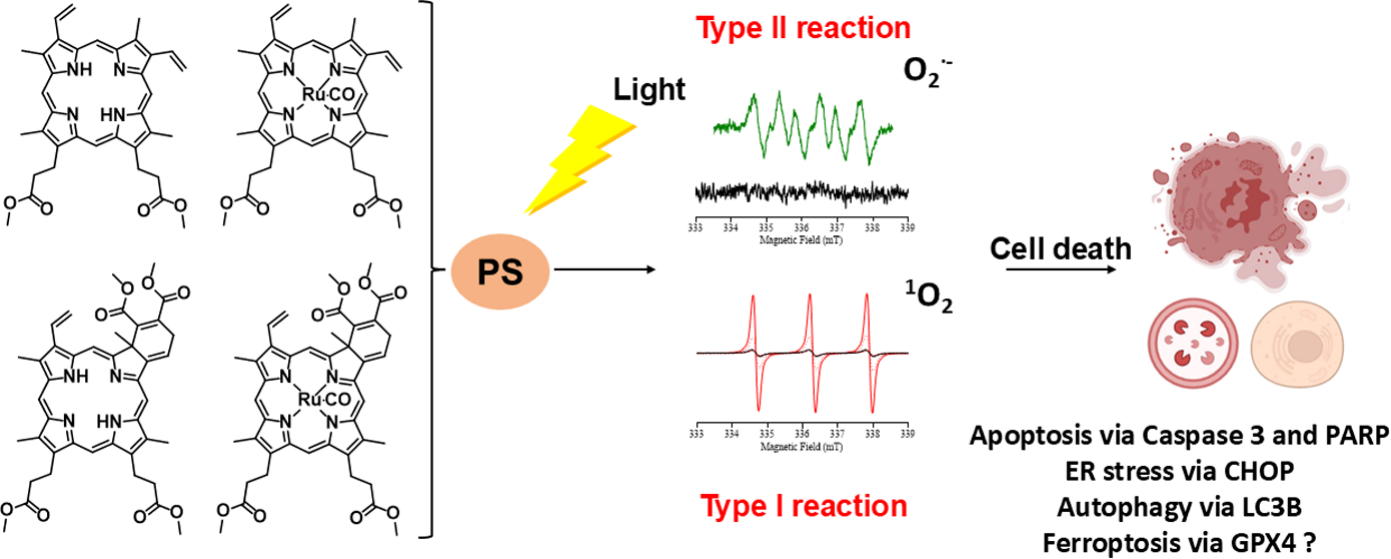

In recent years, photodynamic therapy (PDT) has emerged as a promising alternative to classical chemotherapy for treating cancer. PDT is based on a nontoxic prodrug called photosensitizer (PS) activated by light at the desired location. Upon irradiation, the PS reacts with the oxygen present in the tumor, producing cytotoxic reactive oxygen species (ROS). Compounds with highly conjugated π-bond systems, such as porphyrins and chlorins, have proven to be excellent light scavengers, and introducing a metal atom in their structure improved the generation of ROS. In this work, a series of tetrapyrrole-ruthenium(II) complexes derived from protoporphyrin IX and the commercial drug verteporfin were designed as photosensitizers for PDT. The complexes were almost nontoxic on human gastric cancer cells under dark conditions, revealing remarkable cytotoxicity upon irradiation with light. The ruthenium atom in the central cavity of the chlorin ligand allowed combined mechanisms in photodynamic therapy, as both singlet oxygen and superoxide radicals were detected. Additionally, one complex produced large amounts of singlet oxygen under hypoxic conditions. Biological assays demonstrated that the ruthenium derivatives caused cell death through a caspase 3 mediated apoptotic pathway and *via* CHOP, an endoplasmic reticulum stress-inducible transcription factor involved in apoptosis and growth arrest.

## Abstract 2

Tetrapyrrole-ruthenium(II) compounds were designed as photosensitizers for PDT. The complexes exhibited low toxicity in the dark on human gastric cancer cells and remarkable cytotoxicity upon irradiation with light. Both singlet oxygen and superoxide radicals can be produced, even at low oxygen concentrations. Cell death is induced *via* a caspase 3 mediated apoptotic pathway and *via* CHOP, an endoplasmic reticulum stress transcription factor, potentially making the complexes less vulnerable to drug-resistance mechanisms.

## Introduction

Photodynamic therapy (PDT) is an approved anticancer strategy with high temporal selectivity, which presents several advantages over conventional radiotherapy and chemotherapy, such as few side effects and as a noninvasive therapy.^1,2^ Platinum-based complexes, such as cisplatin, carboplatin, and oxaliplatin, have become the most used metal-based drugs worldwide for cancer treatment by chemotherapy.^3−5^ However, although these anticancer drugs are very effective, their efficiency is limited due to the high incidence of resistance mechanisms. In addition, they lack selectivity toward cancer cells, which leads to numerous undesirable side effects such as nephrotoxicity, neurotoxicity, and ototoxicity, among others.^6,7^ As an alternative to classical chemotherapy, PDT emerged as a promising strategy for treating cancer. Treatments by PDT are based on a nontoxic prodrug called a photosensitizer (PS) that can be activated by light at the desired location.^8,9^ Upon photoactivation, the PS releases the energy needed to react with the oxygen present in the tumor, producing reactive oxygen species (ROS) such as singlet oxygen (^1^O_2_), superoxide radical (O_2_^•**–**^), hydroxyl radical (^•^OH) and hydrogen peroxide (H_2_O_2_). Such species are spatially and temporally confined to the irradiated region, thus targeting malignant tissues without attacking healthy ones.^10^ Two types of PDT have been described. In type II reaction, high amounts of highly toxic singlet oxygen are produced, causing oxidative damage in cells. In type I reaction, the PS in singlet or triplet excited state can react directly with a biological substrate and undergo hydrogen atom abstraction or electron transfer reactions, giving rise to highly reactive free radicals and radical ions, which can generate different ROS and cause cell death.^11−13^

**Figure 1:**
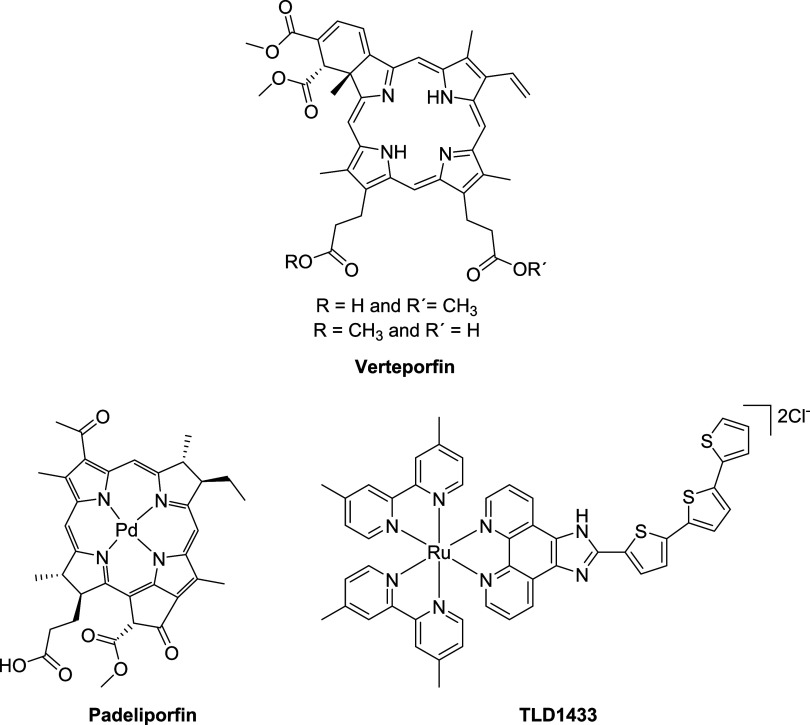
Compounds approved or in clinical trials as photosensitizers for PDT.

Ideally, a PS should preferentially (i) accumulate in tumors, (ii) display no toxicity in the dark, and (iii) be rapidly eliminated from the body.^14,15^ The PS should also present a high quantum yield of ROS production, a high molar extinction coefficient, and molar absorbance. Absorbance in the red spectral regions (600–800 nm) is desired for deep-seated tumors, *i.e.*, in cancers of the stomach, bladder, pancreas, esophageal or glioblastoma multiforme, among others.^14,16^ Compounds with highly conjugated π-bond systems, such as porphyrins and chlorins, are excellent light scavengers and are, therefore, ideal PS for PDT.^17^ Additionally, such tetrapyrrole structures can host a wide variety of metal ions. For example, it has been observed that metaloporphyrins with paramagnetic metal ions such as Cu(II) or Co(II) present low ROS production due to a decrease in the lifetime of the excited triplet state of the PS. On the contrary, metaloporphyrins with diamagnetic ions such as Zn(II), Pd(II), In(III), Ru(II), Ir(III), and Pt(II) present the heavy atom effect with a strong spin–orbital coupling that provides an absorbance lifetime long enough for the excited triplet state to interact with dioxygen, thus favoring the generation of ROS and, therefore, increasing the quantum yields.^13,18,19^ For instance, a palladium(II) metalochlorin complex (padeliporfin, TOOKAD) has been approved in several EU countries, Israel and Mexico, for use as a PS for the treatment of localized prostate cancer (Figure 1).^20^ Ruthenium(II) complexes have generated interest as photosensitizers in PDT due to their favorable photophysical properties compared to other transition metals, including broad absorption spectra and long excited state lifetimes. Moreover, the absorption and emission wavelengths can be precisely tuned through ligand design. Furthermore, ruthenium(II) complexes frequently exhibit high chemical and photochemical stability, allowing them to withstand light without degradation.^21,22^ In this respect, TLD1433 (Figure 1) is the first ruthenium-based PS that has entered a phase II clinical trial to treat noninvasive bladder cancer.^23^ Interestingly, in most of the reports where porphyrins and ruthenium are used, the metal atom is found in the peripheries of the macrocycle, and only a few metaloporphyrins with the ruthenium atom in the central cavity have been described.^24,25^ However, many Ru(II) complexes are poorly soluble in water, which may limit their ability to reach target tissues. Low water solubility can cause aggregation, reduced bioavailability, and ineffective cellular uptake. The clinical applications of these complexes have also been restricted due to their cytotoxicity in the dark and low ROS generation efficiency.^21,26^

It is worth mentioning that PDT is not only used in cancer treatment. For instance, verteporfin (**VP**, Visudyne) is widely used for treating macular degeneration.^27^ However, the use of **VP** has several disadvantages, such as high phototoxicity and slow elimination by the body, meaning that the patient will be photosensitized for more than 48 h after the therapy.^28,29^

Therefore, inspired by the photoproperties of porphyrins and chlorins, a ruthenium(II) ion was introduced in the central cavity of a series of tetrapyrroles to study their potential use as PS for the treatment of cancer, focusing on gastric cancer. Gastric cancer (GC) represents a public health problem due to its high aggressiveness, with a 5-year survival rate of less than 25% and a median survival of about 11 months. It represents the fifth leading cause of cancer-related death in the world, after lung, breast, colon, and liver cancers, with around 769,000 deaths in 2020 (7.7% of all cancers), according to the World Health Organization (WHO).^30^ In terms of incidence, GC is the fifth most common cancer, with more than one million new cases diagnosed in 2020 (5.6% of all cancers).^31,32^ Treatment options are still very limited, and patient management is mainly based on partial or complete gastrectomy combined with oxaliplatin-based (oxaliplatin + 5-Fluorouracyl) neoadjuvant and/or adjuvant chemotherapy. Unfortunately, the efficacy of the treatment is impaired by various resistance mechanisms,^33^ notably due to mutation in p53, which is mutated in up to 70% of GC.^34^ Consequently, there is an urgent need to develop new therapeutic solutions whose mode of action is independent of p53. In this respect, ruthenium-based anticancer compounds have been shown to induce cell death independently of p53, targeting different metabolic pathways and reducing tumor growth *in vivo*.^35−37^ In addition, as the gastric cavity is accessible by endoscopy, combining ruthenium with PDT would be an attractive solution for a localized GC treatment.

## Results and Discussion

### Chemistry

#### Synthesis and Characterization

**Scheme 1:**
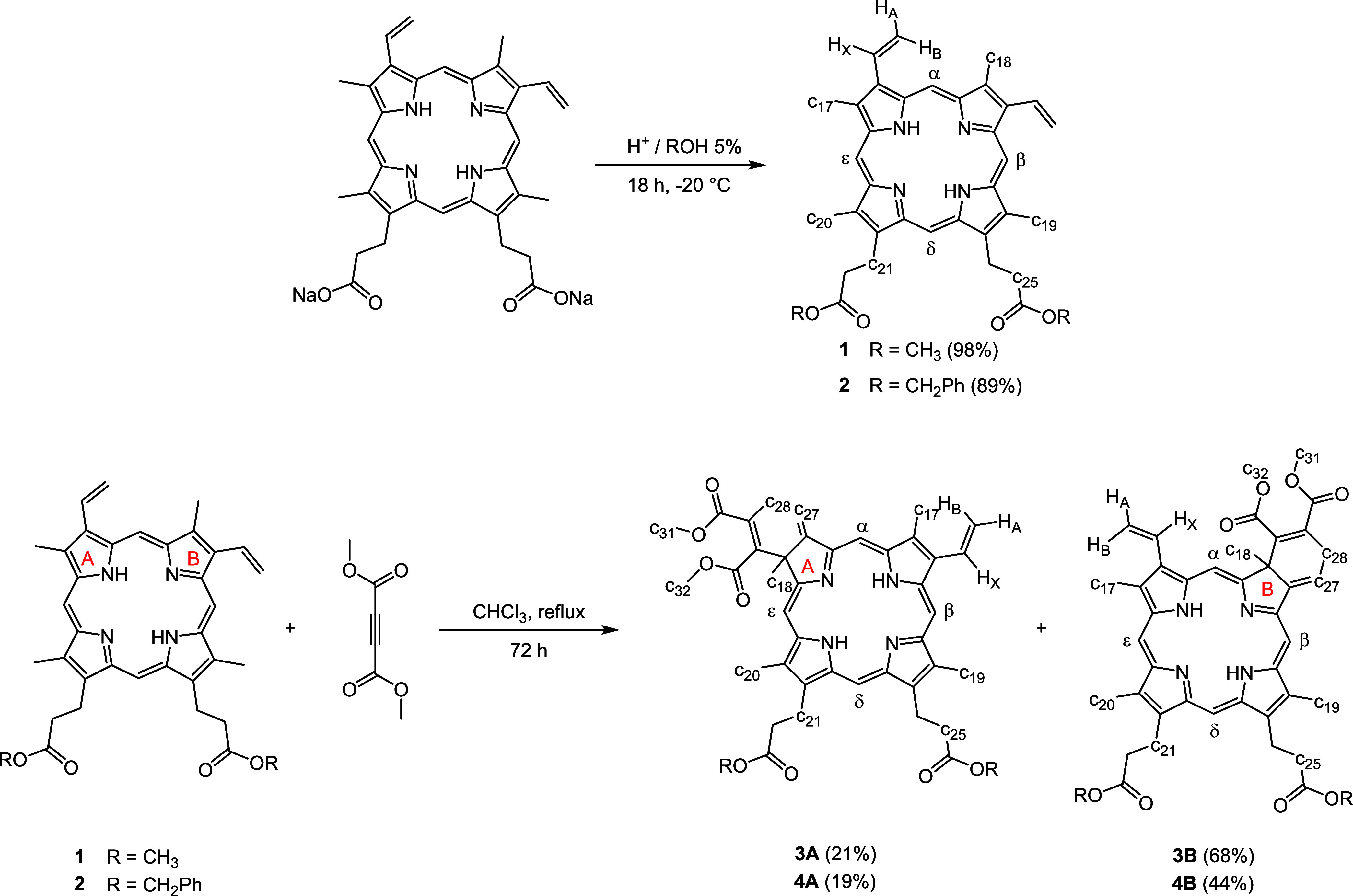
Synthesis of Ligands and Numbering Used for NMR Assignation

The porphyrins **1** and **2** derived from protoporphyrin IX (**PpIX**) were synthesized by adapting a described procedure from commercial **Na**_**2**_**PpIX** salt and methanol or benzyl alcohol in an acidic medium (Scheme 1).^38^

Porphyrins **1** or **2** were used to prepare the chlorins through a Diels–Alder reaction, modifying a reported procedure.^39,40^ Porphyrin **1** or **2** was refluxed with dimethyl acetylenedicarboxylate (DMAD) in dry chloroform for 72 h to form mixtures of **3A** and **3B**, or **4A** and **4B** chlorins (Scheme 1). Isomers **A** and **B** were readily separated by column chromatography on silica gel and characterized by one-dimensional (1D) and two-dimensional (2D)-NMR techniques.

**Scheme 2:**
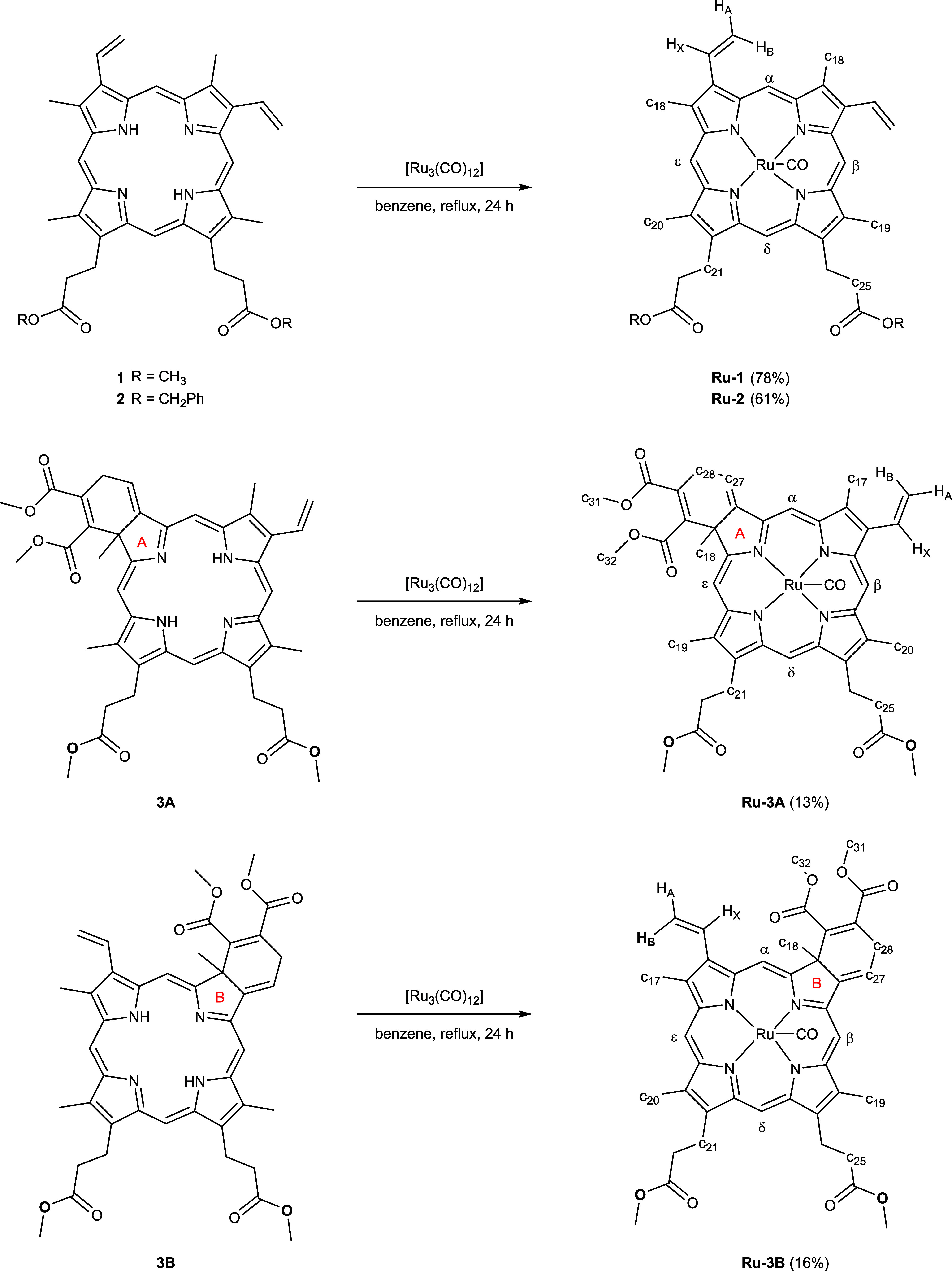
Synthesis of Ruthenium Complexes and Numbering Used for NMR Assignation

The **1** and **2** porphyrins and **3A** and **3B** chlorins were used as ligands to react with [Ru_3_(CO)_12_] to form the new ruthenium(II) complexes. Scheme 2 summarizes the synthesis of the ruthenium derivatives. Two strategies were used to synthesize ruthenium complexes based on chlorins. In the first approach, a mixture of chlorins **AB** was reacted with [Ru_3_(CO)_12_], followed by chromatographic separation of the resulting ruthenium complexes. However, the reaction yield was low, and the separation of the isomers was problematic. Therefore, the second methodology consisted of starting from the purified **3A** or **3B** isomers. Ruthenium complexes were obtained in 13 and 16% yields from the isolated isomer, respectively. The **4A** and **4B** ligands were not metalated as they were obtained in very low quantities. All the new ruthenium(II) derivatives are stable in air and light in the solid state and solution.

All the new compounds were characterized by IR, HR-MS, ^1^H and ^13^C NMR, DEPT-135, HSQC, and HMBC, and their purity was confirmed by HPLC (Figures S1–S61). In addition, crystals suitable for single-crystal X-ray diffraction crystallography were obtained for **Ru-1** and **3B**.

#### Crystallography

**Figure 2:**
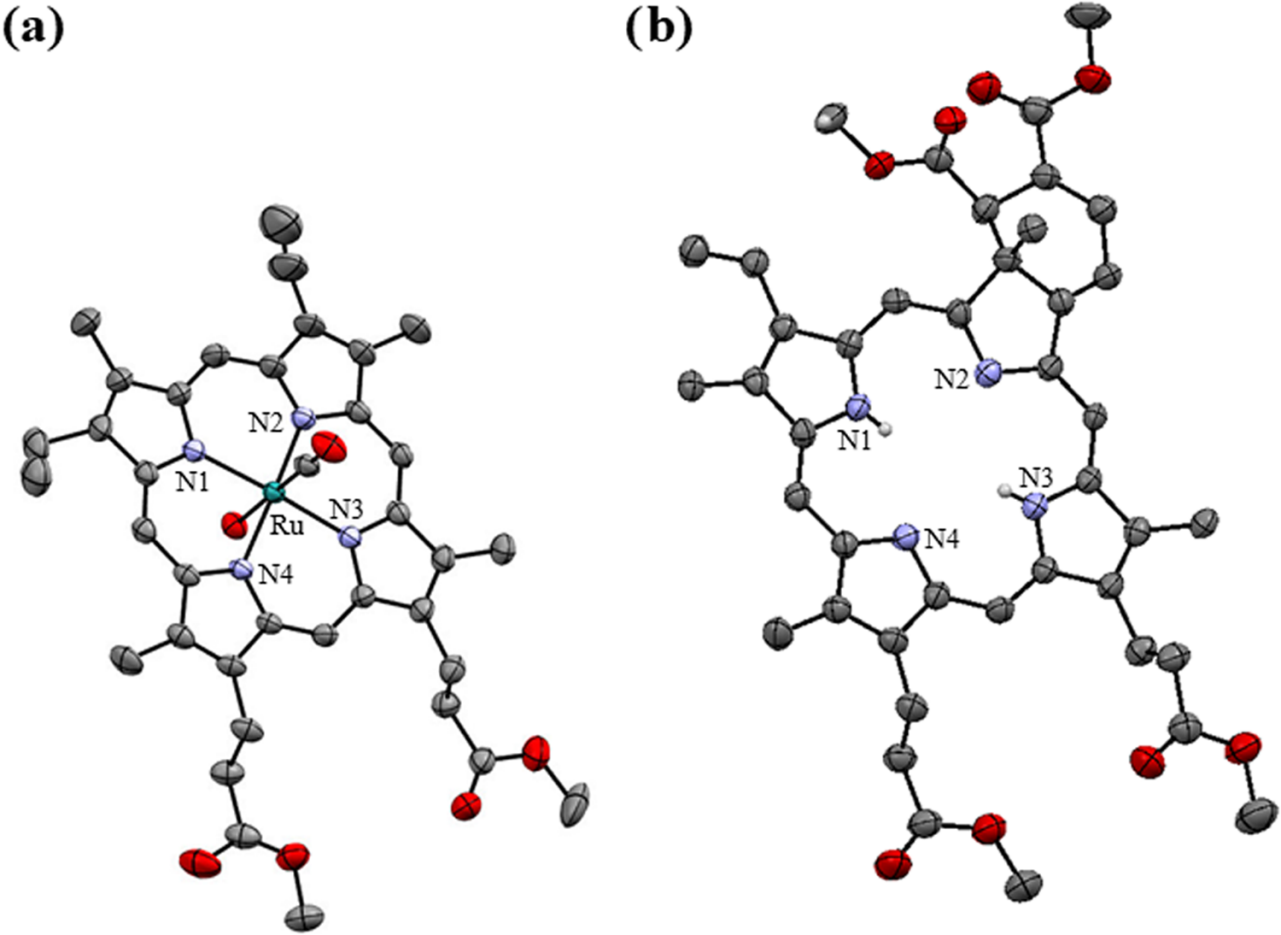
ORTEP diagrams (**a**) **Ru-1** and (**b**) **3B**. Thermal ellipsoids are drawn with a 40% probability level. Hydrogen atoms are omitted for clarity, except for N–H hydrogen atoms.

**Ru-1** and **3B** crystals were obtained by slow evaporation of a hexane/dichloromethane solution. Figure 2 shows the ORTEP diagrams of the structures, and crystallographic data are summarized in Table S1.

Compound **3B** crystallized in an orthorhombic system with space group *Fdd2*, and the structure confirms the cycloaddition. The bond distance between N–H1 and N–H2 is 0.737 and 0.701 Å, respectively. In addition, it is observed that the central structure of the chlorin is planar. **Ru-1** complex crystallized with a water molecule (probably from the solvents used for crystallization) in the *C*2/*c* space group. It has been reported that in similar porphyrin complexes, water, methanol, and ethanol molecules can weakly bind to the metal center *trans* to the CO ligand.^41,42^ The angle between the H_2_O–Ru–C(O) atoms is 179.04°, while the angles between the nitrogen atoms of porphyrin and ruthenium atom are 173.72 and 173.83°, indicating that the structure exhibits a slightly distorted octahedral geometry. The bond distances between Ru-CO and Ru–OH_2_ are 1.782(5) and 2.224(4) Å, respectively. The bond distances between Ru–N1, Ru–N2, Ru–N3, and Ru–N4 are 2.051, 2.057, 2.050, and 2.059 Å, respectively.

#### Electronic Absorption

**Figure 3:**
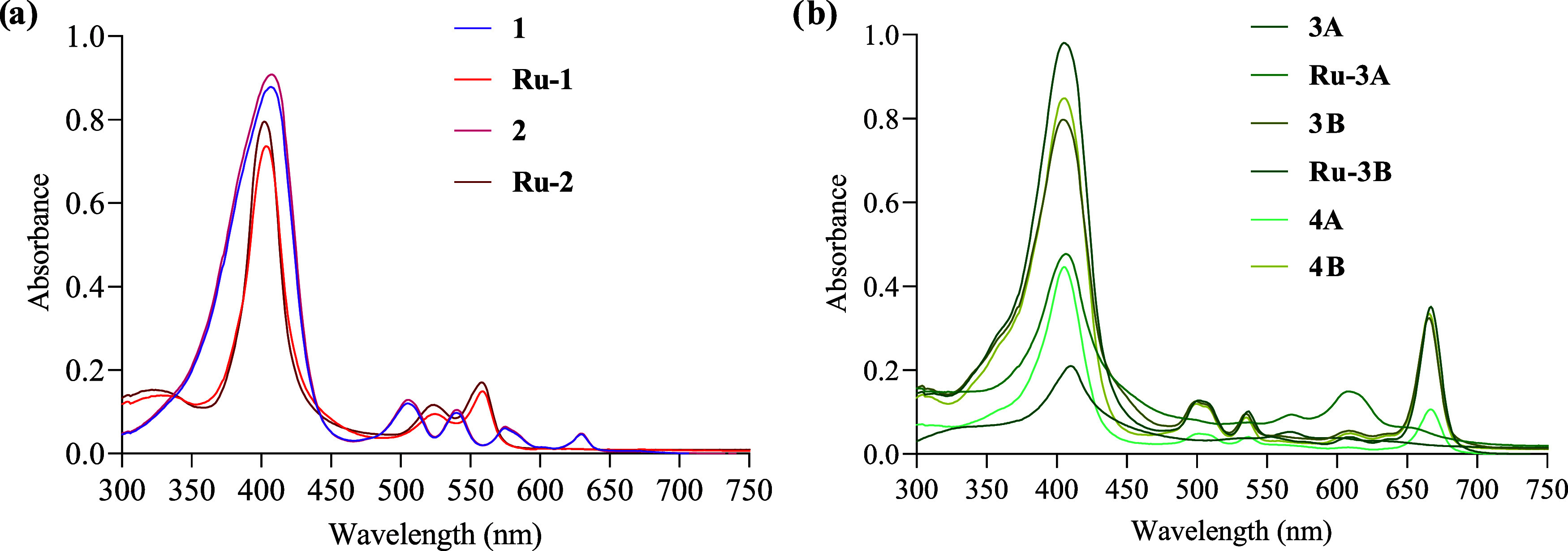
Absorption spectra of (a) porphyrins and metaloporphyrins and (b) chlorins and metalochlorins in DMSO, 1 × 10^–5^ M at 37 °C.

The ultraviolet–visible (UV–vis) absorption spectra of the ligands and complexes were measured in dimethyl sulfoxide (DMSO) at 37 °C at a 1 × 10^–5^ M. Figure 3 shows the spectra for the ligands and complexes, and electronic absorption data are given in Table S2.

A strong Soret absorption band around 400 nm is observed in all the UV–vis spectra, indicating π–π* transitions (Figure 3).^43^ For the ligands, four Q bands in the visible region are attributed to an π-π* electronic transition (a_1u_ → e_g_*) arising from the ground state (S_0_) to the second excited state (S_2_).^44^ For the metaloporphyrinoid derivatives, the bands around 500 to 600 nm are assigned to charge transfer transitions, π-dπ between the tetrapyrrole ring and the metal. In the complexes, the decrease in the number of Q bands is ascribed to a change in the symmetry, from D_2_h to D_4_h, due to the deprotonation and cation formation.^43,44^

#### Stability

The stability of the compounds was evaluated by UV–vis spectroscopy. Figures S62–S70 show the absorption spectra of each compound in DMSO for 24 h at 37 °C at a concentration of 1 × 10^–5^ M. The stability was also evaluated in PBS containing 0.1% DMSO (1 × 10^–5^ M) at 37 °C for 8 h. In DMSO, practically no decomposition of the compounds was observed (Figure 4a). However, in PBS/DMSO (0.1%), a slight decomposition was noted (between 1–10%), as seen in Figure 4b. Figures S71–S79 show the absorption spectra of each compound in PBS/DMSO (0.1%). However, in the case of compound **Ru-2**, the decomposition was more important (48% in PBS/DMSO), as observed in Figure S73.

**Figure 4:**
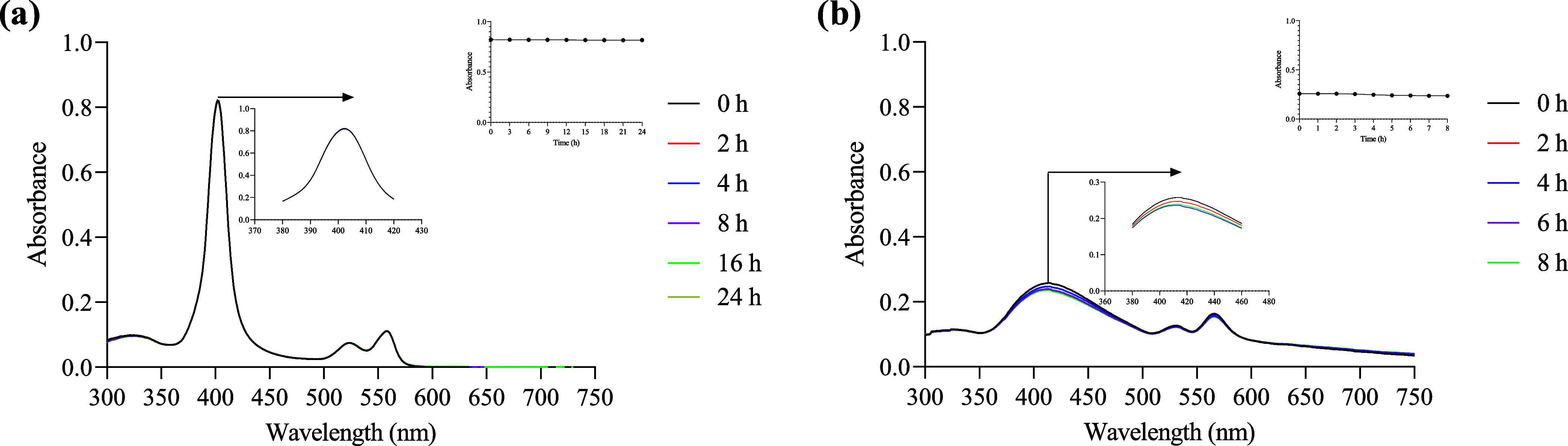
Stability assay for **Ru-1** in (a) DMSO and (b) PBS/DMSO 0.1%. Inset: plot of absorbance *vs* time.

#### Photobleaching

Degradation of the molecules upon light irradiation can cause problems in applications such as photodynamic therapy.^45^ Photodegradation is an oxidative degradation of a molecule over time into fragments of lower molecular weight, implicating that the molecule may lose its photodynamic activity during irradiation.^46,47^

**Figure 5:**
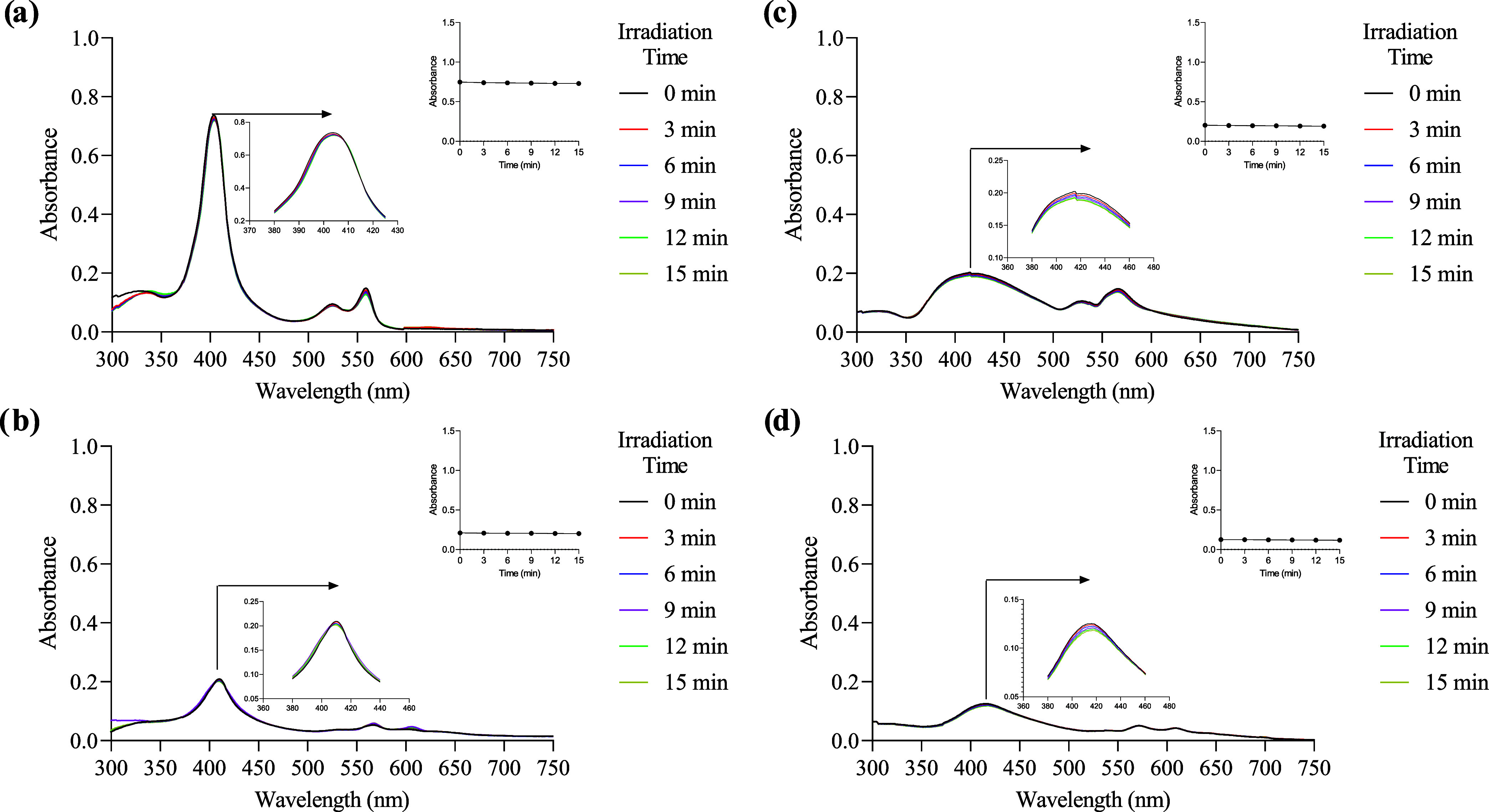
Photodegradation of compound **Ru-1** (a, c) and **Ru-3B** (b, d) in DMSO and PBS/DMSO (0.1%) upon irradiation with white light (50% intensity of a 12 W lamp). Inset: plot of absorbance *vs* time.

The measurement of light tolerance and stability of each compound was studied by UV–vis spectroscopy at 37 °C at 1 × 10^–5^ M. The cell was irradiated with white light, and a spectrum was recorded every 3 min until a total of 5 measurements (15 min). Figure 5 shows the spectra obtained during **Ru-1** and **Ru-3B** photobleaching in pure DMSO (Figure 5a) and PBS/DMSO (0.1%) (Figure 5b). Spectra for the remaining compounds are shown in Figures S80–S87. In all cases, only a slight decrease (1–5%) in the absorption of the Soret band (404–410 nm) was observed.

#### Fluorescence and Singlet Oxygen Formation Quantum Yield Measurements

Emission spectra were measured in DMSO. The free ligands were excited at 503 nm, while the ruthenium complexes were excited at 573 nm. The spectra are shown in Figures S88–S93. For the calculation of fluorescence quantum yield, verteporfin (Φ_f_ = 0.0085) was used as a reference.^46^ The results are shown in Table S2. In all metal-free compounds, an intense emission band at approximately 630–670 nm and a small band at approximately 700–750 nm were observed, assigned to Q (0–0) and Q (1–0) transitions, respectively.^48^ On the other hand, the new ruthenium(II) complexes showed no emission. A similar behavior has been observed in palladium(II) and nickel(II) metaloporphyrins, where the fluorescence was quenched due to electron or energy transfer.^49^

**Figure 6:**
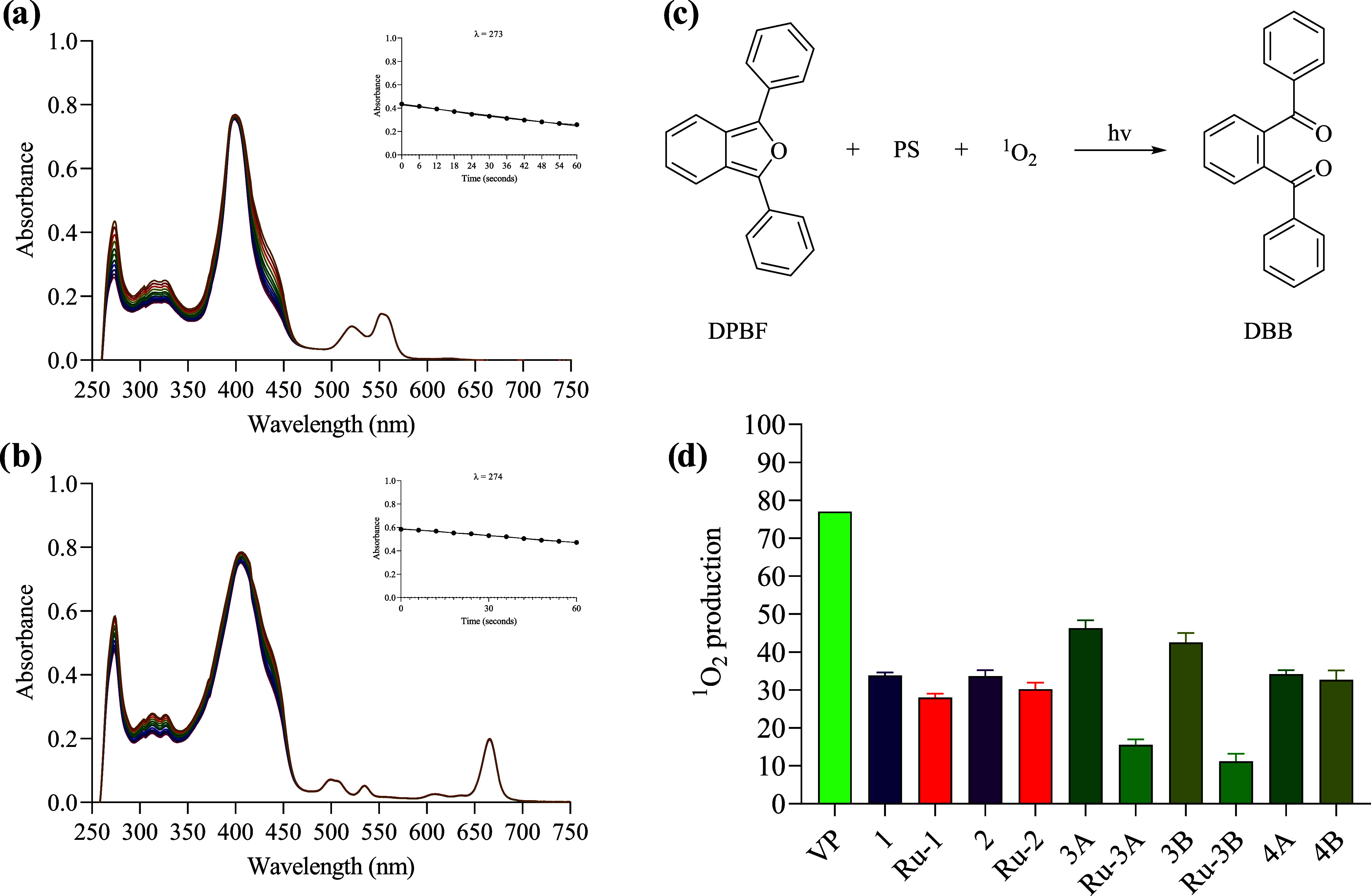
Absorption changes during the determination of singlet oxygen quantum yield of **Ru-1** (a) and **3B** (b) using DPBF as singlet oxygen trapping in DMSO equilibrated with air (inset: plot of absorbance at 214 nm *vs* irradiation time). (c) Reaction between DPBF and photosensitizer in the presence of light and oxygen. (d) Percentage singlet oxygen formation quantum yield relative to verteporfin (Φ_Δ_ = 0.77^46^). [PS] = 7 × 10^–6^ M; [DPBF] = 2 × 10^–5^ M, *N* = 3.

An indirect method was used to calculate the singlet oxygen production quantum yield of all compounds using 1,3-diphenylisobenzofuran (DPBF), which in the presence of ^1^O_2_ oxidizes to 1,2-dibenzoylbenzene (DBB) (Figure 6c). Verteporfin was selected as a reference (Φ_Δ_ = 0.77 in DMSO).^46^ The spectral change of the absorbance band at 214 nm of the DPBF scavenger was monitored to determine the singlet oxygen generation. Eleven measurements were made in a spectrophotometer, one measurement every 6 s after irradiating the samples with white light, for a total of 60 s. Figure 6d summarizes the data obtained.

In all cases, the absorbance of DPBF at 214 nm decreased in the presence of the compounds, confirming the generation of singlet oxygen. All the compounds, but **Ru-3A** and **Ru-3B**, were highly efficient in generating singlet oxygen, with quantum yields between 30 and 50%. Interestingly, for **Ru-3A** and **Ru-3B**, the singlet oxygen quantum yields were much lower (15% and 11%, respectively) than for the corresponding free ligands (**3A** and **3B**) and the other ruthenium complexes (Figure 6d).

#### Determination of ROS Production

**Figure 7:**
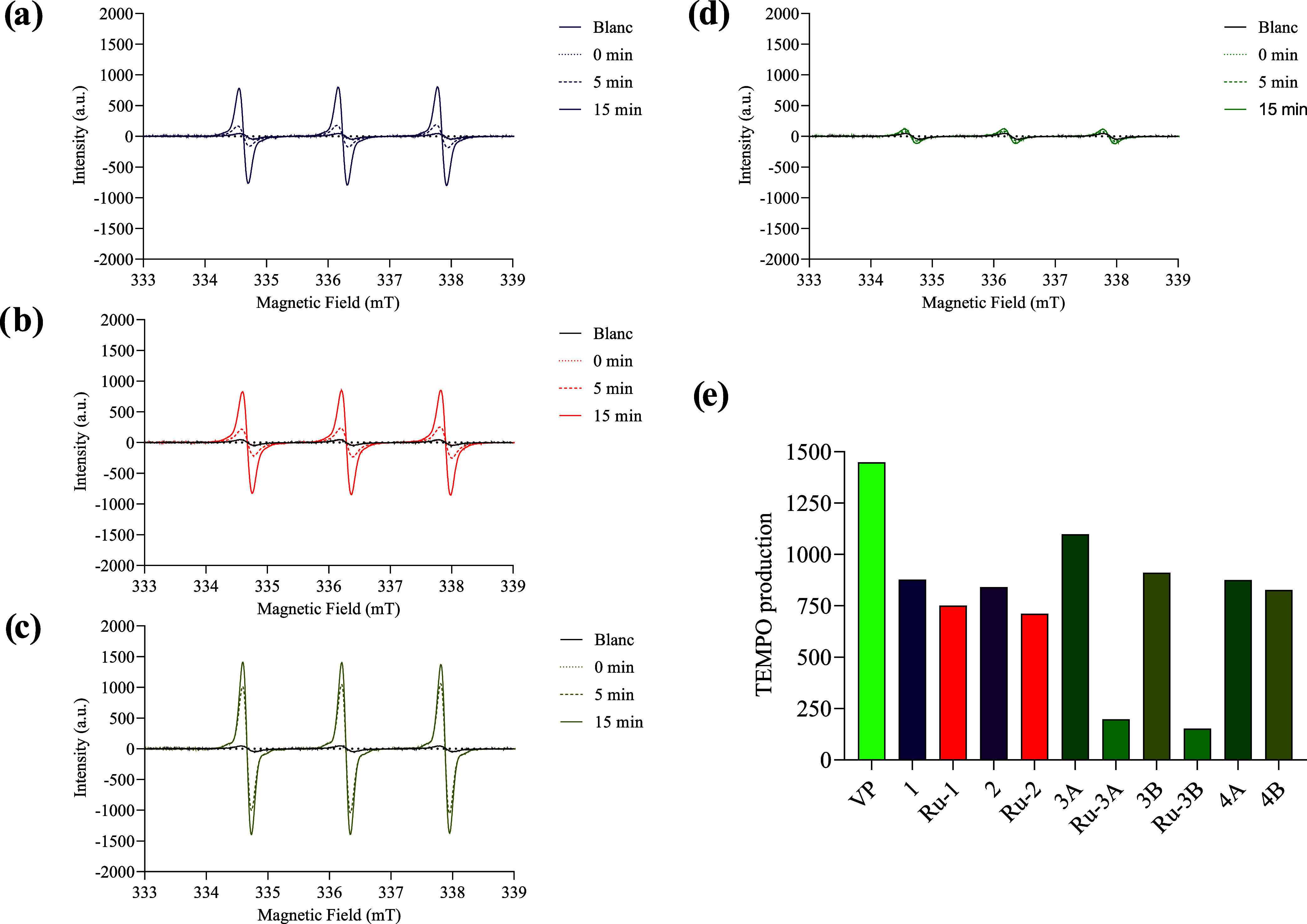
EPR spectra upon irradiation with white light of the PS in the presence of TEMP spin trap in ethanol/DMSO at room temperature: (a) **1**, (b) **Ru-1**, (c) **3B**, and (d) **Ru-3B**. (e) TEMPO signal intensity after 15 min of irradiation. [PS] = 1 × 10^–3^ M, [TEMP] = 4 × 10^–3^ M, blanc = spectra of TEMP without PS.

Since singlet oxygen has a very short half-life, a spin trap and detection of the generated radical by electron paramagnetic resonance (EPR) were used as an indirect method to monitor its formation. The spin trap of choice was 2,2,6,6-tetramethylpiperidine (TEMP), as it can readily be converted into the stable radical 2,2,6,6-tetramethyl-1-piperidinyloxy (TEMPO) in the presence of singlet oxygen. The formation of TEMPO was monitored by continuous white light irradiation of the PS *in situ* for 15 min. As seen in Figure 7a–d, the characteristic triplet signal (*g* = 2.0063 and hfcc = 1.63 mT) for TEMPO was detected for all compounds. However, as previously observed, the production of ^1^O_2_ by the metalochlorins **Ru-3A** and **Ru-3B** was much lower (Figure 7e).

**Figure 8:**
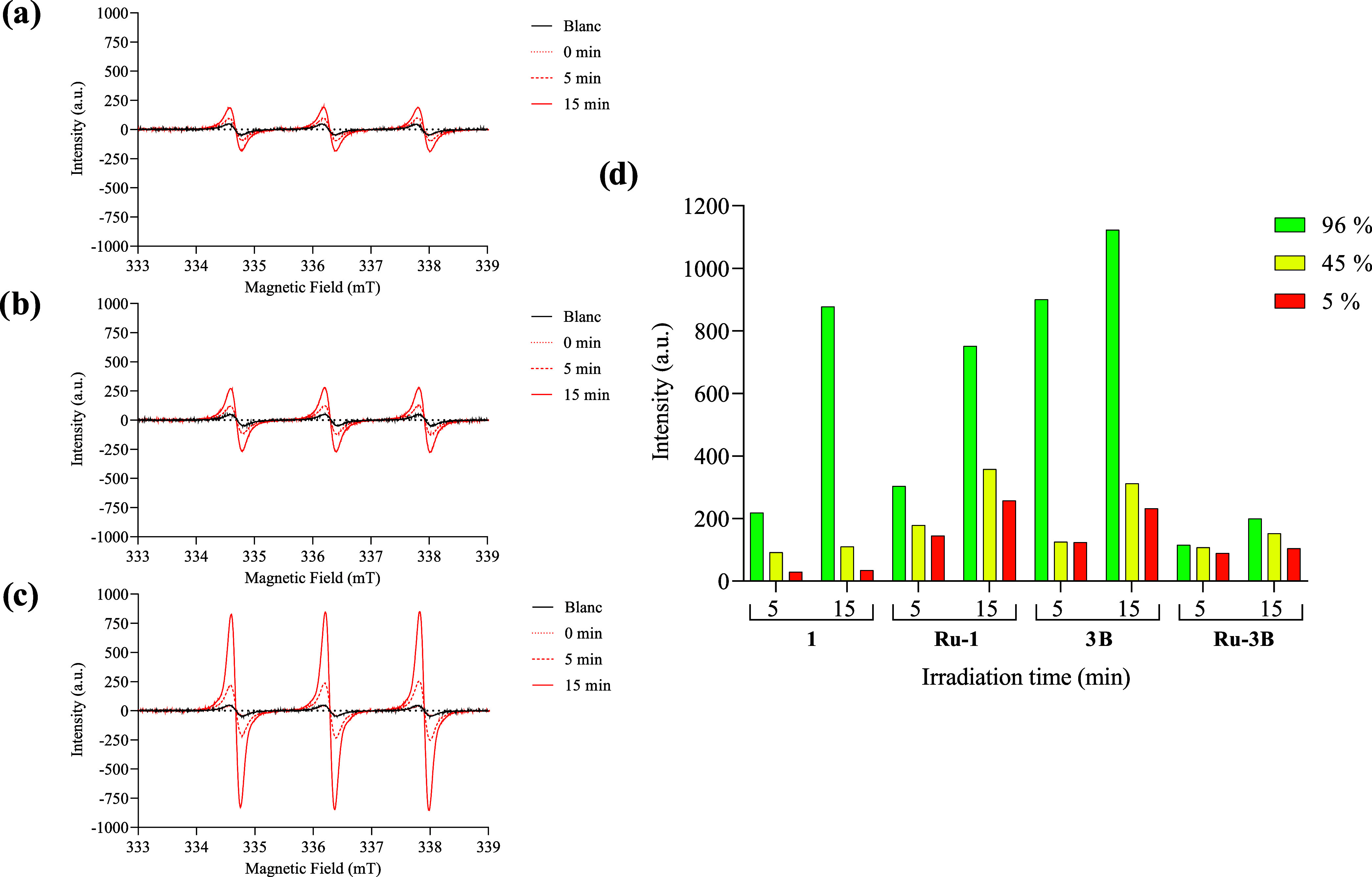
EPR spectra upon irradiation for 5 and 15 min of **Ru-1** in the presence of TEMP varying the oxygen concentration: (a) 5% of oxygen, (b) 45% of oxygen, (c) 96% of oxygen. (d) TEMPO signal intensity for compounds **1**, **Ru-1**, **3B**, and **Ru-3B** at 15 min of irradiation at different oxygen concentrations. [PS] = 1 × 10^–3^ M, [TEMP] = 4 × 10^–3^ M, blanc = spectra of TEMP without PS.

Because our compounds were producing ^1^O_2_ under normal conditions, the effect of the oxygen concentration in the samples was evaluated. Compounds **1**, **Ru-1**, **3B**, and **Ru-3B** were selected for this experiment. Interestingly, compound **Ru-1** produced significant amounts of ^1^O_2_ at low oxygen concentrations, which might indicate that it could potentially be active in type II PDT on hypoxic cancer cells.^50^Figure 8 shows the EPR spectra obtained at different O_2_ concentrations for **Ru-1**, and the spectra for compounds **1**, **3B**, and **Ru-3B** are shown in Figures S94–S96, respectively.

**Figure 9:**
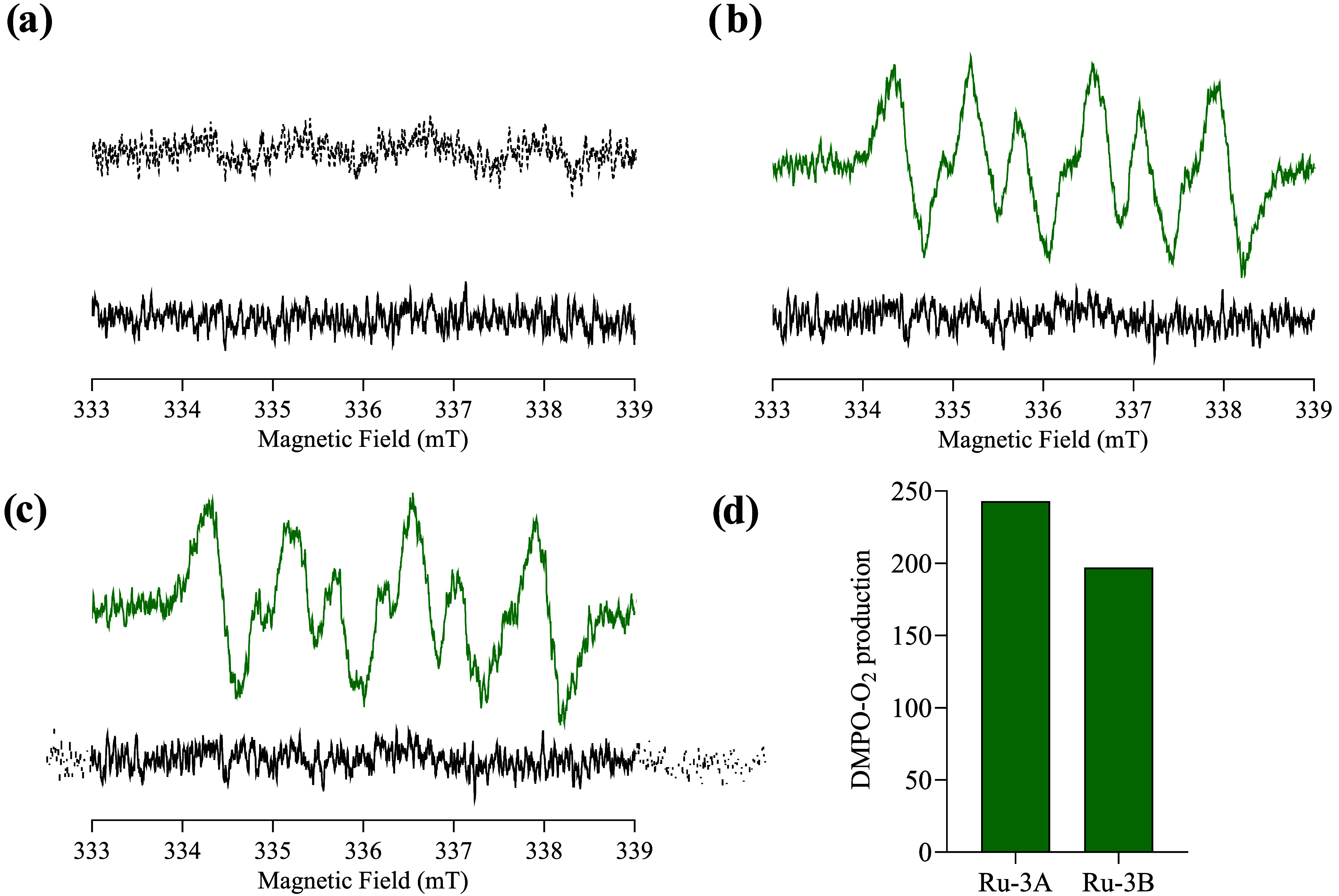
EPR spectra in methanol/DMSO at room temperature: (a) DMPO in the dark (solid line) and upon irradiation (dotted line), (b) **Ru-3A** in the dark (black) and upon irradiation (green), and (c) **Ru-3B** dark (black) and upon irradiation (green). (d) DMPO- O_2_^•–^signal intensity after 15 min of irradiation. [PS] = 1 × 10^–3^ M, [DMPO] = 0.07 M. Irradiation time = 15 min.

Contemplating that type I PDT can also occur when the PS is irradiated, the possibility that the metalochlorins **Ru-3A** and **Ru-3B** can generate ROS different from ^1^O_2_ was investigated. Therefore, another spin trap, 5,5-dimethyl-1-pyrroline-*N*-oxide (DMPO), able to react with superoxide radical O_2_^•–^ was used. Figure 9a–c show the EPR spectra obtained in the presence of DMPO before and after irradiation with white light for 15 min. As expected, no ROS is produced without the PS (Figure 9a). When the sample containing the PS (**Ru-3A** or **Ru-3B**) and DMPO was irradiated with white light for 15 min, the characteristic signal and coupling constants for the DMPO-^–^ O_2_^•–^ adduct (*g* = 2.0060, hfcc = 1.330 mT) were observed (Figure 9b,c).^51,52^ Such results confirm that our metalochlorins can exhibit combined mechanisms of action for PDT, *i.e*., they can produce singlet oxygen in the type II mechanism, as well as the superoxide radical in a type I mechanism. The Soret band of **Ru-3A** and **Ru-3B** metalochlorins showed a lower absorption intensity than for ligands **1**, **2**, **3A**, and **3B**, and complexes **Ru-1** and **Ru-2**. A higher Soret band absorption intensity usually indicates a larger gap between the highest occupied molecular orbital (HOMO) and lowest unoccupied molecular orbital (LUMO), while a lower Soret band absorption intensity means a smaller HOMO–LUMO gap.^53,54^ According to the literature, a small HOMO–LUMO gap facilitates the intermolecular transfer of photoinduced electrons from the excited triplet state to the ground state to produce the superoxide radical, as observed in our study.^55^ However, theoretical calculations in the excited states are necessary to fully explain why the metalochlorins specifically produce superoxide radicals.

#### Lipophilicity

The *n*-octanol/water partition coefficient (log *P*_o/w_) is a well-established parameter that helps to predict the biological activity of drugs and other biologically active compounds.^56^ The so-called “shake-flask” method was adopted to determine the partition coefficient using 1-octanol and PBS (phosphate-buffered saline) as solvents.^57^ The results are summarized in Table 1. All the porphyrin derivatives presented low lipophilicity values. At the same time, the chlorin compounds were much more lipophilic as expected due to the reduction of one of the pyrrole rings and the formation of the new ring in the chlorin.

**Table 1:** Partition Coefficient (log *P*_o/PBS_) of Compounds^a^

compound	log *P*_O/PBS_
**1**	0.957 ± 0.056
**Ru-1**	1.014 ± 0.058
**2**	1.063 ± 0.142
**Ru-2**	1.032 ± 0.108
**3A**	1.478 ± 0.083
**Ru-3A**	1.396 ± 0.051
**3B**	1.661 ± 0.088
**Ru-3B**	1.510 ± 0.177
**4A**	1.368 ± 0.042
**4B**	1.682 ± 0.176
**VP**	1.374 ± 0.064

a The value obtained is the average of three independent experiments at 27 °C.

### Biological Assays

#### Impact of Porphyrins, Chlorins, and Ru(II) Compounds on Gastric Cancer Cell Survival

**Figure 10:**
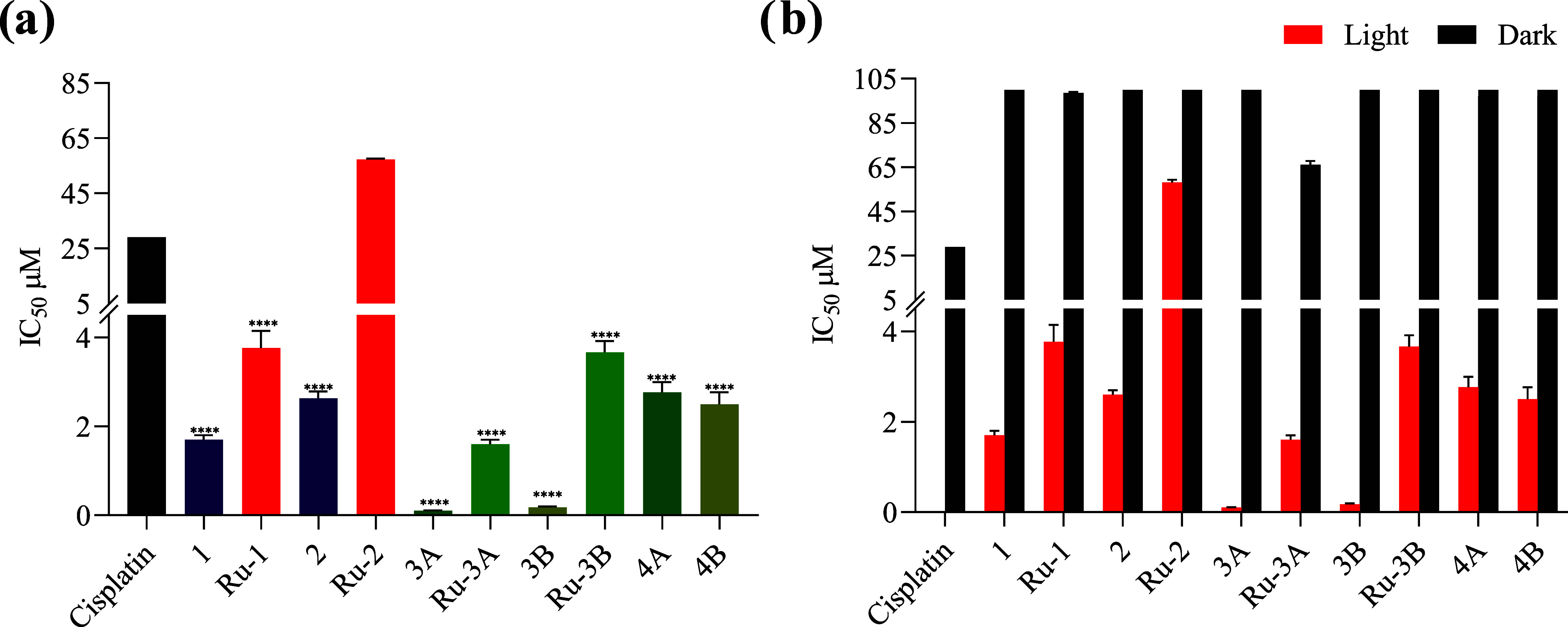
(a) Graphs showing the mean IC_50_ inhibitory concentrations presented in Table 2. Student’s *t*-test is considered significant at *p* < 0.5. * < 0.05, ** < 0.01, *** < 0.001, **** < 0.0001 with respect to cisplatin. (b) Comparison of the minimum inhibitory concentration in the dark and activated under irradiation with white light for 15 min.

To assess the anticancer potential of compounds, the cytotoxicity was evaluated on the gastric cancer cell line AGS. The cells were treated in the dark for 8 h with increasing concentrations of the different compounds (Table 2). Cisplatin was used as a positive cytotoxic and clinically used drug control, and verteporfin (**VP**) or protoporphyrin-IX (**PpIX**) were used as positive PDT controls. Cells were then irradiated for 15 min with white light or kept in the dark and further cultivated for 48h in the dark. An MTT test was then performed to evaluate the impact of the different treatments on cell survival. This demonstrated that in the dark, and, in contrast to cisplatin (IC_50_ = 29 μM) and verteporfin (IC_50_ = 7 μM), all compounds displayed very low toxicity on AGS cells, with an IC_50_ which could not be calculated, except for **Ru-1** (IC_50_ = 95 μM) and **Ru-3A** (IC_50_ = 64 μM) (Table 2, Figures 10, and S97–S101). After irradiation, all compounds showed a marked increase in their toxicity, ranging between 0.1 and 4 μM, which, except for **Ru-2** (IC_50_ = 51 μM), were below the IC_50_ of cisplatin (Figure 10 and Table 2). The difference in the observed toxicity is most likely not due to a difference in cellular uptake, as there does not appear to be a correlation between their lipophilicity and the IC_50_ values. The lower cytotoxicity of **Ru-2** is most likely due to its low stability in biological media, as determined by stability tests in PBS/DMSO. The phototoxicity index (PI) was calculated based on the IC_50_ dark/light ratio, and values as high as 1000 and 555 were calculated for compounds **3A** and **3B**, respectively.^58^

**Table 2:** Mean Inhibitory Concentrations (IC_50_) of the Compounds in AGS Gastric Cancer Cells^a^

	IC_50_ (μM)		
compound	light	dark	PI
**cisplatin**		29	
**PpIX**	0.5	>100	>200
**Vp**	0.003	7	=13,270
**1**	1.7 ± 0.184	>100	>17
**Ru-1**	3.8 ± 0.379	95 ± 0.462	=25
**2**	2.6 ± 0.100	>100	>18
**Ru-2**	51.3 ± 1.249	>100	>2
**3A**	0.1 ± 0.06	>100	>1000
**Ru-3A**	1.7 ± 0.100	64.1 ± 0.815	=38
**3B**	0.2 ± 0.021	>100	>555
**Ru-3B**	3.2 ± 0.252	>100	>31
**4A**	2.5 ± 0.231	>100	>40
**4B**	2.8 ± 0.265	>100	>36

a Cells were incubated for 8 h, followed by irradiation with white light for 15 min or kept in dark condition. The MTT assay was performed to determine cell viability after 48 h of treatment. *N* = 3.

Given these results, compounds **1**, **Ru-1**, **3B**, and **Ru-3B** were selected as lead compounds for further studies. **Ru-3B** complex is of special interest as it could present different PDT mechanisms, combining the production of ^1^O_2_ by the type II mechanism and O_2_^•**–**^ radicals by a type I mechanism (as mentioned above). This capacity might be of significant importance for the design of new PS since the production of O_2_^•**–**^ radicals does not directly depend on the concentration of oxygen present in the hypoxic microenvironment of cancer cells.^59−61^

#### Induction of Cell Deaths by Porphyrins, Chlorins, and Ruthenium Compounds

**Figure 11:**
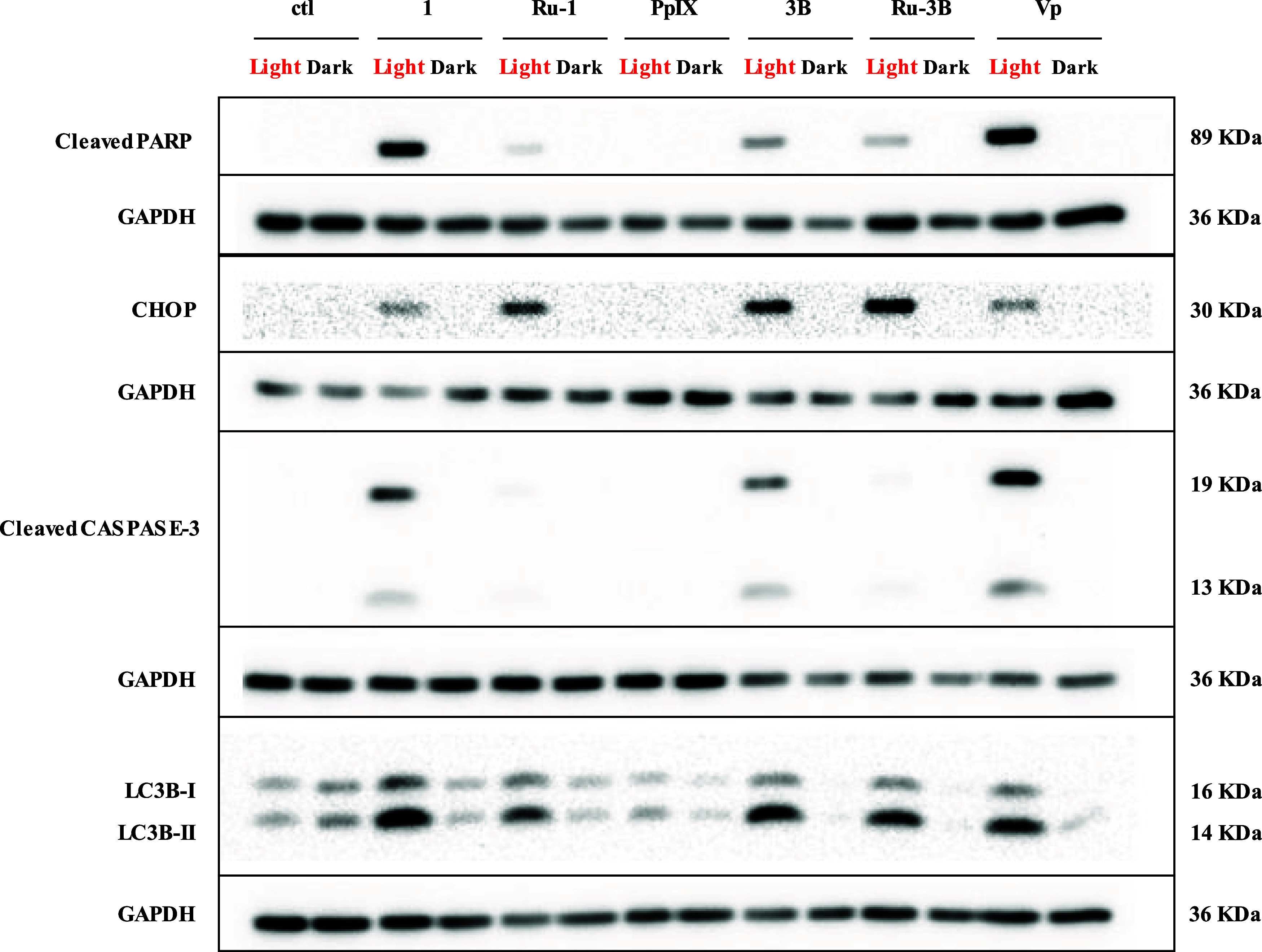
Effects of photoactivation of **1**, **Ru-1**, **3B**, and **Ru-3B** on protein expression on different cell death markers in AGS gastric cancer cells. AGS cells were treated with **1**, **Ru-1**, **3B**, **Ru-3B, PpIX**, or **VP** at IC_50_ concentrations for 4 h and then either irradiated with white light or kept in the dark for 15 min and then further cultivated for 24 h in the dark. Protein extraction was performed, and expression of the different markers was determined by Western blot. GAPDH was used as a loading control. Representative Western blot of three independent experiments. ctl = control, untreated cells.

The therapeutic effects of PDT treatment on cancer cells are thought to be mainly related to ROS production, which are highly reactive and cytotoxic species that can induce cell death by apoptosis.^62^ It has also been shown that ruthenium compounds can induce different cell death pathways.^63,64^ For these reasons, we initially analyzed by Western blot the expression of cleaved caspase 3 and PARP, which are two well-known markers for programmed cell death apoptosis.^65−70^ For this, AGS cells were treated at the IC_50_ of each compound for 4 h in the dark and then irradiated, or not, with white light for 15 min. The cells were then further cultivated in the dark for 24 h. Figure 11 shows that, except for **PpIX**, all compounds induced the cleavage of caspase 3 and PARP after irradiation, notably compound **1**, which was as efficient as **VP**. Then, the protein expression of CHOP, a key component of the endoplasmic reticulum (ER) stress-mediated apoptosis pathway and known to be induced by ruthenium compounds, was evaluated.^71,72^ All compounds, except for **PpIX**, induced the expression of CHOP upon irradiation. Interestingly, CHOP expression was much more strongly induced by the ruthenium compounds compared to their respective free ligands or verteporfin, suggesting that they possibly lead to a much stronger accumulation of misfolded or unfolded proteins and/or ROS production (Figure 11).^73,74^ Furthermore, CHOP has been reported to participate in the regulation of ER stress-mediated autophagy.^75−77^ Autophagy is a cellular self-degradation system that facilitates the degradation of damaged organelles and misfolded or mutant proteins.^78^ In cancer, autophagy protects tumor cells during cancer progression. However, it can also suppress cancer cell development or induce cell death.^79,80^ Autophagy is characterized by the formation of autophagosomes, which express LC3B proteins. To investigate this, the expression and conversion of LC3B–I to LC3B–II was analyzed by Western blot (Figure 11). The results show that following irradiation, all ruthenium compounds induced the expression of LC3B–I and LC3B–II (Figure 11). Interestingly, upon light activation, **3B** and **Ru-3B** led to a marked induction of LC3B–I/II expression, similar to verteporfin, suggesting a specific induction of autophagy (Figures 11 and S102)

**Figure 12:**
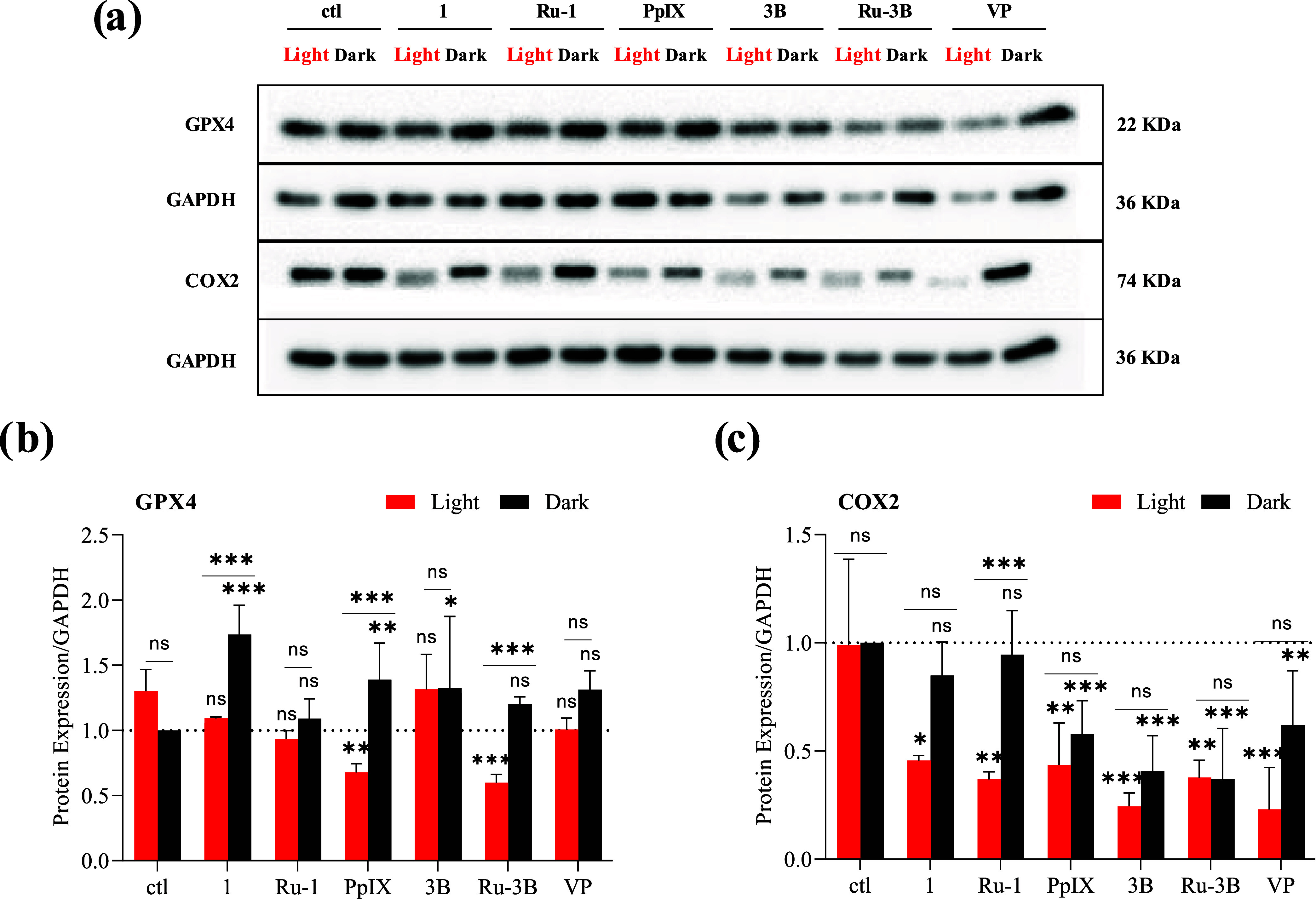
Effects of photoactivation of **1**, **Ru-1**, **3B** and **Ru-3B** on protein expression of ferroptosis and inflammation markers in AGS gastric cancer cells. AGS cells were treated with **1**, **Ru-1**, **3B**, **Ru-3B, PpIX**, or **VP** at IC_50_ concentrations for 4 h and then either irradiated with white light or kept in the dark for 15 min and then further cultivated for 24 h in the dark. (a) Expression of the different markers was determined by Western blot. GAPDH was used as a loading control. Representative Western blot of three independent experiments. ctl = control, untreated cells. Graphs represent the quantification of three independent experiments of GPX4 (b) and COX2 (c) expression, compared to not-treated AGS cells (ctl) and normalized to GAPDH expression. Bars above the colons indicate the comparison of dark *versus* light for each compound. The dark and light results of each compound were compared to those of the respective untreated control condition (ctl). Statistical analysis: 2-way Anova *<0.05, **<0.01, ***<0.001, ns = not-significant.

Ferroptosis, another form of nonapoptotic cell death, can promote inflammation and is triggered by iron-catalyzed lipid peroxidation *via* Fenton-type reactions, where the main ROS responsible for lipid damage are hydroxyl radical and superoxide radical.^81,82^ Glutathione peroxidase (GPX4) can suppress lipid peroxidation and protect cells from ferroptosis.^83^ To investigate the impact of the different compounds on GPX4 expression, AGS cells were treated as described above, and then GPX4 expression was analyzed by Western blot (Figure 12a). This showed that upon activation by light, **PpIX** and **Ru-3B** lead to a decrease in GPX4 expression (Figure 12a,b), which suggests that these two compounds might induce ferroptosis.

COX2 is known for its central role in inflammation and prostaglandins (PGs) production. In normal cells, COX2 expression is almost absent, whereas in cancer cells, it is frequently overexpressed.^84^ Importantly, it has been shown that in cancers, COX2 is not only a mediator of inflammation but could also be a mediator of oxidative stress and inhibition of apoptosis, as well as cancer cell proliferation.^85^ The expression of COX2 was analyzed by Western blot (Figure 12a), showing that upon light irradiation, compounds **1** and **Ru-1** significantly reduce COX2 expression compared to untreated conditions. In contrast, **PpIX**, **3B**, **Ru-3B**, and **VP** already lead to a reduction in COX2 expression in the dark (Figure 12a,c). These results suggest that the effect of **PpIX**, **3B**, **Ru-3B**, and **VP** on cell survival upon light activation may not be due to their repressive influence on COX2 expression.

## Conclusions

A series of tetrapyrrole-ruthenium(II) compounds derived from protoporphyrin IX and the commercial drug verteporfin were designed as potential photosensitizers for PDT. The introduction of the ruthenium atom in the central cavity of the macrocycle did not affect the low toxicity in the dark on human gastric cancer cells compared to the free ligands while displaying remarkable cytotoxicity upon irradiation with light. Additionally, our results show that the complexes could cause cell death under low oxygen concentrations by generating singlet oxygen or superoxide anion radicals. Notably, the ruthenium atom coordinated to the chlorin ligands allowed combined mechanisms since EPR studies showed that both singlet oxygen and superoxide radicals could be produced by complexes **Ru-3A** and **Ru-3B** after irradiation. The ruthenium complex **Ru-1** also produced large amounts of singlet oxygen at very low oxygen concentrations. Such results are significant since these new photosensitizers could be used for PDT in hypoxic environments, as often found in tumors. In addition, the study demonstrates that upon light activation, the ruthenium derivatives not only induce cell death *via* a caspase 3 mediated apoptotic pathway, but also partly *via* CHOP, which is an endoplasmic reticulum (ER) stress-inducible transcription factor involved in the development of apoptosis and growth arrest. These data confirm that the new ruthenium compounds can be tuned to trigger various cell death pathways and highlight the potential of our complexes as promising multitarget therapeutic alternatives that could reduce tumor resistance mechanisms.

## Experimental Section

### Chemistry

#### Reagents and Measurements

The compounds were synthesized using a double vacuum/inert gas line under a nitrogen or argon atmosphere. All solvents were dried using established procedures and distilled under nitrogen prior to use. All reagents were purchased from Sigma-Aldrich and were used without prior purification. RuCl_3_ was purchased from Pressure Chemical Company. Ruthenium precursor [Ru_3_(CO)_l2_] was prepared according to previously reported procedures.^86^

The reactions were monitored by thin layer chromatography (TLC) using silica gel 60 F_254_ alumina plates (Merck). Infrared spectra were recorded on an α ATR spectrometer from Bruker Optics. Mass spectra (FAB^+^) were obtained using a Jeol JMS-SX102A instrument with *m*-nitrobenzyl alcohol as the matrix. Electrospray ionization mass spectrometry (ESI-MS) was performed using a Bruker Esquire spectrometer. The NMR spectra were recorded at room temperature using deuterated chloroform, acetone, or acetonitrile as solvent on a Bruker Advance spectrometer at 300 MHz for proton and 75 MHz for carbon and on a Bruker Advance spectrometer at 500 MHz for proton and 125 MHz for carbon. Chemical shifts (δ) are in ppm downfield of TMS using the residual solvent as an internal standard. Coupling constants (*J*) are expressed in Hz. Multiplicity: s = singlet, d = doublet, t = triplet, sept = septuplet, m = multiplet. The UV–vis absorption spectra were recorded on a Shimadzu 2600 spectrophotometer in a quartz cuvette at 37 °C with 1 × 10^–5^ M solutions of each compound in DMSO (prepared from a 10 mM stock solution). The fluorescence spectra were obtained in an Agilent Varian Cary 100 spectrophotometer in quartz cuvettes at room temperature. Electron paramagnetic resonance (EPR) determinations were carried out in an EPR spectrometer (Jeol JES-TE300), operated in the X-Band mode at a modulation frequency of 100 kHz with a cylindrical cavity (TE_011_). Each sample was placed in a quartz flat cell (Wilmad Glass Company) and irradiated *in situ*. The photogeneration of ^1^O_2_ was carried out with a 500 Hg Arc lamp (Oriel OPS-A500), and the samples were irradiated continuously at 160 W. Data acquisition and manipulation were done using the ES-IPRIT/TE program. The HPLC chromatograms were recorded on an Agilent model 1200 Series Binary SL system with a UV–vis detector (λ = 400 nm) and an Eclipse Plus C_18_ (3.5 μm × 2.1 mm) column. The flow rate was 0.2 mL/min, with the initial mobile phase acetonitrile–water (60:40) for the first 10 min. Then acetonitrile–water (90:10) for 20 min and finally acetonitrile for 20 min.

### Synthesis of the Ligands

#### Synthesis of Porphyrin Derivatives

##### Porphyrin **1**

The esterification reaction was carried out by adapting a reported procedure.^38^ A solution of 95.0 mL of dry methanol and 5.0 mL of H_2_SO_4_ at 5% (v/v) was cooled to −20 °C in a cold bath, and 1.0 g (1.648 mmol) of the disodium salt of the protoporphyrin IX (**Na**_**2**_**-PpIX**) was added. The reaction mixture was stirred overnight at −18 °C in the dark. The solution was neutralized with a 10% aqueous solution of NaHCO_3_, and the organic phase was extracted with 200 mL of CHCl_3_ and dried over Na_2_SO_4_. The solvent was evaporated to dryness under vacuum, and the crude solid was purified by column chromatography (silica gel, (1/10) ethyl acetate/dichloromethane) to give 954 mg of a purple solid in 98% yield. The purity observed by HPLC is >99%.

MS (FAB^+^) *m*/*z* (%) 591 (30) [C_36_H_38_N_4_O_4_ + H]^+^. IR (ATR, cm^–1^): ν–NH 3039, ν_as_–CH_3_ 2913, ν_s_–CH_3_ 2852, ν–C=O (ester) 1731. UV–vis (DMSO, λ_max_ nm) (log ε): λ_1_ (Soret) 406 (4.48), λ_2_ (Q_IV_) 506 (4.43), λ_3_ (Q_III_) 540 (3.33), λ_4_ (Q_II_) 576 (3.12), λ_5_ (Q_I_) 630 (2.97). ^1^H NMR (CDCl_3_, 300 MHz): −3.84 (s, 2H, NH); 3.27 (t, 4H, ^3^*J* = 7.75, H-22 and H-25); 3.59, 3.60, 3.67, and 3.68 (4s, 18 H, H-17, H-18, H-19, H-20, O–CH_3_ and O–CH_3_); 4.38 (t, 4H, ^3^*J* = 7.71, H-21 and H-24); 6.18 (dd, 2H, *J*_AB_ = 1.06, *J*_AX_ = 11.57, H_A_); 6.36 (dd, 2H, *J*_BA_ = 1.05 Hz, *J*_*BX*_ = 17.74 Hz, H_B_); 8.25 (m, 2H, H_X_); 9.97, 10, 10.11, and 10.13 (4s, 4H, H-α, H-β, H-γ and H-δ). ^13^C NMR (CDCl_3_, 75 MHz): 11.81, 12.84, 21.96, 37.06, 51.89, 96.19, 97.18, 97.50, 98.10, 120.90, 130.42, 173.73. The atoms were numbered according to Scheme 1.

##### Porphyrin **2**

The esterification reaction was carried out by adapting a reported procedure.^38^

A solution of 2.0 mL of benzyl alcohol and 0.5 mL of 1.0 N HCl was cooled to −20 °C in a cold bath, and 70 mg (0.115 mmol) of the disodium salt of the protoporphyrin IX (**Na**_**2**_**-PpIX**) was added. The reaction mixture was stirred overnight at −18 °C in the dark. The solution was neutralized with a 10% aqueous solution of NaHCO_3_, and the organic phase was extracted with 100 mL of CHCl_3_ and dried over Na_2_SO_4_. The solvent was evaporated to dryness under vacuum, and the crude solid was purified by column chromatography (silica gel, (6/4) ethyl acetate/dichloromethane) to give 76 mg of a purple solid in 89% yield. The purity observed by HPLC is >99%.

MS (FAB^+^) *m*/*z* (%) 743 (15) [C_48_H_46_N_4_O_4_ + H]^+^. IR (ATR, cm^–1^): ν–NH 3306, ν_as_–CH3 2909, ν_s_–CH3 2854, ν–C=O (ester) 1735. UV–vis (DMSO, λ_max_ nm) (log ε): λ_1_ (Soret) 408 (4.76), λ_2_ (Q_IV_) 506 (3.87), λ_3_ (Q_III_) 540 (3.77), λ_4_ (Q_II_) 576 (3.56), λ_5_ (Q_I_) 630 (3.41). ^1^H NMR (CDCl_3_, 300 MHz): −3.87 (s, 2H, NH); 3.30 (t, 4H, ^3^*J* = 7.55, H-22 and H-25); 3.55 and 3.67 (2s, 12 H, H-17, H-18, H-19 and H-20); 4.38 (t, 4H, ^3^*J* = 7.51, H-21 and H-24); 5.04 (s, 4H, Ph–CH_2_); 6.18 (dd, 2H, *J*_AB_ = 1.01, *J*_AX_ = 11.56, H_A_); 6.37 (dd, 2H, *J*_BA_ = 1.68, *J*_BX_ = 17.80, H_B_); 7.03 (m, 10H, Ph-H); 8.25 (dd, 2H, *J*_XA_ = 11.44, *J*_XB_ = 17.17, H_X_); 9.96, 9.97, 10.07, and 10.13 (4s, 4H, H-α, H-β, H-γ and H-δ). ^13^C NMR (CDCl_3_, 75 MHz): 11.83, 12.87, 21.94, 37.18, 66.51, 96.37, 97.25, 97.56, 98.11, 120.93, 128.07, 128.14, 128.41, 130.44, 135.84, 173.14. The atoms were numbered according to Scheme 1.

#### Synthesis of Chlorin Derivatives

The Diels–Alder reaction was carried out by modifying a reported procedure.^39,40^ Dimethyl acetylenedicarboxylate (1.0 mL, 0.008 mol) was added to a solution of 100 mg of the porphyrin **1** or **2** in 5 mL of chloroform. The reaction mixture was heated to reflux temperature for 72 h with constant stirring. The reaction was concentrated under vacuum, and the two isomers were separated by silica gel chromatography using dichloromethane/hexane/ethyl acetate as eluent (8:1.2:0.8).

##### Chlorin **3A**

Dark blue solid, 19 mg, 21% yield. The purity observed by HPLC is >99%. MS (ESI) *m*/*z* 733 [C_42_H_44_N_4_O_8_+H]^+^. IR (ATR, cm^–1^): ν–NH 3334, ν_as_–CH_3_ 2948, ν_s_–CH_3_ 2856, ν–C=O (ester) 1721. UV–vis (DMSO, λ_max_ nm) (log ε): λ_1_ (Soret) 406 (4.92), λ_2_ (Q_IV_) 502 (4.05), λ_3_ (Q_III_) 536 (3.94), λ_4_ (Q_II_) 608 (3.60), λ_5_ (Q_I_) 666 (4.51). ^1^H NMR (CDCl_3_, 300 MHz): −2.63 (s, 2H, NH); 2.11 (s, 3H, H-18); 3.26 (t, 2H, *J* = 7.79, H-25); 3.24 (t, 2H, *J* = 7.85, H-22); 3.37 (s, 3H, H-19); 3.52 (a, 3H, H-20); 3.54 (s, 3H, H-17); 3.60 (dd, 1H, *J* = 1.90, H-28); 3.68 (s, 3H, O–CH_3_); 3.69 (s, 3H, O–CH_3_); 3.93 (s, 3H, H-31); 4.02 (dd, 1H, *J* = 6.67, H-28); 4.07 (s, 3H, H-32); 4.16 (t, 2H, *J* = 7.61, H-24); 4.36 (t, 2H, *J* = 7.23, H-21); 6.14 (dd, 1H, *J*_AB_ = 1.46, *J*_AX_ = 11.51, H_A_); 6.32 (dd, 1H, *J*_BA_ = 1.43, *J*_BX_ = 17.84, H_B_); 7.39 (dd, 1H, *J* = 1.97, *J* = 6.61, H-27); 8.11 (dd, 1H, *J*_XA_ = 11.49, *J*_XB_ = 17.79, H_X_); 9.13 (s, 1H, H-δ); 9.34 (s, 1H, H-α); 9.74 (s, 1H, H-γ); 9.76 (s, 1H, H-β). ^13^C NMR (CDCl_3_, 75 MHz): 11.54, 11.73, 12.40, 21.71, 21.95, 28.33, 29.95, 36.78, 37.17, 51.82, 51.93, 52.57, 52.73, 54.93, 90.54, 92.44; 99.03, 99.49, 118.39, 121.63, 128.77, 129.76, 129.86, 130.58, 133.19, 133.22, 134.16, 136.26, 137.28, 138.08, 138.26, 140.05, 149.25, 149.35, 150.56, 151.93, 152.22, 162.39, 166.21, 170.28, 173.52, 173.90. The atoms were numbered according to Scheme 1.

##### Chlorin **3B**

Brown solid, 61 mg, 68% yield. The purity observed by HPLC is >99%. MS (ESI) *m*/*z* 733 [C_42_H_44_N_4_O_8_+H]^+^. IR (ATR, cm^–1^): ν–NH 3339, ν_as_–CH_3_ 2957, ν_s_–CH_3_ 2854, ν–C=O (ester) 1717. UV–vis (DMSO, λ_max_ nm) (log ε): λ_1_ (Soret) 404 (5.03), λ_2_ (Q_IV_) 500 (4.07), λ_3_ (Q_III_) 534 (3.93), λ_4_ (Q_II_) 608 (3.62), λ_5_ (Q_I_) 666 (4.55). ^1^H NMR (CDCl_3_, 300 MHz): −2.48 (s, 2H, NH); 2.11 (s, 3H, H-18); 3.20 (2 t, superimposed, 4H; H-22 and H-25); 3.42 (s, 3H, H-20); 3.49 (s, 3H, H-17); 3.62 (dd, 1H, *J* = 1.88, *J* = 21.03, H-28); 3.66 (s, 6H, O–CH_3_); 3.67 (s, 3H, H-19); 3.91 (s, 3H, H-31); 3.98 (dd, 1H, *J* = 6.68, *J* = 21.07, H-28); 4.01 (s, 3H, H-32); 4.17 (t, 2H, ^3^*J* = 7.54, H-21); 4.31 (t, 2H, ^3^*J* = 7.57, H-24); 6.17 (dd, 1H, *J*_AX_ = 11.62, H_A_); 6.34 (dd, 1H, *J*_BX_ = 17.78, H_B_); 7.39 (dd; 1H, ^3^*J* = 6.37, H-27); 8.18 (dd, 1H; *J*_XA_ = 11.55, *J*_XB_ = 17.79, H_X_); 9.22 (s, 1H; H-α); 9.31 (s, 1H, H-β); 9.66 (s, 1H, H-γ); 9.80 (s, 1H, H-δ). ^13^C NMR (CDCl_3_, 75 MHz): 11.43, 11.80, 12.78, 21.67, 21.98, 28.33, 29.96, 36.74, 37.17, 51.83, 51.92, 52.71, 52.75, 54.82, 90.11, 92.77, 98.25, 100.42, 118.77, 120.87, 128.52, 129.72, 130.77, 131.10, 133.28, 133.32, 134.14, 136.51, 136.90, 138.25, 138.52, 139.75, 149.31, 149.39, 151.03, 151.27, 153.03, 162.31, 166.19, 170.21, 173.50, 173.92. The atoms were numbered according to Scheme 1.

##### Chlorin **4A**

Dark blue solid, 7 mg, 19% yield. The purity observed by HPLC is >99%. MS (ESI) *m*/*z* 885 [C_54_H_52_N_4_O_8_+H]^+^. IR (ATR, cm^–1^): ν–NH 3341, ν_as_–CH_3_ 2953, ν_s_–CH_3_ 2850, ν–C=O (ester) 1718. UV–vis (DMSO, λ_max_ nm) (log ε): λ_1_ (Soret) 406 (5.01), λ_2_ (Q_IV_) 502 (3.97), λ_3_ (Q_III_) 536 (3.87), λ_4_ (Q_II_) 608 (3.60), λ_5_ (Q_I_) 666 (4.42). ^1^H NMR (CDCl_3_, 300 MHz): −2.58 (s, 2H, NH); 2.11 (s, 3H, H-18); 3.24 (2 t, superimposed, 4H, H-22 and H-25); 3.37 (s, 3H, H-20); 3.48 (s, 3H, H-19); 3.59 (s, 3H, H-17); 3.67 (dd, 1H, *J* = 1.76, H-28); 3.91 (s, 3H, H-31); 4.00 (dd, 1H, *J* = 6.76, H-28); 4.01 (s, 3H, H-32); 4.20 (t, 2H, ^3^*J* = 7.52, H-21); 4.35 (t, 2H, ^3^*J* = 7.61, H-24); 5.04 (s, 4H, Ph–CH_2_); 6.17 (dd, 1H, *J*_AB_ = 1.15, *J*_AX_ = 11.60, H_A_); 6.37 (dd, 1H, *J*_BA_ = 1.46, *J*_BX_ = 17.87, H_B_); 7.06 (m, 10H; Ph-H); 7.41 (dd; 1H, *J* = 1.75, *J* = 6.42, H-27); 8.18 (dd, 1H; *J*_XA_ = 11.52, *J*_XB_ = 17.73, H_X_); 9.09 (s, 1H; H- δ); 9.39 (s, 1H, H-α); 9.77 (s, 1H, H-γ); 9.82 (s, 1H, H-β). ^13^C NMR (CDCl_3_, 75 MHz): 11.45, 11.81, 12.79, 21.65, 21.97; 28.32, 29.97, 36.88, 37.30, 52.72, 52.76, 54.82, 66.43; 66.55, 90.15, 92.74, 98.42, 100.47, 118.73, 120.86, 124.95, 128.05, 128.53, 129.75, 130.74, 131.18, 133.25, 133.31, 134.19, 136.23, 136.88, 138.25, 138.62, 139.74, 149.34, 149.41, 151.05, 151.36, 153.00, 162.28, 166.21, 170.23, 172.94, 173.35. The atoms were numbered according to Scheme 1.

##### Chlorin **4B**

Brown solid, 17 mg, 44% yield. The purity observed by HPLC is >99%. MS (ESI) *m*/*z* 885 [C_54_H_52_N_4_O_8_ + H]^+^. IR (ATR, cm^–1^): ν–NH 3341, ν_as_–CH_3_ 2923, ν_s_–CH_3_ 2852, ν–C=O (ester) 1719. UV–vis (DMSO, λ_max_ nm) (log ε): λ_1_ (Soret) 406 (4.99), λ_2_ (Q_IV_) 502 (4.10), λ_3_ (Q_III_) 534 (3.94), λ_4_ (Q_II_) 608 (3.60), λ_5_ (Q_I_) 666 (4.59). ^1^H NMR (CDCl_3_, 300 MHz): −2.55 (s, 2H, NH); 2.04 (s, 3H, H-18); 3.15 (2 t, superimposed, 4H, H-22 and H-25); 3.31 (s, 3H, H-20); 3.37 (s, 3H, H-19); 3.55 (dd, 1H, *J* = 1.97, H-28); 3.59 (s, 3H, H-17); 3.82 (s, 3H, H-31); 3.90 (dd, 1H, *J* = 6.66, *J* = 22.15, H-28); 3.94 (s, 3H, H-32); 4.11 (t, 2H, ^3^*J* = 7.52, H-21); 4.24 (t, 2H, ^3^*J* = 7.61, H-24); 4.94 (s, 2H, Ph–CH_2_); 4.97 (s, 2H, Ph–CH_2_); 6.10 (dd, 1H, *J*_AB_ = 1.43, *J*_AX_ = 11.54, H_A_); 6.26 (dd, 1H, *J*_*BA*_ = 1.43, *J*_BX_ = 17.88, H_B_); 6.95–7.07 (m, 10H, Ph-H); 7.31 (dd; 1H, *J* = 2.01, *J* = 6.68, H-27); 8.11 (dd, 1H; *J*_XA_ = 11.57, *J*_XB_ = 17.84, H_X_); 9.15 (s, 1H, H-α); 9.21 (s, 1H, H-β); 9.60 (s, 1H, H-γ); 9.71 (s, 1H, H-δ). ^13^C NMR (CDCl_3_, 75 MHz): 11.45, 11.81, 12.79, 21.65, 21.97, 28.32, 29.97, 36.88, 37.30, 52.72, 52.76, 54.82, 66.43, 66.55, 90.15, 92.74, 98.42, 100.47, 118.73, 120.86, 124.95, 128.05, 128.53, 129.75, 130.74, 131.18, 133.25, 133.31, 134.19, 136.23, 136.88, 138.25, 138.62, 139.74, 149.34, 149.41, 151.05, 151.36, 153.00, 162.28, 166.21, 170.23, 172.94, 173.35. The atoms were numbered according to Scheme 1.

#### Synthesis of the Complexes

##### General Procedure

The ruthenium complexes were prepared by adapting a reported procedure.^41^ A solution of 1.0 mmol of the corresponding ligand (**1**, **2**, **3A**, and **3B**) and 1.1 mmol of [Ru_3_(CO)_12_] in 10 mL of benzene was stirred at reflux temperature for 24 h in the dark. The solvent was evaporated to dryness under vacuum, and the crude solid was purified through column chromatography on neutral alumina using a 1:2 hexane/acetone mixture as eluent.

**Ru-1**: A pink solid was obtained from 30.0 mg (0.051 mmol) of **1** and 36.0 mg (0.056 mmol) of [Ru_3_(CO)_12_] in 78% yield (28 mg). The purity observed by HPLC is >99%. MS (FAB^+^) *m*/*z* 718 [C_37_H_36_N_4_O_5_Ru + H]^+^. IR (ATR, cm^–1^): ν_as_–CH_3_ 2921, ν_s_–CH_3_ 2858, ν–Ru–C=O 1914, ν–C=O (ester) 1744. UV–vis (DMSO, λmax nm) (log ε): λ_1_ (Soret) 404 (4.76), λ_2_ (Q_β_) 524 (4.08), λ_3_ (Q_α_) 558 (4.27). ^1^H NMR (CD_3_CN, 500 MHz): 3.30 (t, 4H, H-22 and H-25); 3.57, 3.59, 3.60, 3.61, 3.70, and 3.71 (6s, 18H, H-17, H-18, H-19, H-20, O–CH_3_ and O–CH_3_); 4.33 (t, 4H, H-21 and H-24); 6.11 (dd, 2H, *J*_AB_ = 1.52, *J*_AX_ = 11.51, H_A_); 6.40 (dd, 2H, *J*_BA_ = 1.49, *J*_BX_ = 17.82, H_B_); 8.38 (m, 2H, *J*_XA_ = 11.47, *J*_XB_ = 17.85, H_X_); 9.94, 10.06, 10.10, 10.17 (4s, 4H, H-α, H-β, H-γ and H-δ). ^13^C NMR (CD_3_CN, 125 MHz): 11.72, 12.94, 14.32, 22.38, 23.29, 30.83, 32.26, 37.56, 52.06, 99.34, 100.01, 100.21, 100.67, 119.87, 174.46, 207.46 (Ru-CO). The atoms were numbered according to Scheme 2.

**Ru-2**: A pink solid was obtained from 30.0 mg (0.041 mmol) of **2** and 28.0 mg (0.044 mmol) of [Ru_3_(CO)_12_] in 61% yield (21 mg). The purity observed by HPLC is >99%. MS (ESI) *m*/*z* (%) 872 [C_37_H_36_N_4_O_5_Ru + 2H]^+^. IR (ATR, cm^–1^): ν_as_–CH_3_ 2922, ν_s_–CH_3_ 2859, ν–Ru–C=O 1915, ν–C=O (ester) 1729. UV–vis (DMSO, λmax nm) (log ε): λ_1_ (Soret) 402 (4.76), λ_2_ (Q_β_) 524 (4.08), λ_3_ (Q_α_) 558 (4.11). ^1^H NMR (CD_3_CN, 500 MHz): 3.36 (t, 4H, H-22 and H-25); 3.52, 3.55, 3.70, and 3.71 (4s, 12H, H-17, H-18, H-19 and H-20); 4.34 (t, 4H, H-21 and H-24); 5.05 (s, 4H, Ph–CH_2_); 6.11 (dd, 2H, *J*_AB_ = 1.72, *J*_AX_ = 11.54, H_A_); 6.40 (dd, 2H, *J*_BA_ = 1.78, *J*_BX_ = 17.89, H_B_); 7.13 (m, 10H, Ph-H); 8.40 (dd, 2H, *J*_XA_ = 11.75, *J*_XB_ = 17.58, H_X_); 9.98, 10.04, 10.08, and 10.17 (4s, 4H, H-α, H-β, H-γ and H-δ). ^13^C NMR (CD_3_CN, 125 MHz): 11.58, 11.79, 18.07, 20.26, 22.52, 29.70, 37.87, 37.93, 66.78, 99.18, 99.36, 99.39, 99.52, 128.58, 128.70, 128.79, 129.29, 129.32, 131.32, 131.42, 136.81, 136.91, 137.47, 138.04, 138.06, 140.02, 140.05, 142.01, 142.34, 142.57, 143.02, 143.05, 143.31, 143.36, 143.91, 144.02, 174.01, 211.04 (Ru-CO). The atoms were numbered according to Scheme 2.

**Ru-3A**: A green solid was obtained from 30.0 mg (0.041 mmol) of **3A** and 29.0 mg (0.045 mmol) of [Ru_3_(CO)_12_] in 13% yield (4 mg). The purity observed by HPLC is >98%. MS (ESI) *m*/*z* (%) 862 [C_43_H_42_N_4_O_9_Ru + 2H]^+^. IR (ATR, cm^–1^): ν_as_–CH_3_ 2992, ν_s_–CH_3_ 2854, ν–Ru–C=O 1927, ν–C=O (ester) 1722. UV–vis (DMSO, λmax nm) (log ε): λ_1_ (Soret) 406 (4.83), λ_2_ (Q_β_) 536 (3.81), λ_3_ (Q_α_) 568 (3.94), λ_4_ (Q_γ_) 606 (4.24). ^1^H RMN (acetone-*d*_6_, 300 MHz): 2.83 (s, 3H, H-18); 3.14 (t, 2H, H-22); 3.26 (t, 2H, H-25); 3.31 (s, 3H, H-19); 3.55 (s, 3H, H-20); 3.56 (s, 3H, H-17) 3.57 (dd, 1H, *J* = 2.39, H-28); 3.61 (s, 3H, O–CH_3_); 3.62 (s, 3H, O–CH_3_); 3.66 (dd, 1H, *J* = 7.59, H-28); 3.90 (s, 3H, H-31); 4.08 (s, 3H, H-32); 4.11 (t, 2H, H-24); 4.37 (t, 2H, H-21); 6.14 (dd, 1H, *J*_AB_ = 1.58, *J*_AX_ = 11.62, H_A_); 6.37 (dd, 1H, *J*_BA_ = 1.63, *J*_BX_ = 17.91, H_B_), 7.65 (dd, 1H, *J* = 2.30, H-27), 8.23 (dd, 1H, *J*_XA_ = 11.67, *J*_XB_ = 17.88, H_X_), 9.20 (s, 1H, H- δ), 9.55 (s, 1H, H-α), 9.77 (s, 1H, H-γ), 9.91 (s, 1H, H-β). ^13^C NMR (acetone-*d*_6_, 75 MHz): 11.43, 11.50, 12.35, 21.97, 22.17, 37.04, 37.47, 51.69, 51.80, 52.00, 52.79, 91.49, 93.15, 99.95, 100.39, 120.57, 121.95, 127.14, 130.47, 137.91, 141.39, 143.33, 149.10, 149.49, 151.67, 152.00, 152.75, 163.53, 166.63, 173.68, 173.87. The atoms were numbered according to Scheme 2.

**Ru-3B**: A green solid was obtained from 30.0 mg (0.041 mmol) of **3B** and 29.0 mg (0.045 mmol) of [Ru_3_(CO)_12_] in 16% yield (6 mg). The purity observed by HPLC is >98%. MS (ESI) *m*/*z* (%) 862 [C_43_H_42_N_4_O_9_Ru + 2H]^+^. IR (ATR, cm^–1^): ν_as_–CH_3_ 2923, ν_s_–CH_3_ 2857, ν–Ru–C=O 1923, ν–C=O (ester) 1721. UV–vis (DMSO, λmax nm) (log ε): λ_1_ (Soret) 406 (4.63), λ_2_ (Q_β_) 534 (3.66), λ_3_ (Q_α_) 568 (3.83), λ_4_ (Q_γ_) 606 (4.02). ^1^H NMR (acetone-*d*_6_, 300 MHz): 2.79 (s, 3H, H-18); 3.17 (t, 2H, H-22); 3.25 (t, 2H, H-25); 3.42 (s, 3H, H-20); 3.54 (s, 3H, H-17); 3.60 (s, 3H, O–CH_3_); 3.61 (s, 3H, O–CH_3_); 3.68 (s, 3H, H-19); 3.76 (d, 1H, H-28) 3.88 (s, 3H, H-31); 4.00 (d, 1H, H-28); 4.03 (s, 3H, H-32); 4.16 (t, 2H); 4.34 (t, 2H); 6.21 (dd, 1H, *J*_AB_ = 1.447, *J*_AX_ = 11.586, H_A_); 6.39 (dd, 1H, *J*_BA_ = 1.466, *J*_BX_ = 17.892, H_B_); 7.68 (dd; 1H, ^3^*J* = 6.775, H-27); 8.21 (dd, 1H; *J*_XA_ = 11.603, *J*_XB_ = 17.852, H_X_); 9.31 (s, 1H, H-α); 9.55 (s, 1H, H-β); 9.87 (s, 1H, H-γ); 9.95 (s, 1H, H-δ). ^13^C NMR (acetone-*d*_6_, 75 MHz): 11.15, 11.49, 12.52, 21.84, 22.14, 36.92, 37.42, 51.69, 51.78, 52.79, 52.92, 55.07, 90.98, 93.37, 99.35, 101.34, 120.89, 121.36, 130.02, 130.36, 137.01, 137.92, 139.05, 139.39, 149.55, 151.84, 152.45, 154.25, 163.23, 166.61, 170.32, 173.63, 173.88. The atoms were numbered according to Scheme 2.

#### Crystallography

Single crystals suitable for X-ray diffraction were obtained for **3B** and **Ru-1** by slow evaporation of dichloromethane/hexane solution. Data were collected at room temperature (298 K) on a Bruker Apex-II CCD diffractometer using monochromatic graphite Mo Kα (0.71073Ǻ) radiation. Cell determination and final cell parameters were obtained on all reflections using the Bruker SAINT software included in the APEX2 software.^87,88^ The integration and scaling of the data were carried out using the Bruker SAINT software.^87,88^ For compound **3B**, weak high-angle diffractions, combined with the multiple contents and some degree of disorder, caused a large number of refined parameters with poor data/parameters ratio, which led to the observed low C–C precision. However, the identity of the structure was clearly established without issue. The CIF files have been deposited in the Cambridge Structural Data Base under CCDC 2400652 for **3B** and 2400653 for **Ru-1**. Copies of the data can be obtained, free of charge, at www.ccdc.cam.ac.uk.

#### Stability Studies

Stability studies were performed using a Shimadzu 2600 UV–vis spectrophotometer at 37 °C from 1 × 10^–5^ M solutions of each compound in pure DMSO or in PBS (Dulbecco’s Phosphate Buffered Saline, Dominique Dutscher SAS without magnesium and calcium) with 0.1% DMSO. Spectra were recorded every hour for 24 h in DMSO and every hour for 8 h in PBS/DMSO (0.1%).

#### Photostability Studies

The photodegradation of the new compounds was evaluated as described in the literature.^46^ Solutions of each compound at a concentration of 1 × 10^–5^ M were prepared in pure DMSO or in PBS/DMSO (0.1%). Each solution was irradiated at 37 °C with white light (light temperature: 8500 K and light intensity: 50%, SmallRing brand) at different times until completing 15 min (irradiation time: 0, 3, 6, 9, 12, and 15 min). The absorption spectrum was recorded after each irradiation.

#### EPR Studies

The detection of singlet oxygen is based on the specific reaction between ^1^O_2_ and 2,2,6,6-tetramethylpiperidine (TEMP), which produces a stable (2,2,6,6-tetramethylpiperidin-1-yl)oxyl (TEMPO) radical adduct. Detection of ^1^O_2_ was carried out according to the following procedure. Solutions of 1 × 10^–3^ M of the compounds and 0.06 M TEMP in ethanol/DMSO (1%, final volume 400 μL) equilibrated with air at room temperature were irradiated with visible light in a flat cell for up to 15 min, generating the characteristic triplet signal corresponding to TEMPO. EPR spectra were obtained with the following parameters: center field, 334.5 ± 4 mT; power, 1.0 mW; microwave frequency, 9.43 GHz; modulation width, 0.5 mT; time constant, 0.1 s; amplitude, 10. The oxygen concentration was measured using an Iuzmar dissolved oxygen meter. For the detection of superoxide radical (O_2_^•–^), 5,5-dimethyl-1-pyrroline-*N*-oxide (DMPO, from Dojindo Japan) was used. Detection of O_2_^•–^ was carried out according to the following procedure. A mixture of 1 × 10^–3^ M solution of the compounds and a 0.07 M solution of DMPO in methanol/DMSO (1%, final volume 400 μL) at room temperature were irradiated with visible light in a flat cell for up to 15 min, generating the signal of the DMPO-O_2_^•–^ adduct. EPR spectra were obtained with the following parameters: center field, 335 ± 4 mT; power, 8.0 mW; microwave frequency, 9.43 GHz; modulation width, 0.63 mT; time constant, 0.1 s; amplitude, 100.

#### Fluorescence Quantum Yield Measurements

The fluorescence quantum yields were determined using verteporfin in DMSO as a reference (Φ_f_ = 0.0085).^46,89^ The free ligands were excited at 503 nm, and the ruthenium complexes at 573 nm. The quantum yield was calculated following eq 1.^90,91^
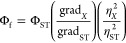
1Where ST and *X* are standard and analyte, respectively, Φ_f_ is the fluorescence quantum yield, Grad is the gradient from the area of integration fluorescence intensity *vs* absorbance, and η_*X*_ and η_ST_ are the refractive index of the solvent used to measure the fluorescence spectrum of the analyte and the standard, respectively.

#### Oxygen Singlet Quantum Yield Measurements

Quantum yields of singlet oxygen (^1^O_2_) production were determined in DMSO equilibrated with oxygen and white light, using verteporfin as a reference and 1,3-diphenylisobenzofuran (DPBF) as a chemical trap which in the presence of singlet oxygen is oxidized to 1,2-dibenzoylbenzene.^92^ The absorption band of DPBF was monitored at 214 nm.^93^ The ^1^O_2_ production quantum yields of the compounds were determined following eq 2.^94,95^
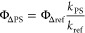
2Where PS is the photosensitizer, Φ_Δref_ is singlet oxygen production quantum yield of verteporfin (Φ_Δ_ = 0.77 in DMSO^46^) and *k*_ref_ and *k*_PS_ are the decay constant of DPBF in the presence of the standard (VP) or each PS and are obtained from the slope obtained from the absorbance of DPBF at different irradiation times.

#### Lipophilicity Studies

The partition coefficient (log *P*_o/PBS_) of each compound was determined experimentally by the “*shake-flask*” method at room temperature following reported methods.^57,96^ A mixture of 1-octanol and PBS in equal amounts was shaken for 24 h, after which the phases were allowed to separate for 24 h. Then, a 1 × 10^–5^ M solution of each compound is prepared in 1-octanol/PBS (1:1), and the mixture is left to stir for 24 h. After 24 h of stirring, the mixture is left for 24 h to separate the two phases. Finally, the concentration of each compound in the two phases is determined by UV–vis spectroscopy. log*P* was determined by the following eq 3.^97^
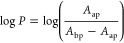
3Where *A*_ap_ is the absorbance after partition, and *A*_bp_ is the absorbance before partition.

### Biology

#### Cell Lines

The human gastric adenocarcinoma (AGS) cell line was acquired from ATCC (Manassa, VA). AGS cells were cultured in RPMI 1640 medium (Roswell Park Memorial Institute) containing 10% fetal bovine serum (FBS) at 37 °C in a humidified atmosphere of 95% air and 5% CO_2_.

#### MTT Survival Assays

The antiproliferative activity of cancer cells was determined by the 3-(4,5-dimethylthiazol-2-yl)-2,5-diphenyltetrazolium bromide (MTT) assay. Cells were seeded at 10.000 cells per well (200 μL) in Cellstar 96-well plates (Greiner Bio-One) and further incubated for 24 h. Cells were treated with each compound at different concentrations for 8 h in the dark. After this time, the medium was removed, fresh medium was added, and cells were kept in the dark or irradiated with white light for 15 min. Subsequently, the plates were incubated in the dark for an additional 48 h, and the MTT assay was performed as previously described in the literature.^34^ Measurements were performed at 550 nm with the Tristar2Multimode Reader (Berthold Technologies).

#### Western Blotting^98^

AGS cells were treated with the IC_50_ of each compound for 4 h at 37 °C in the dark and then were either kept in the dark or irradiated with white light for 15 min and further cultivated in the dark for an additional 24 h. Cells were then lysed in lysis buffer (50 mM Tris–HCl pH 6.7, NaCl 150 mM, NP40 1%). Total protein concentration was determined using Bradford assay and adjusted to the same quantity (50 μg) for each experiment. A total of 30 μg of proteins were resolved by 6–15% SDS-PAGE (depending on protein molecular weight) and transferred to nitrocellulose blotting membranes according to standard methods. Membranes were blocked with blocking buffer (containing 5% milk powder or 1% BSA) for 1 h at room temperature, then washed three times with TBST or PBST (containing 0.1% Tween-20) and incubated with primary antibodies at 4 °C overnight. The following day, the membranes were washed thrice with PBST or PBST. The membranes were incubated with antirabbit or antimouse antibodies for 1 h at room temperature and visualized by enhanced chemiluminescence using the ClarityTM ECL Western Blotting Substrate Bio-Rad reagent according to the manufacturer’s instructions. Signals were acquired with the help of Immobilon Crescendo or Ozyme on Syngene PX*i* equipment using GeneSys software. Protein bands were quantified using ImageJ software.^99^ Western blots were performed using the following antibodies: GAPDH (6C5) (1:2000, sc-32233, Santa Cruz Biotechnology); Cleaved PARP (D64E10) (1:1000, 5625S, Cell Signaling Technology); COX2 (D5H5) (1:1000, 12282S, Cell Signaling Technology); CHOP/GADD 153 (B-3) (1:500, sc-7351, Santa Cruz Biotechnology); Cleaved Caspase-3 (D175) (1:1000, 9661L, Cell Signaling Technology); GPX4 (1:1000, ab25066, abcan) and LC3B (D11) (1:1000, 3868S, Cell Signaling Technology). The secondary antibodies were: Antirabbit IgG (1:10000, 7074S, Cell Signaling Technology) and Anti-Mouse IgG (1:8000, 7076S, Cell Signaling Technology). Loading was controlled by glyceraldehyde-3-phosphate dehydrogenase (GAPDH) for normalization.

## Supporting Information Available

Mass spectra; IR spectra; NMR spectra; UV–vis spectra; EPR spectra and cell survival graphs (Figures S1–S102); crystallographic data and quantum yields (Tables S1 and S2) (PDF)
