# Supplementary material for: Protoporphyrin IX-Derived Ruthenium(II) Complexes for Photodynamic Therapy in Gastric Cancer Cells
Source: Inorg Chem. 2025 May 2;64(19):9684–702. doi: 10.1021/acs.inorgchem.5c00896 (PMC12093383; doi:10.1021/acs.inorgchem.5c00896)
Supplement: Supplementary file 1 — ic5c00896_si_001.pdf [file ic5c00896_si_001.pdf]

Protoporphyrin IX – derived ruthenium(II) complexes for photodynamic therapy in gastric cancer cells

Andres Restrepo-Acevedo,[a] Maria Isabel Murillo,[a] Christophe Orvain,[b,c] Chloé Thibaudeau,[b] Sevda Recberlik,[c] Lucas Verget,[a,d] Virginia Gómez Vidales,[a] Christian Gaiddon,[b,e] Georg Mellitzer,[b,c]* Ronan Le Lagadec[a]*

[a]Instituto de Química UNAM, Circuito Exterior s/n Ciudad Universitaria, 04510 Ciudad de México, Mexico

[b]Strasbourg Université Inserm UMR_S U1113, IRFAC, 3 Avenue Molière, 67200 Strasbourg, France

[c]INSERM UMR_S1260, Regenerative Nanomedicine Team GP-SMIT, CRBS, 1 Rue Eugène Boeckel, 67085 Strasbourg, France

[d]Faculté de chimie, Sorbonne Université, 4 place Jussieu 75005 Paris, France

[e]UMR7242, Biotechnology et Signalisation Cellulaire, 300 Bld S. Brant, FR-67412 Illkirch Cedex, France

Corresponding Authors: Georg Mellitzer (mellitzer@unistra.fr); Ronan Le Lagadec (ronan@unam.mx)

Figures

Figure S1. FT-IR spectrum of **1**

Figure S2. FAB-MASAS spectrum of **1** (positive detection mode)

Figure S3. ^{1}H-NMR spectrum of **1** in $CDCl_3$, 300 MHz

Figure S4. ^{13}C-NMR spectrum of **1** in $CDCl_3$, 300 MHz

Figure S5. DEP-135 spectrum of **1** in $CDCl_3$, 300 MHz

Figure S6. HSQC spectrum of **1** in $CDCl_3$, 300 MHz

Figure S7. Analytic HPLC of **1** with detection at 406 nm

Figure S8. FT-IR spectrum of **2**

Figure S9. FAB-MASAS spectrum of **2** (positive detection mode)

Figure S10. ^{1}H-NMR spectrum of **2** in $CDCl_3$, 300 MHz

Figure S11. ^{13}C-NMR spectrum of **2** in $CDCl_3$, 300 MHz

Figure S12. DEP-135 spectrum of **2** in $CDCl_3$, 300 MHz

Figure S13. HSQC spectrum of **2** in $CDCl_3$, 300 MHz

Figure S14. Analytic HPLC of **2** with detection at 408 nm

Figure S15. FT-IR spectrum of **3A**

Figure S16. ESI-HRMS spectrum of **3A** (positive detection mode)

Figure S17. ^{1}H-NMR spectrum of **3A** in $CDCl_3$, 300 MHz

Figure S18. ^{13}C-NMR spectrum of **3A** in $CDCl_3$, 300 MHz

Figure S19. DEP-135 spectrum of **3A** in $CDCl_3$, 300 MHz

Figure S20. NOESY spectrum of **3A** in $CDCl_3$, 300 MHz

Figure S21. Analytic HPLC of **3A** with detection at 406 nm

Figure S22. FT-IR spectrum of **3B**

Figure S23. ESI-HRMS spectrum of **3B** (positive detection mode)

Figure S24. ^{1}H-NMR spectrum of **3B** in $CDCl_3$, 300 MHz

Figure S25. ^{13}C-NMR spectrum of **3B** in $CDCl_3$, 300 MHz

Figure S26. DEP-135 spectrum of **3B** in $CDCl_3$, 300 MHz

Figure S27. NOESY spectrum of **3B** in $CDCl_3$, 300 MHz

Figure S28. Analytic HPLC of **3B** with detection at 404 nm

Figure S29. FT-IR spectrum of **4A**

Figure S30. ESI-HRMS spectrum of **4A** (positive detection mode)

Figure S31. ^{1}H-NMR spectrum of **4A** in $CDCl_3$, 300 MHz

Figure S32. ^{13}C-NMR spectrum of **4A** in $CDCl_3$, 300 MHz

Figure S33. DEP-135 spectrum of **4A** in $CDCl_3$, 300 MHz

Figure S34. NOESY spectrum of **4A** in $CDCl_3$, 300 MHz

Figure S35. Analytic HPLC of **4A** with detection at 406 nm

Figure S36. FT-IR spectrum of **4B**

Figure S37. ESI-HRMS spectrum of **4B** (positive detection mode)

Figure S38. ^{1}H-NMR spectrum of **4B** in $CDCl_3$, 300 MHz

Figure S39. ^{13}C-NMR spectrum of **4B** in $CDCl_3$, 300 MHz

Figure S40. NOESY spectrum of **4B** in $CDCl_3$, 300 MHz

Figure S41. Analytic HPLC of **4B** with detection at 406 nm

Figure S42. FT-IR spectrum of **Ru-1**

Figure S43. FAB-MASAS spectrum of **Ru-1** (positive detection mode)

Figure S44. ^{1}H-NMR spectrum of **Ru-1** in CD_3CN, 500 MHz

Figure S45. ^{13}C-NMR spectrum of **Ru-1** in CD_3CN, 500 MHz

Figure S46. Analytic HPLC of **Ru-1** with detection at 404 nm

Figure S47. FT-IR spectrum of **Ru-2**

Figure S48. ESI-HRMS spectrum of **Ru-2** (positive detection mode)

Figure S49. ^{1}H-NMR spectrum of **Ru-2** in CD_3CN, 500 MHz

Figure S50. ^{13}C-NMR spectrum of **Ru-2** in CD_3CN, 500 MHz

Figure S51. Analytic HPLC of **Ru-2** with detection at 402 nm

Figure S52. FT-IR spectrum of **Ru-3A**

Figure S53. ESI-HRMS spectrum of **Ru-3A** (positive detection mode)

Figure S54. ^{1}H-NMR spectrum of **Ru-3A** in acetone-d_6, 300 MHz

Figure S55. ^{13}C-NMR spectrum of **Ru-3A** in acetone-d_6, 300 MHz

Figure S56. Analytic HPLC of **Ru-3A** with detection at 406 nm

Figure S57. FT-IR spectrum of **Ru-3B**

Figure S58. ESI-HRMS spectrum of **Ru-3B** (positive detection mode)

Figure S59. ^{1}H-NMR spectrum of **Ru-3B** in acetone-d_6, 300 MHz

Figure S60. ^{13}C-NMR spectrum of **Ru-3B** in acetone-d_6, 300 MHz

Figure S61. Analytic HPLC of **Ru-3B** with detection at 406 nm

Figure S62. Stability test of **1** in DMSO at 37 °C and 1×10^{-5} M

Figure S63. Stability test of **2** in DMSO at 37 °C and 1×10^{-5} M

Figure S64. Stability test of **Ru-2** in DMSO at 37 °C and 1×10^{-5} M

Figure S65. Stability test of **3A** in DMSO at 37 °C and 1×10^{-5} M

Figure S66. Stability test of **Ru-3A** in DMSO at 37 °C and 1×10^{-5} M

Figure S67. Stability test of **3B** in DMSO at 37 °C and 1×10^{-5} M

Figure S68. Stability test of **Ru-3B** in DMSO at 37 °C and 1×10^{-5} M

Figure S69. Stability test of **4A** in DMSO at 37 °C and 1×10^{-5} M

Figure S70. Stability test of **4B** in DMSO at 37 °C and 1×10^{-5} M

Figure S71. Stability test of **1** in PBS/DMSO(0.1%) at 37 °C and 1×10^{-5} M

Figure S72. Stability test of **2** in PBS/DMSO(0.1%) at 37 °C and 1×10^{-5} M

Figure S73. Stability test of **Ru-2** in PBS/DMSO(0.1%) at 37 °C and 1×10^{-5} M

Figure S74. Stability test of **3A** in PBS/DMSO(0.1%) at 37 °C and 1×10^{-5} M

Figure S75. Stability test of **Ru-3A** in PBS/DMSO(0.1%) at 37 °C and 1×10^{-5} M

Figure S76. Stability test of **3B** in PBS/DMSO(0.1%) at 37 °C and 1×10^{-5} M

Figure S77. Stability test of **Ru-3B** in PBS/DMSO(0.1%) at 37 °C and 1×10^{-5} M

Figure S78. Stability test of **4A** in PBS/DMSO(0.1%) at 37 °C and 1×10^{-5} M

Figure S79. Stability test of **4B** in PBS/DMSO(0.1%) at 37 °C and 1×10^{-5} M

Figure S80. Photodegradation of compound **1** in DMSO using white light

Figure S81. Photodegradation of compound **2** in DMSO using white light

Figure S82. Photodegradation of compound **Ru-2** in DMSO using white light

Figure S83. Photodegradation of compound **3A** in DMSO using white light

Figure S84. Photodegradation of compound **Ru-3A** in DMSO using white light

Figure S85. Photodegradation of compound **3B** in DMSO using white light

Figure S86. Photodegradation of compound **4A** in DMSO using white light

Figure S87. Photodegradation of compound **4B** in DMSO using white light

Figure S88. Absorption and emission spectra in DMSO for **1** excitation at 503 nm

Figure S89. Absorption and emission spectra in DMSO for **2** excitation at 503 nm

Figure S90. Absorption and emission spectra in DMSO for **3A** excitation at 503 nm

Figure S91. Absorption and emission spectra in DMSO for **3B** excitation at 503 nm

Figure S92. Absorption and emission spectra in DMSO for **4A** excitation at 503 nm

Figure S93. Absorption and emission spectra in DMSO for **4B** excitation at 503 nm

Figure S94. EPR spectra upon irradiation for 5 and 15 min of **1** in the presence of TEMP varying the oxygen concentration: (a) 5 % of oxygen, (b) 45 % of oxygen, (c) 96 % of oxygen.

Figure S95. EPR spectra upon irradiation for 5 and 15 min of **3B** in the presence of TEMP varying the oxygen concentration: (a) 5 % of oxygen, (b) 45 % of oxygen, (c) 96 % of oxygen.

Figure S96. EPR spectra upon irradiation for 5 and 15 min of **Ru-3B** in the presence of TEMP varying the oxygen concentration: (a) 5 % of oxygen, (b) 45 % of oxygen, (c) 96 % of oxygen.

Figure S97. Cell viability curves for the compounds **1** (left) and **Ru-1** (right) in the AGS cell line.

Figure S98. Cell viability curves for the compounds **2** (left) and **Ru-2** (right) in the AGS cell line.

Figure S99. Cell viability curves for the compounds **3A** (left) and **Ru-3A** (right) in the AGS cell line.

Figure S100. Cell viability curves for the compounds **3B** (left) and **Ru-3B** (right) in the AGS cell line.

Figure S101. Cell viability curves for the compounds **4A** (left) and **4B** (right) in the AGS cell line.

Figure S102. Relative LC3BI/II expression

Tables

Table S1. Crystallographic data for **Ru-1** and **3B**

Table S2. Spectroscopic properties and $^{1}O_2$ quantum yields in DMSO

Figure S1. FT-IR spectrum of **1**

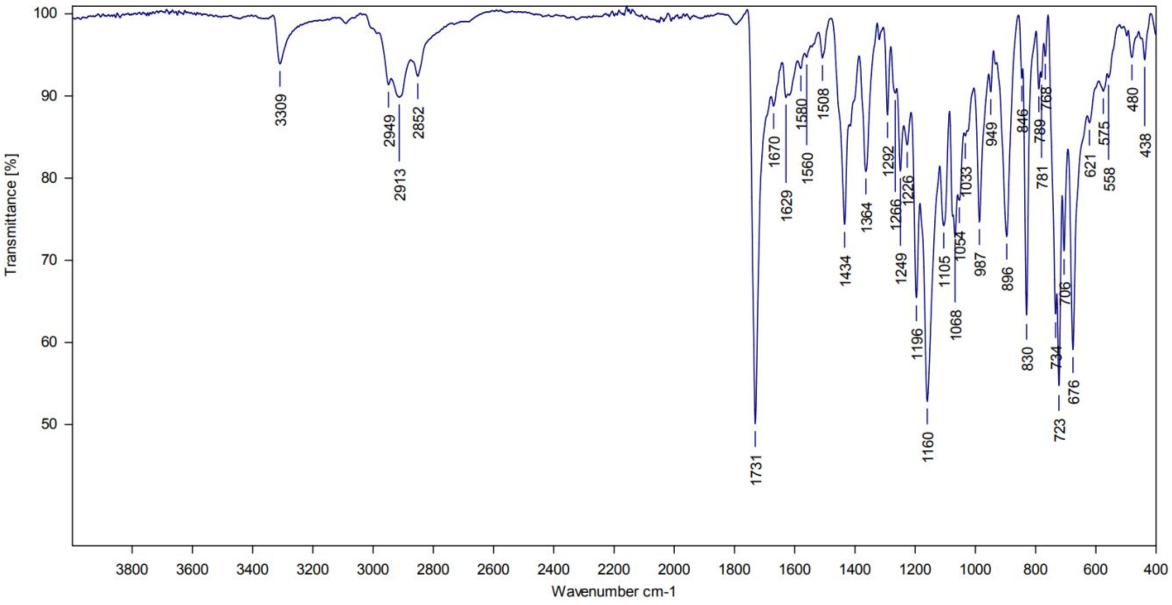

Figure S2. FAB-MASAS spectrum of **1** (positive detection mode)

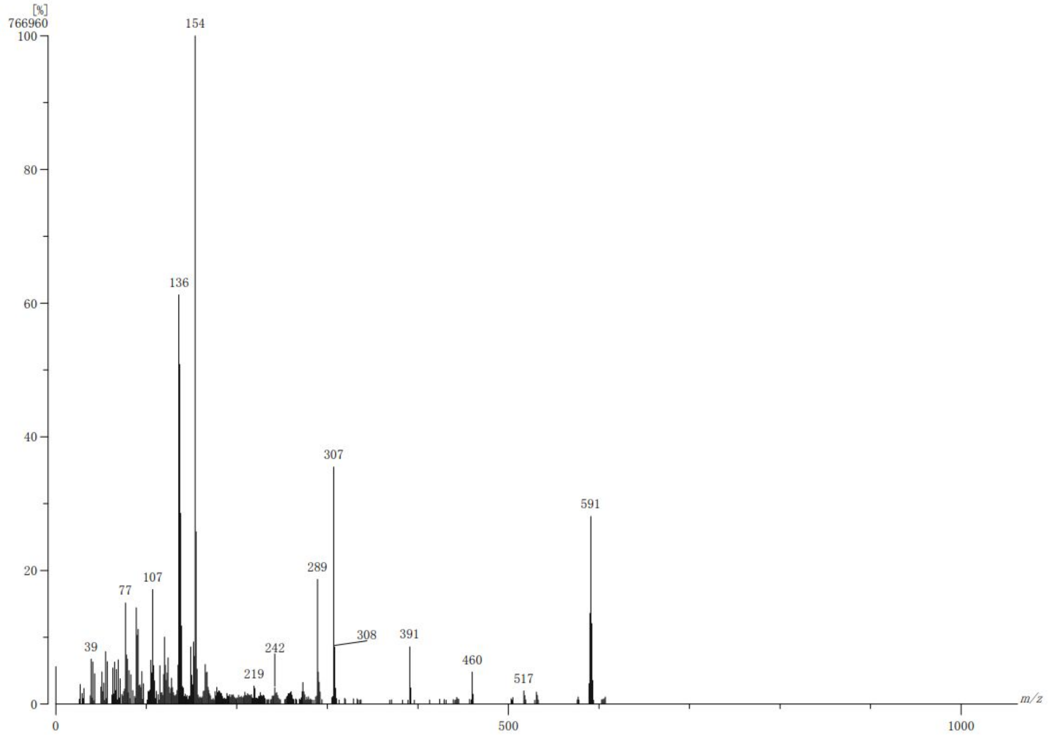

Figure S3. ^{1}H-NMR spectrum of **1** in $CDCl_3$, 300 MHz

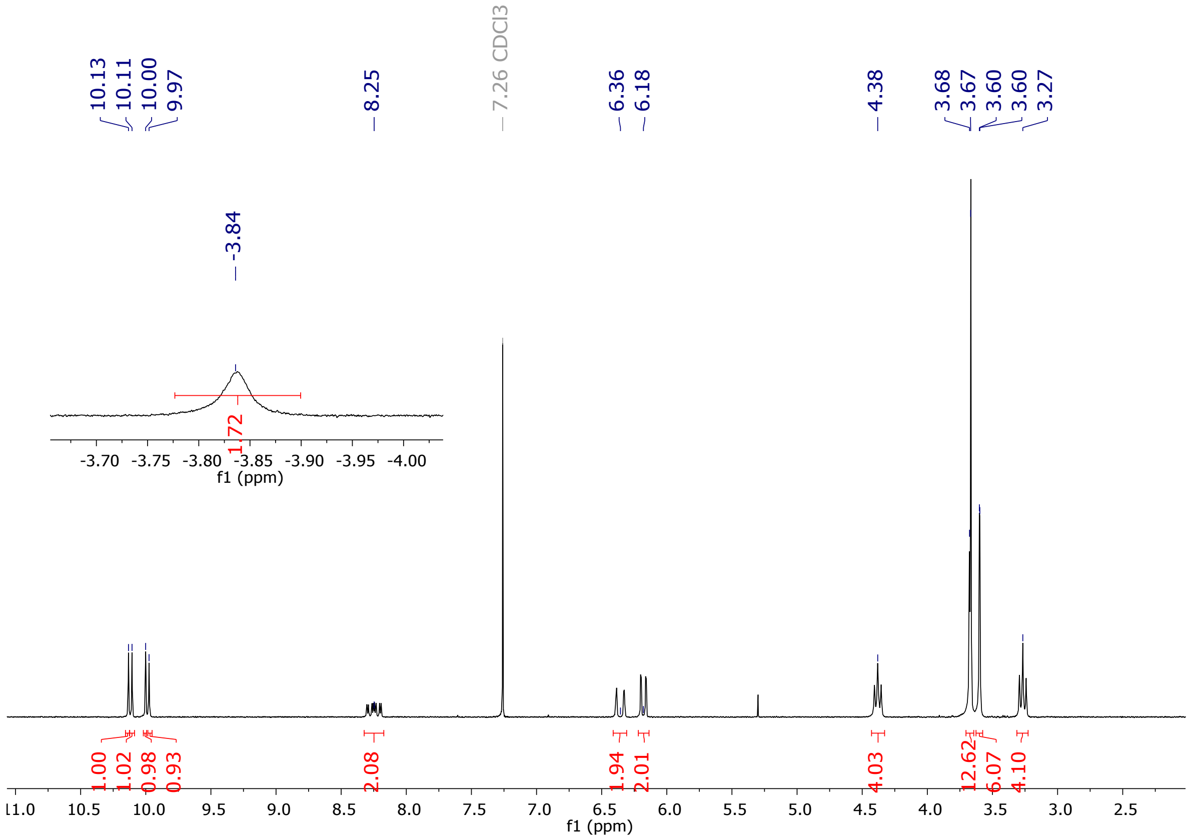

Figure S4. ^{13}C-NMR spectrum of **1** in $CDCl_3$, 300 MHz

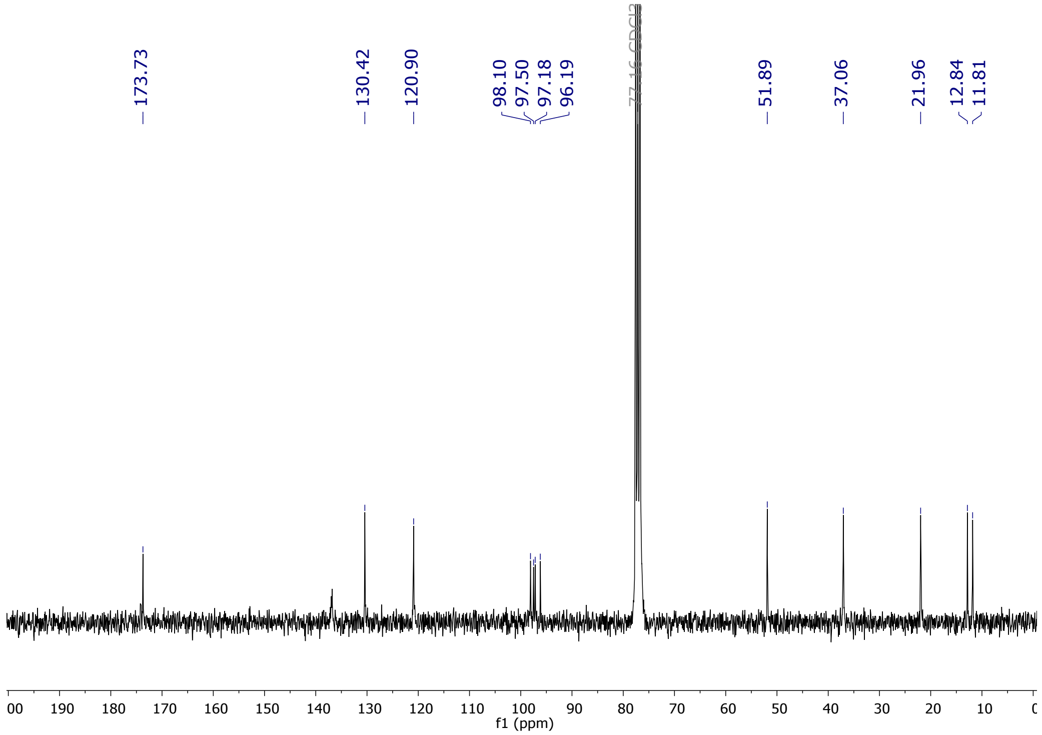

Figure S5. DEP-135 spectrum of **1** in $CDCl_3$, 300 MHz

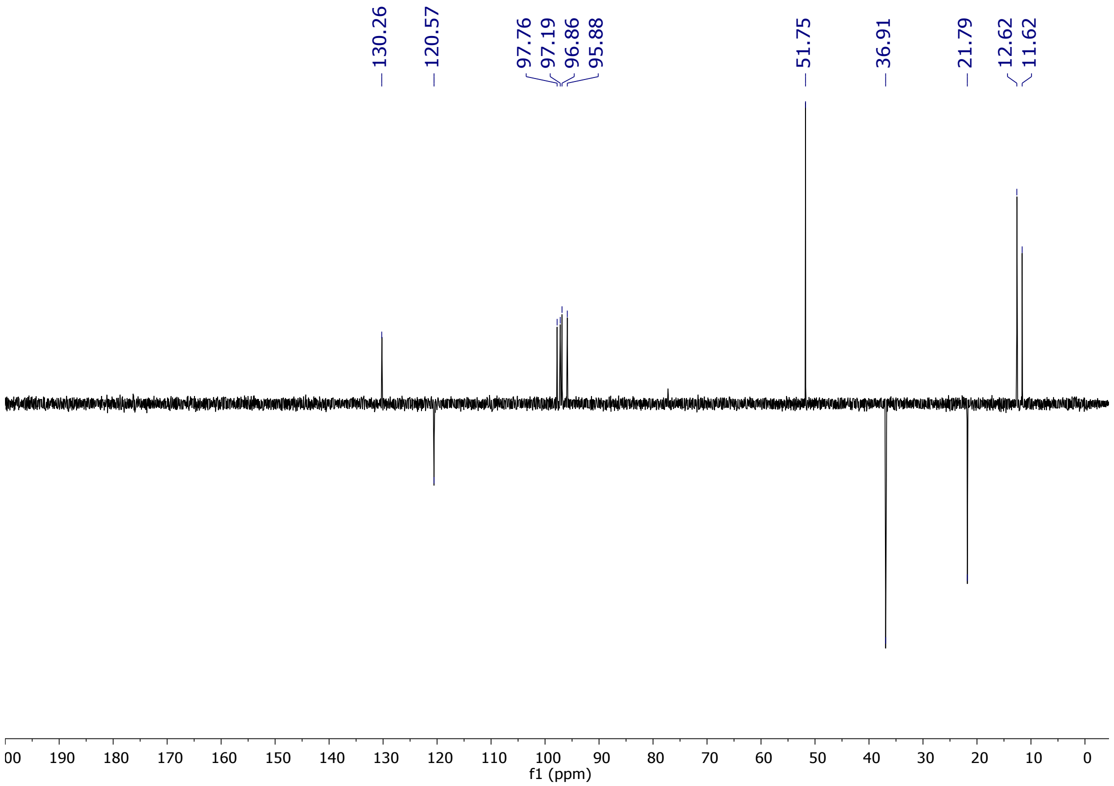

Figure S6. HSQC spectrum of **1** in $CDCl_3$, 300 MHz

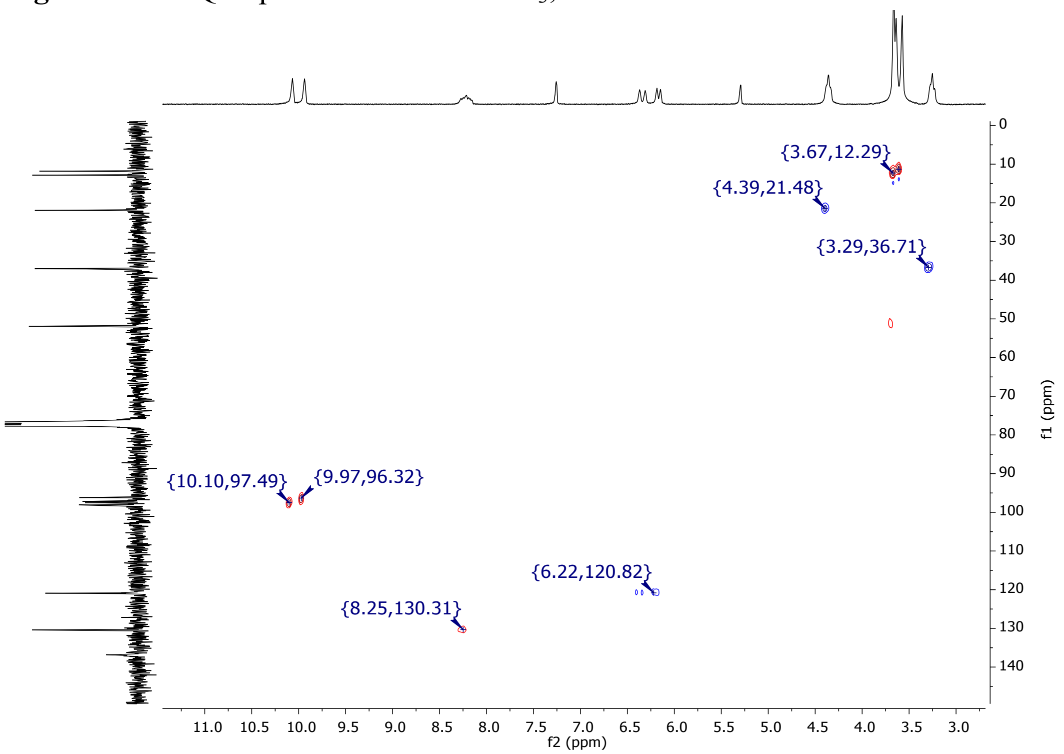

Figure S7. Analytic HPLC of **1** with detection at 406 nm

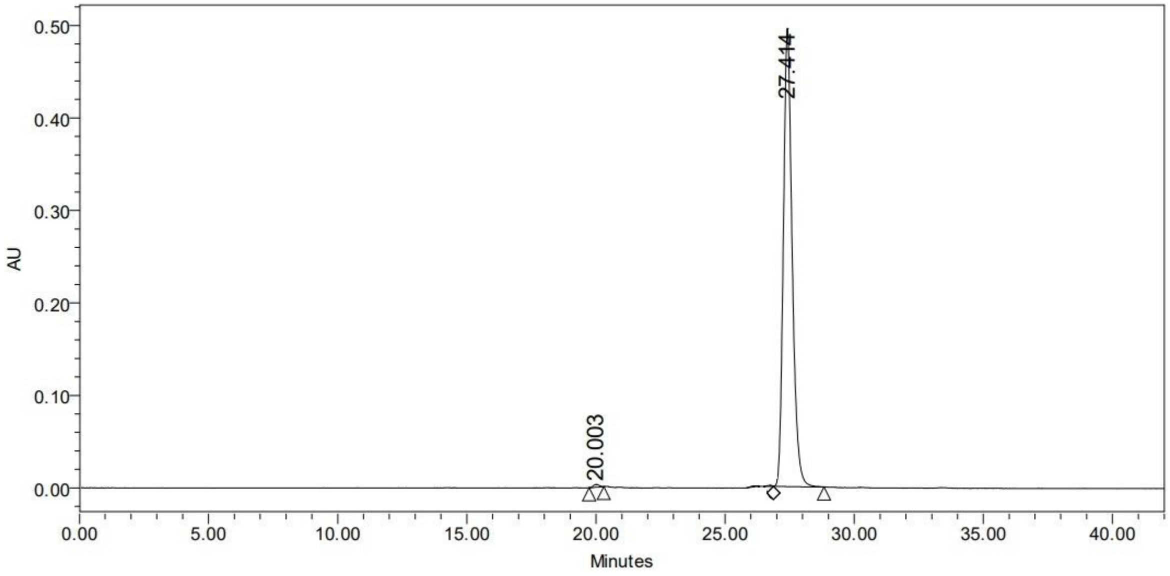

Figure S8. FT-IR spectrum of **2**

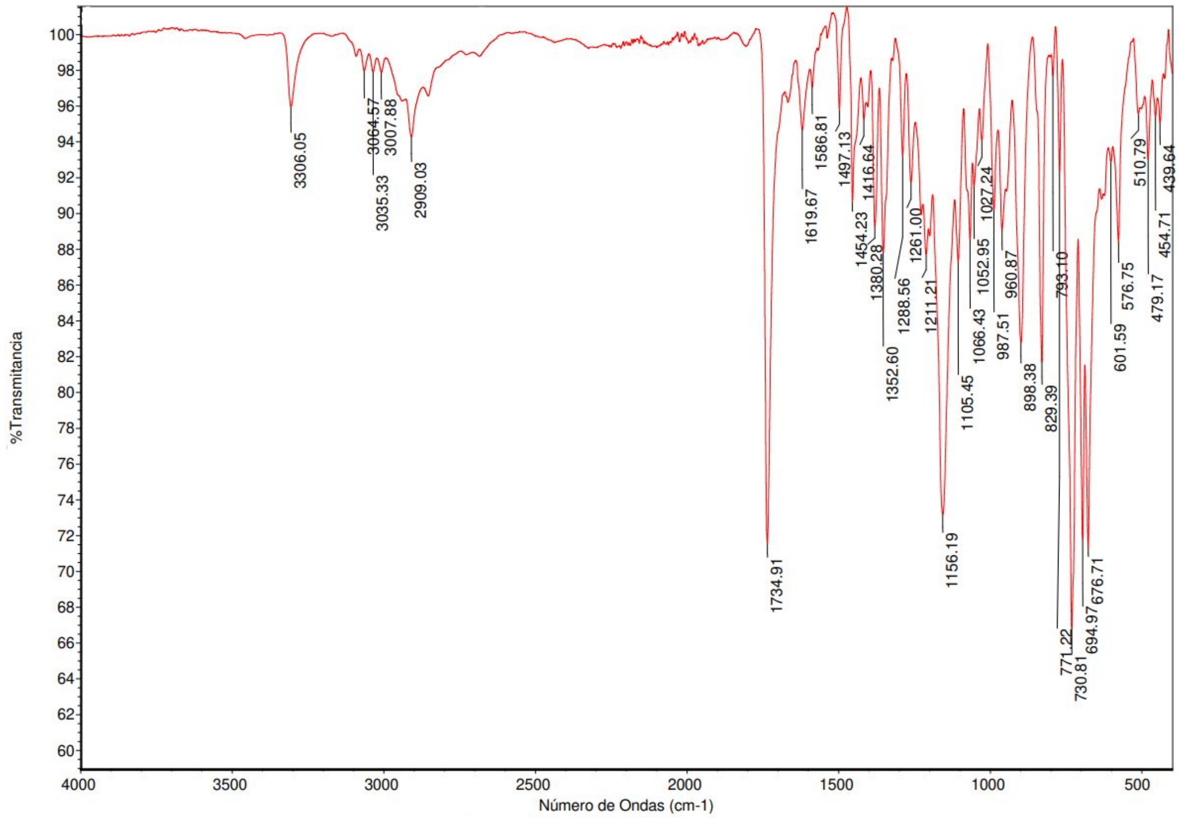

Figure S9. FAB-MS spectrum of **2** (positive detection mode)

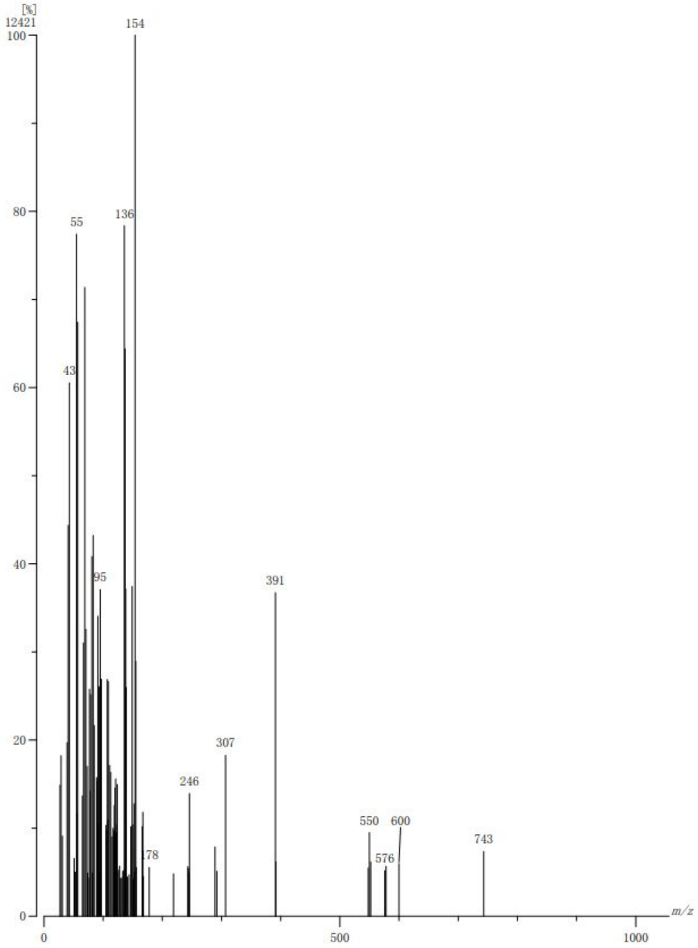

Figure S10. ^{1}H-NMR spectrum of **2** in $CDCl_3$, 300 MHz

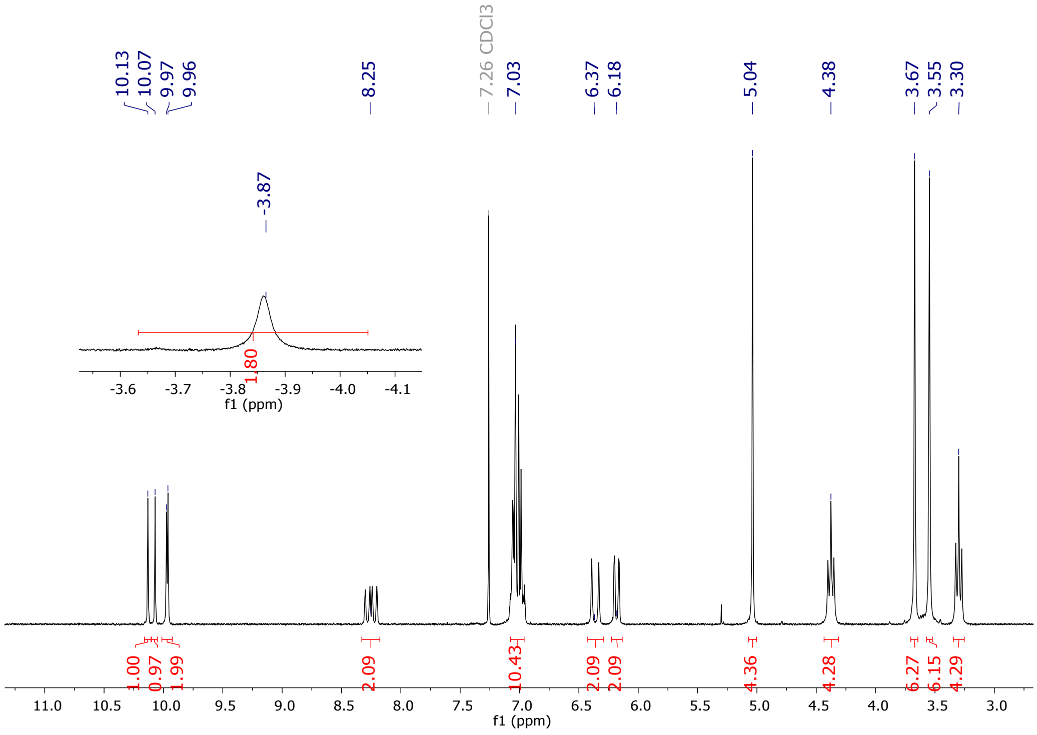

Figure S11. ^{13}C-NMR spectrum of **2** in $CDCl_3$, 300 MHz

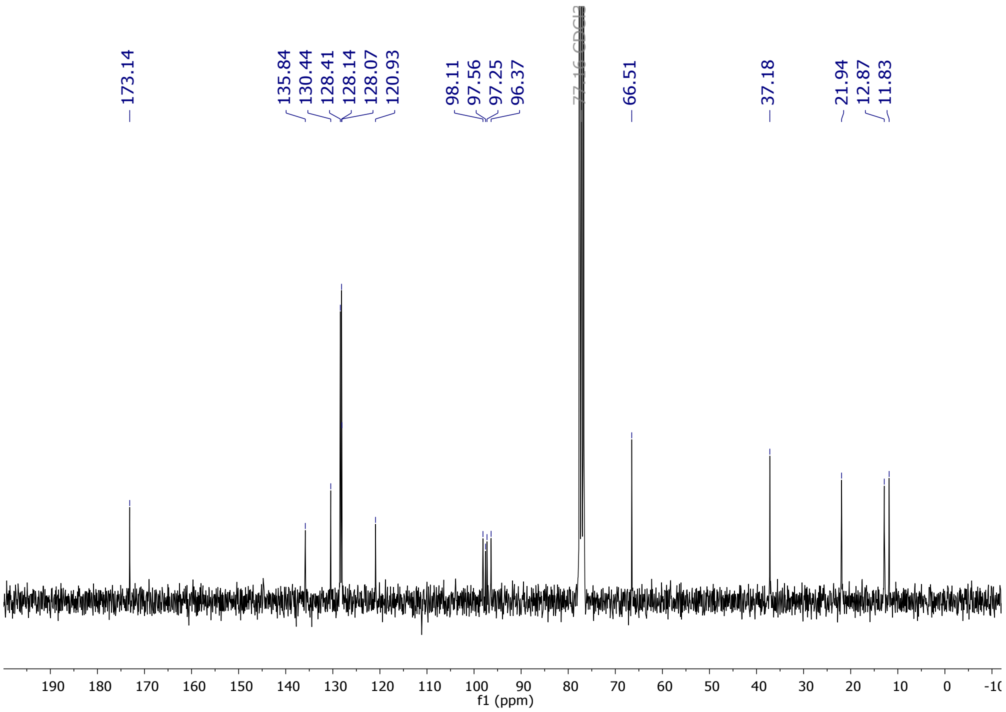

Figure S12. DEP-135 spectrum of **2** in $CDCl_3$, 300 MHz

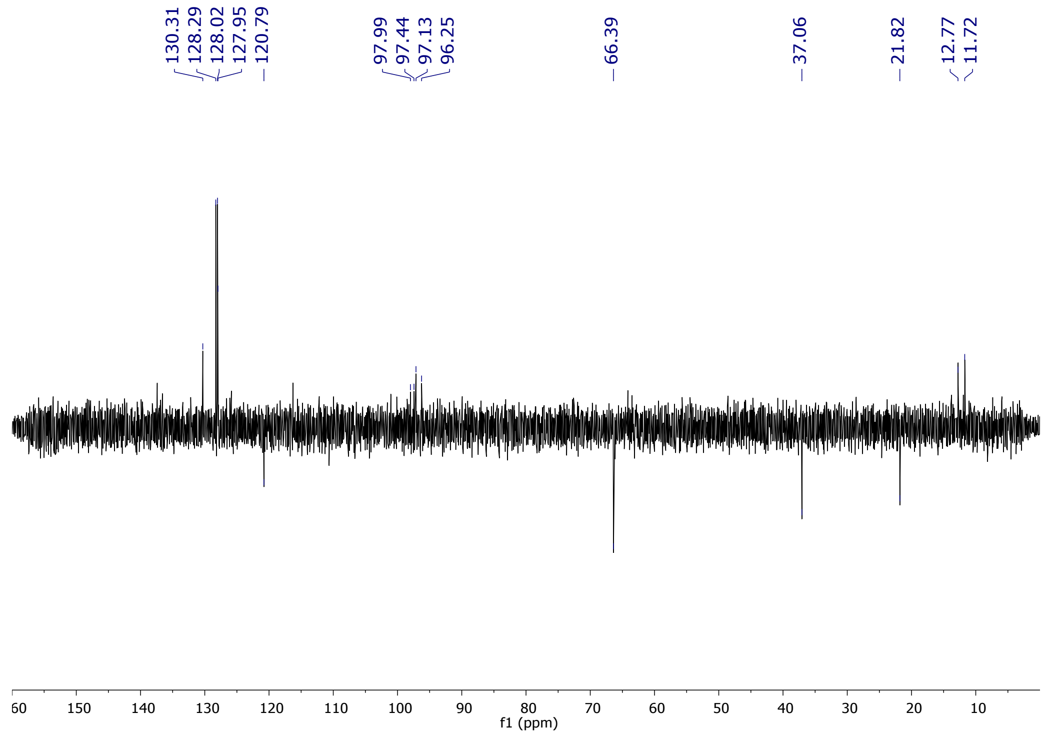

Figure S13. HSQC spectrum of **2** in $CDCl_3$, 300 MHz

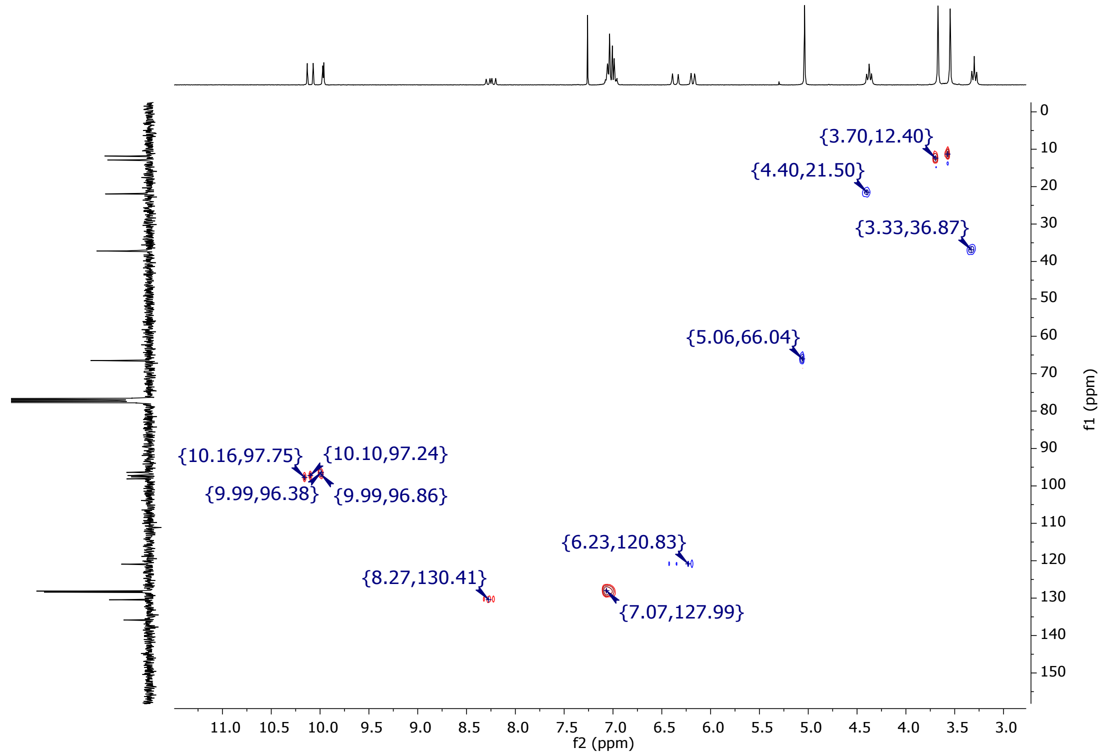

Figure S14. Analytic HPLC of **2** with detection at 408 nm

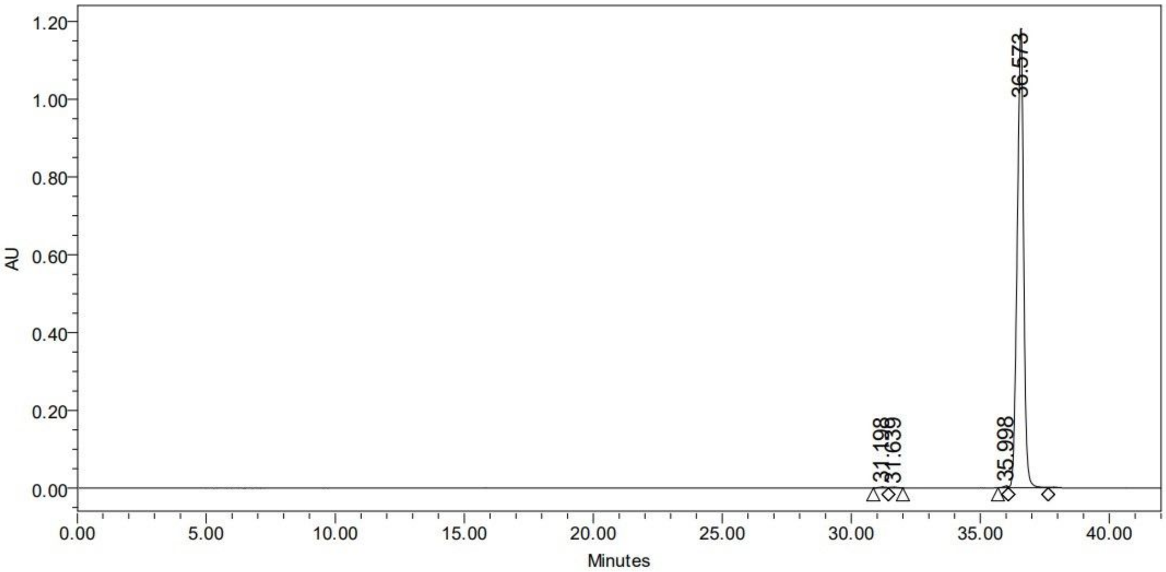

Figure S15. FT-IR spectrum of **3A**

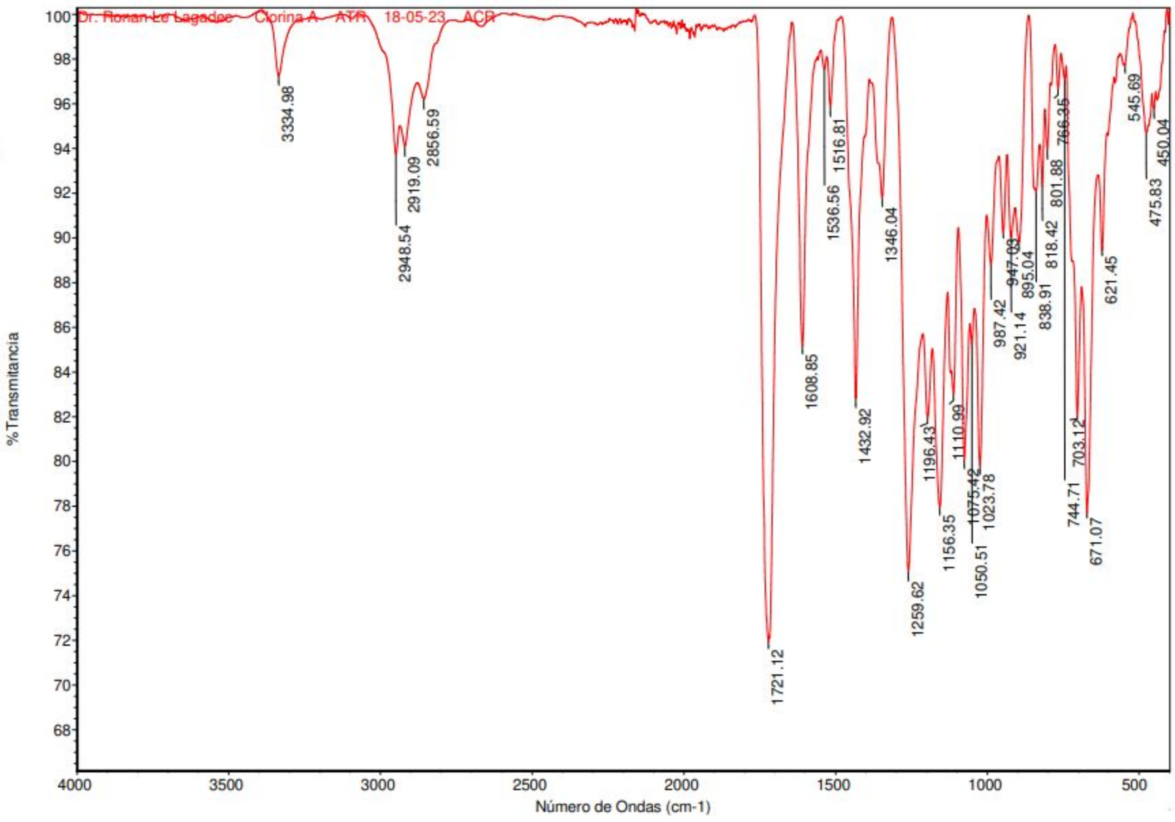

Figure S16. ESI-HRMS spectrum of **3A** (positive detection mode)

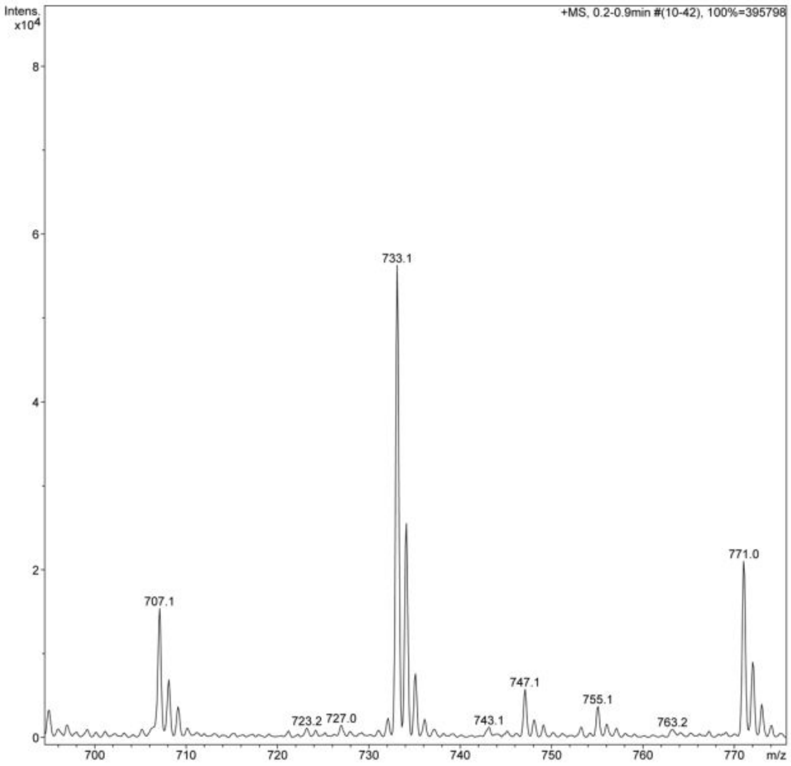

Figure S17. ^{1}H-NMR spectrum of **3A** in $CDCl_3$, 300 MHz

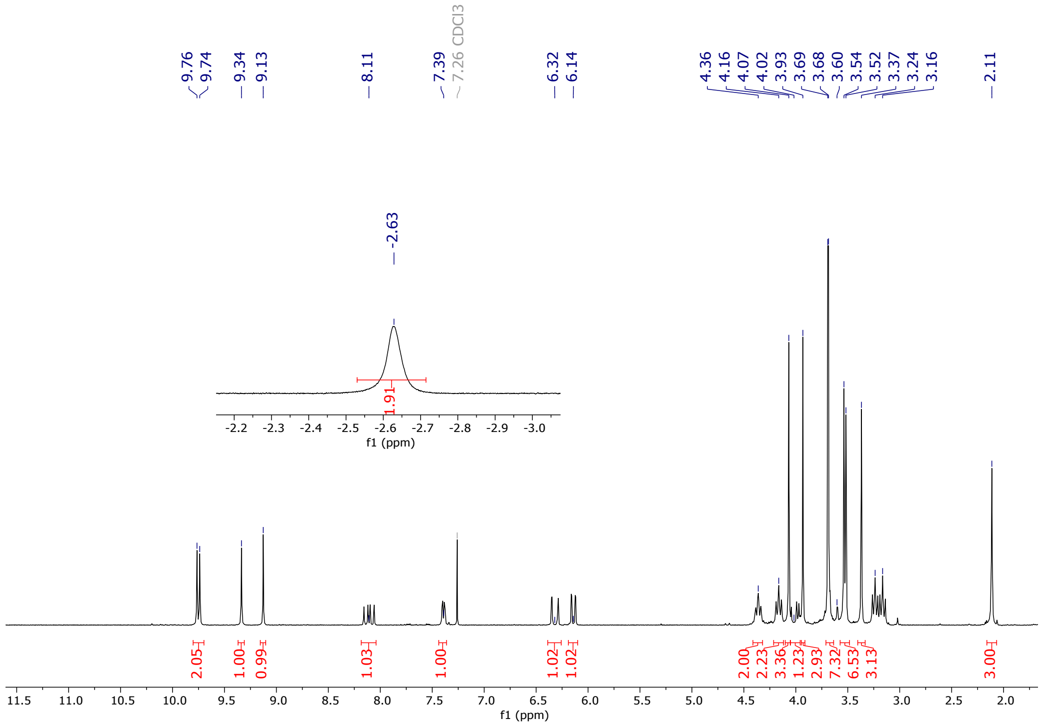

Figure S18. ^{13}C-NMR spectrum of **3A** in $CDCl_3$, 300 MHz

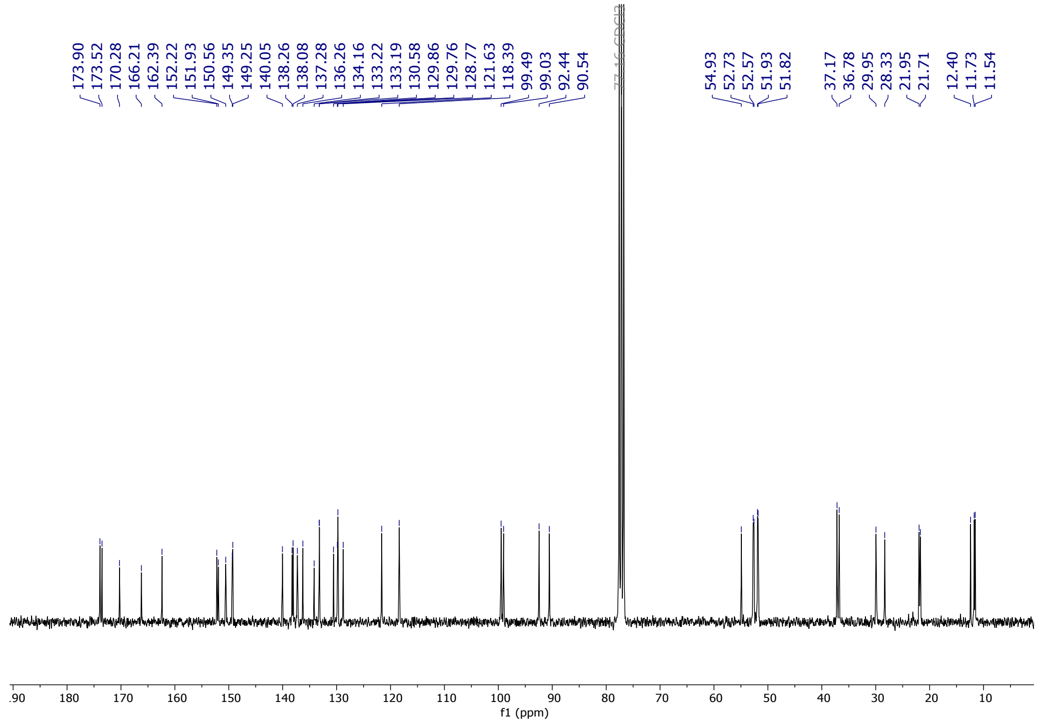

Figure S19. DEP-135 spectrum of **3A** in $CDCl_3$, 300 MHz

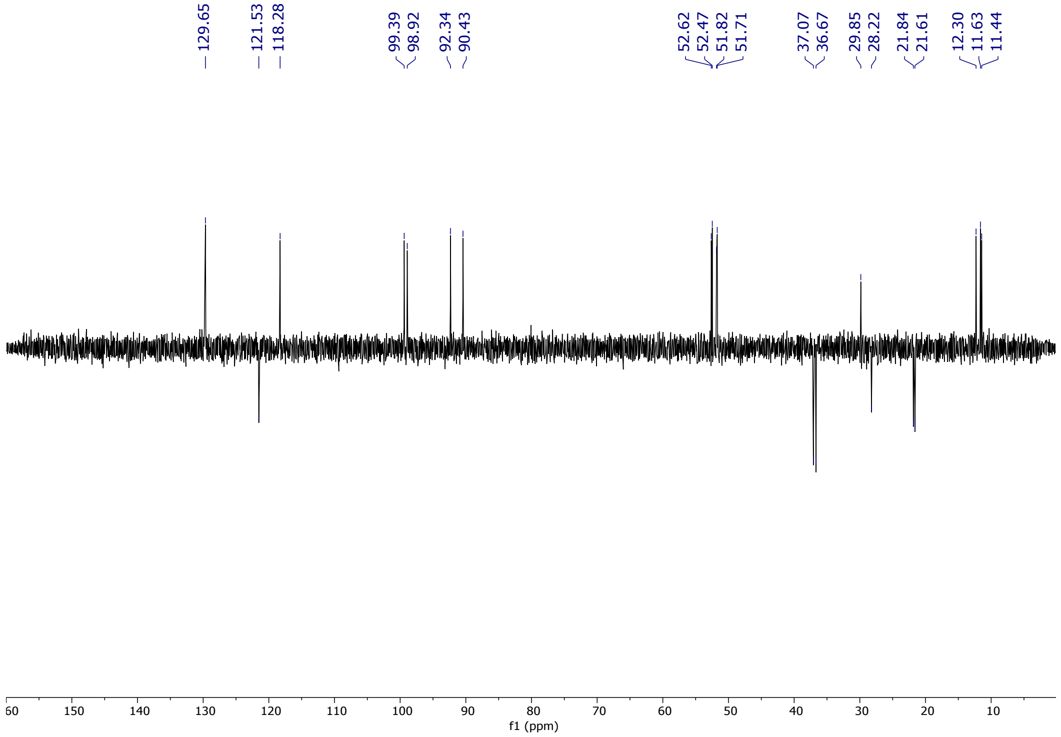

Figure S20. NOESY spectrum of **3A** in $CDCl_3$, 300 MHz

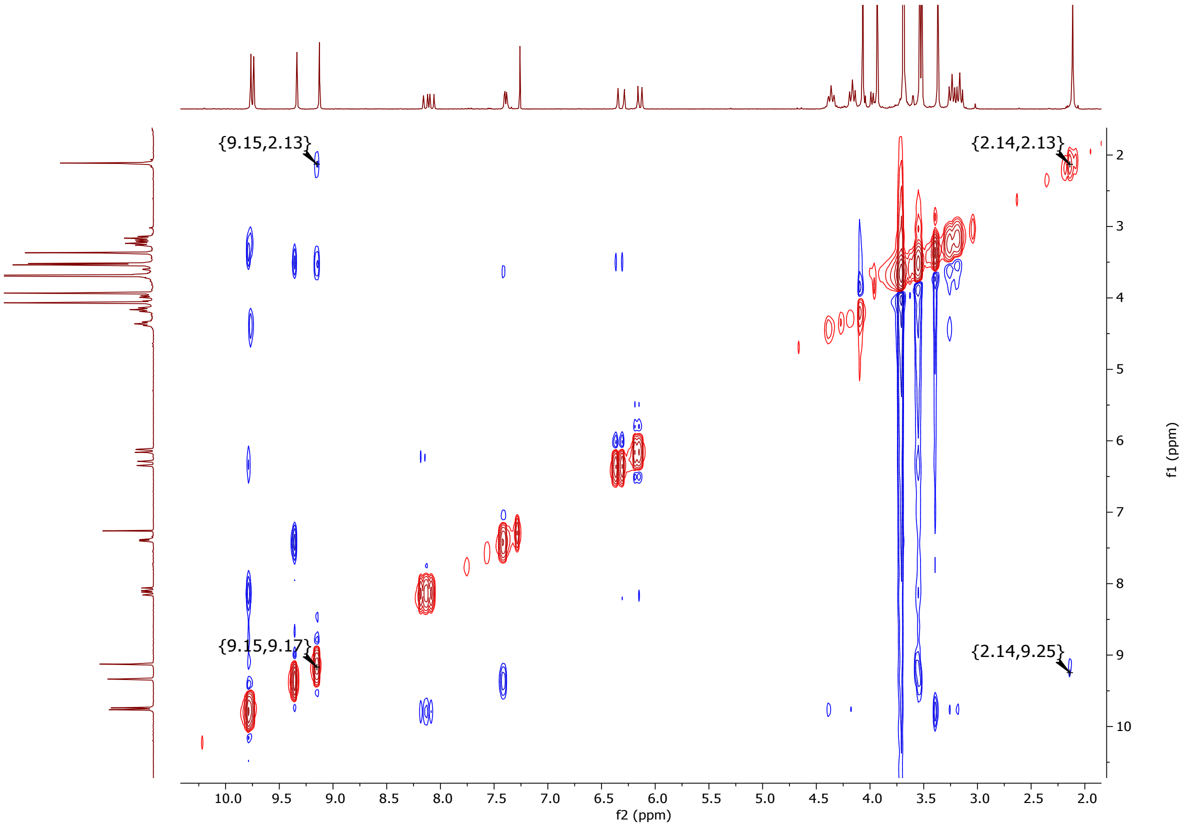

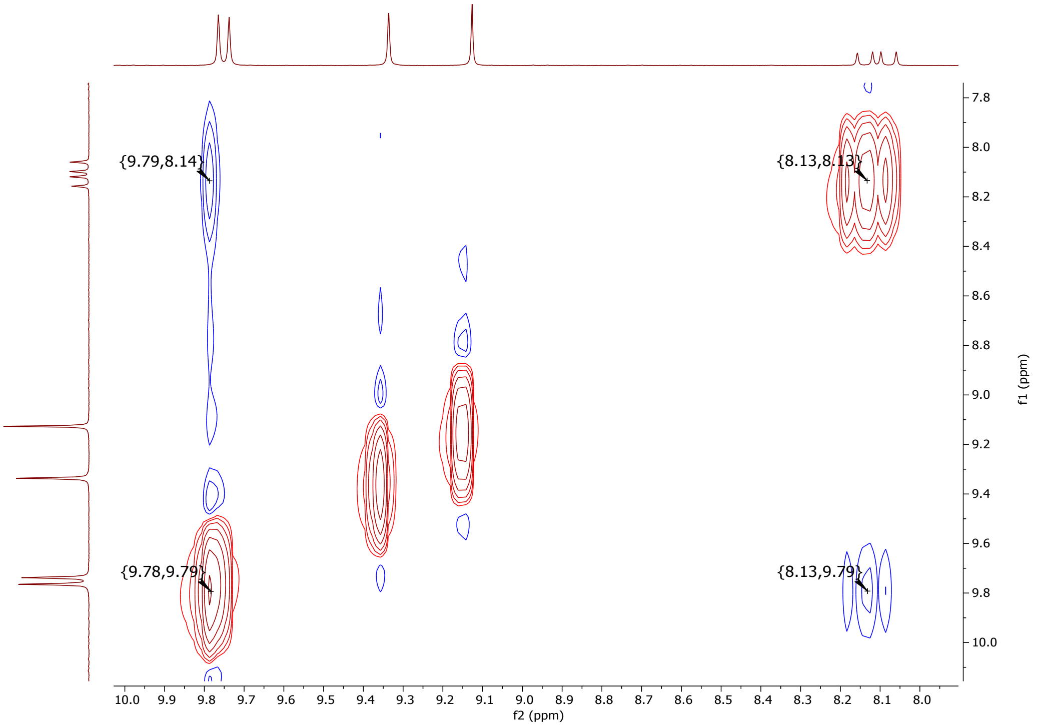

Figure S21. Analytic HPLC of **3A** with detection at 406 nm

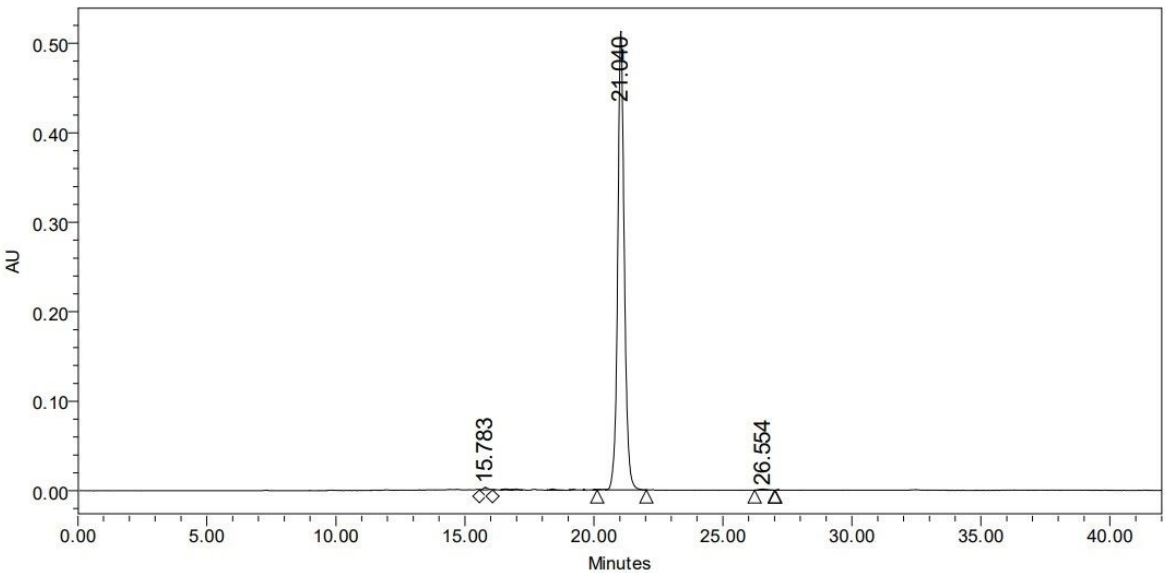

Figure S22. FT-IR spectrum of **3B**

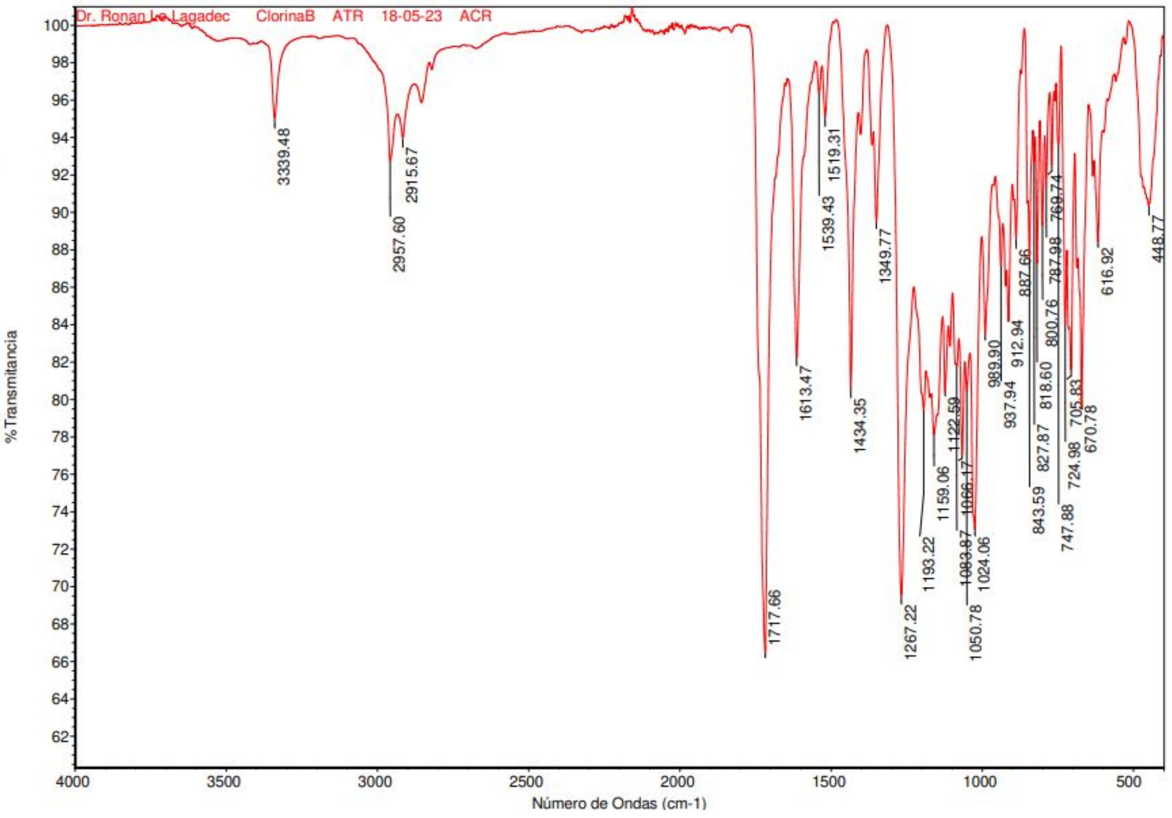

Figure S23. ESI-HRMS spectrum of **3B** (positive detection mode)

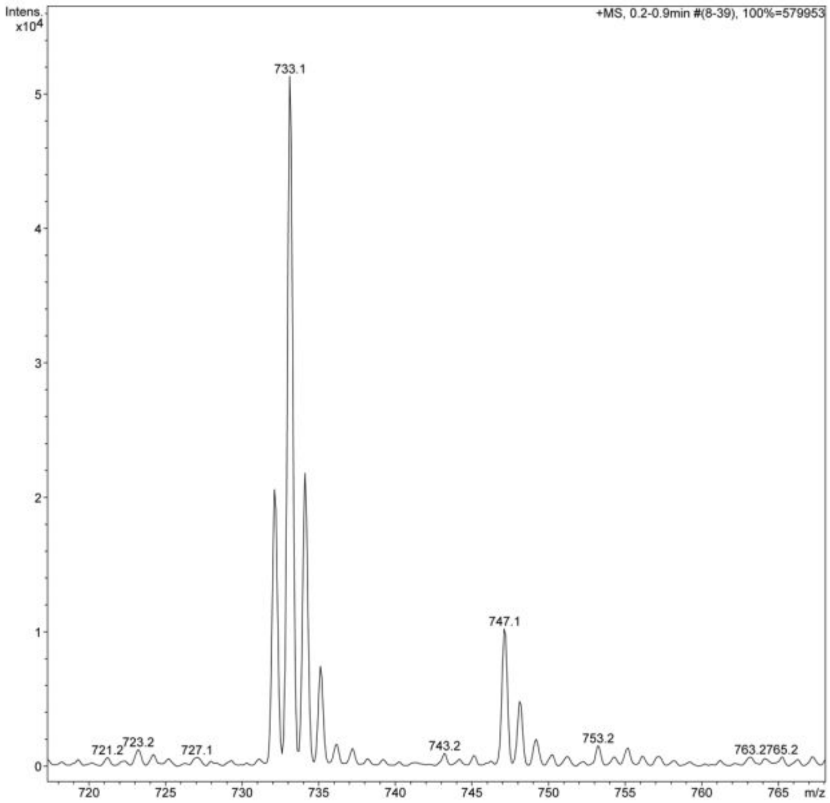

Figure S24. ^{1}H-NMR spectrum of **4B** in $CDCl_3$, 300 MHz

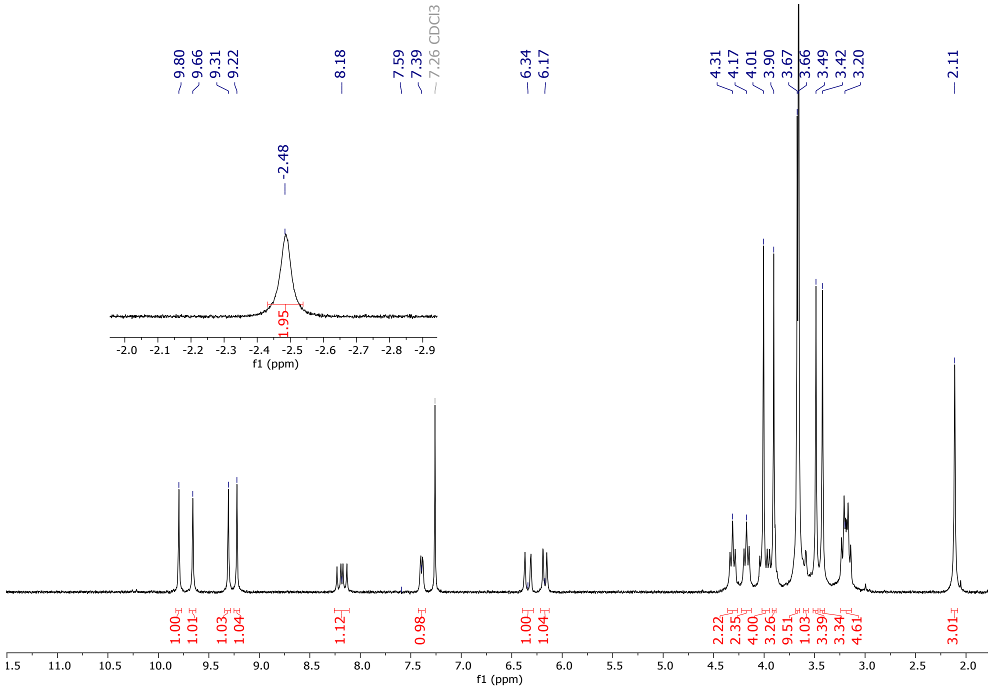

Figure S25. ^{13}C-NMR spectrum of **3B** in $CDCl_3$, 300 MHz

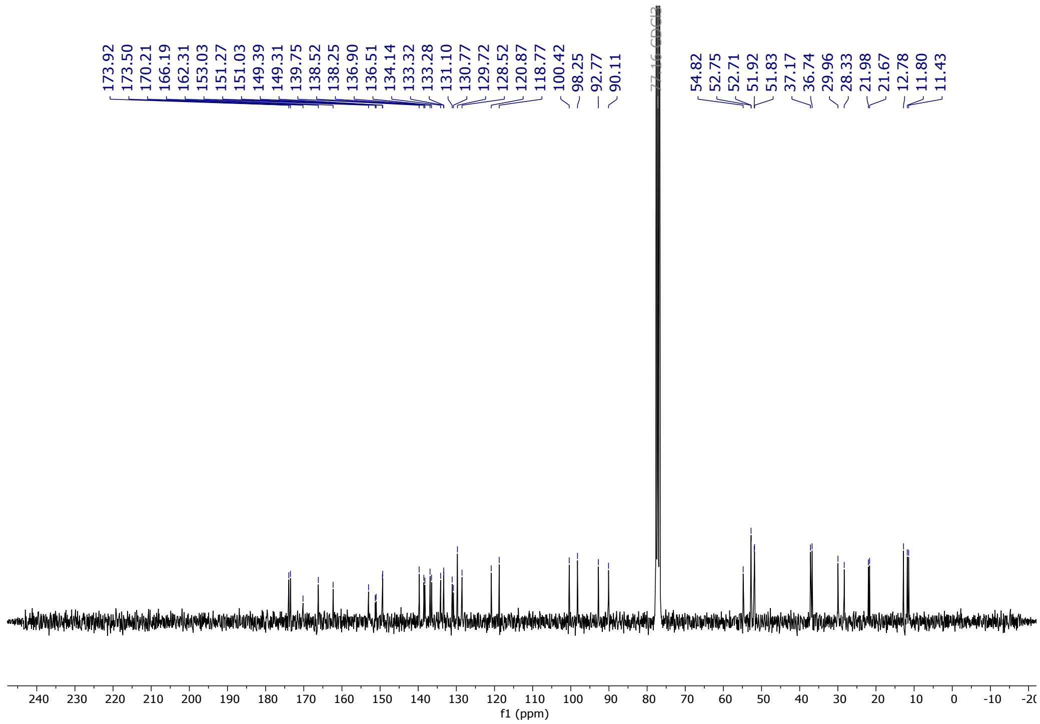

Figure S26. DEP-135 spectrum of **3B** in $CDCl_3$, 300 MHz

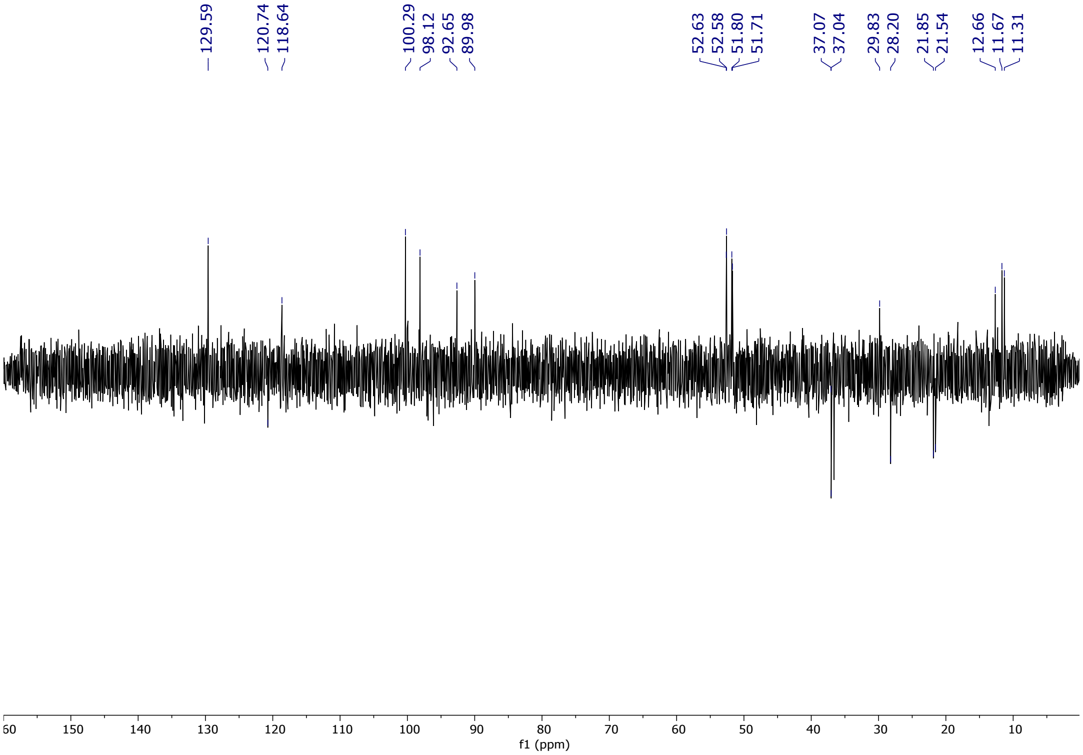

Figure S27. NOESY spectrum of **3B** in $CDCl_3$, 300 MHz

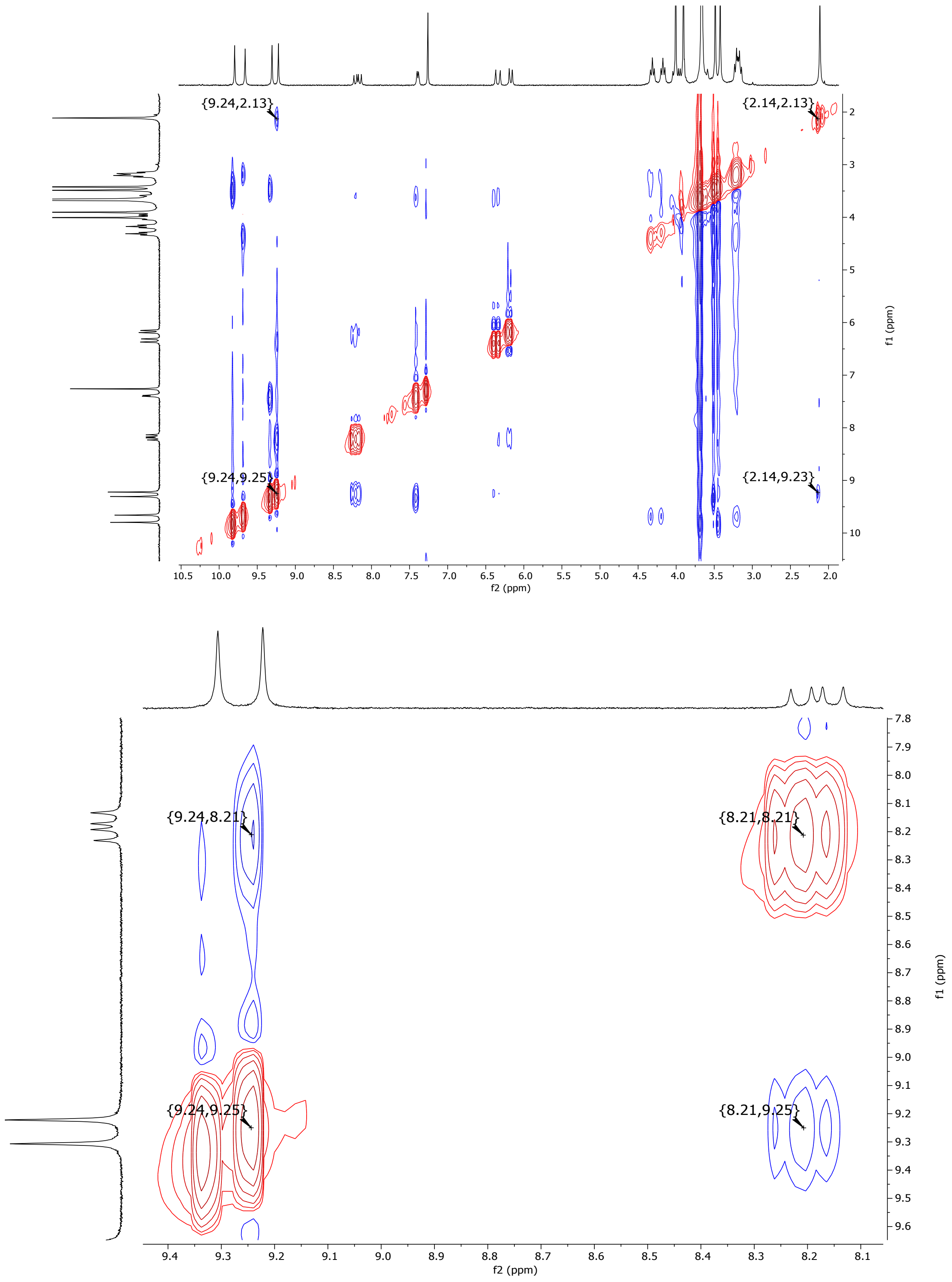

Figure S28. Analytic HPLC of **3B** with detection at 404 nm

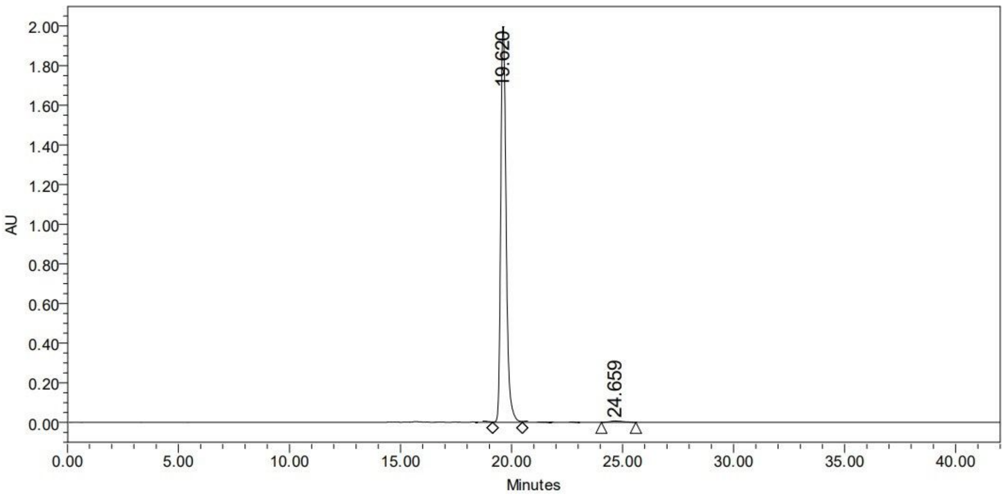

Figure S29. FT-IR spectrum of **4A**

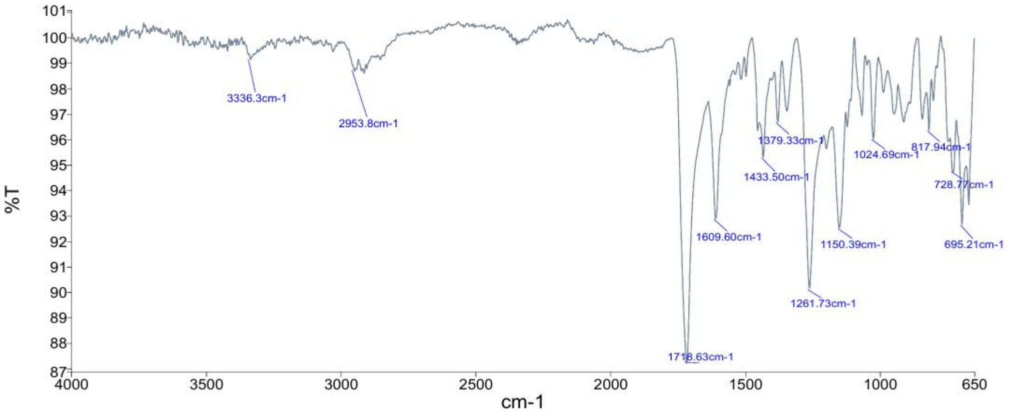

Figure S30. ESI-HRMS spectrum of **4A** (positive detection mode)

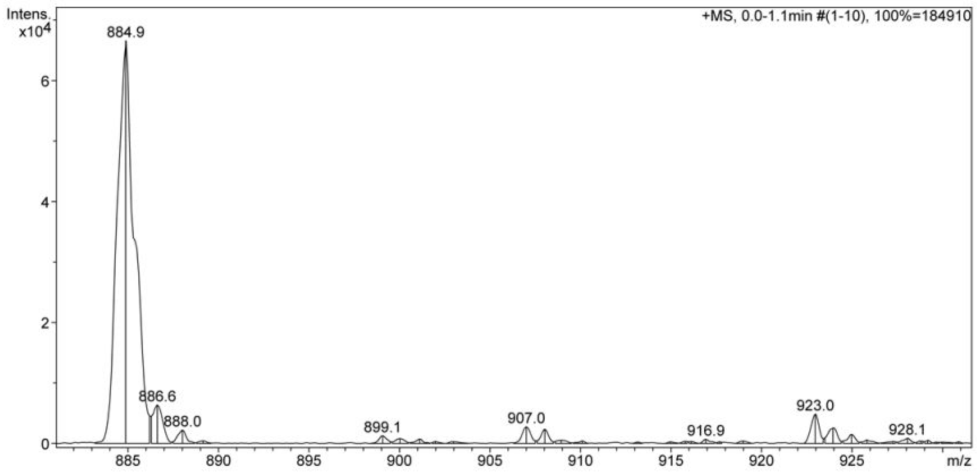

Figure S31. ^{1}H-NMR spectrum of **4A** in $CDCl_3$, 300 MHz

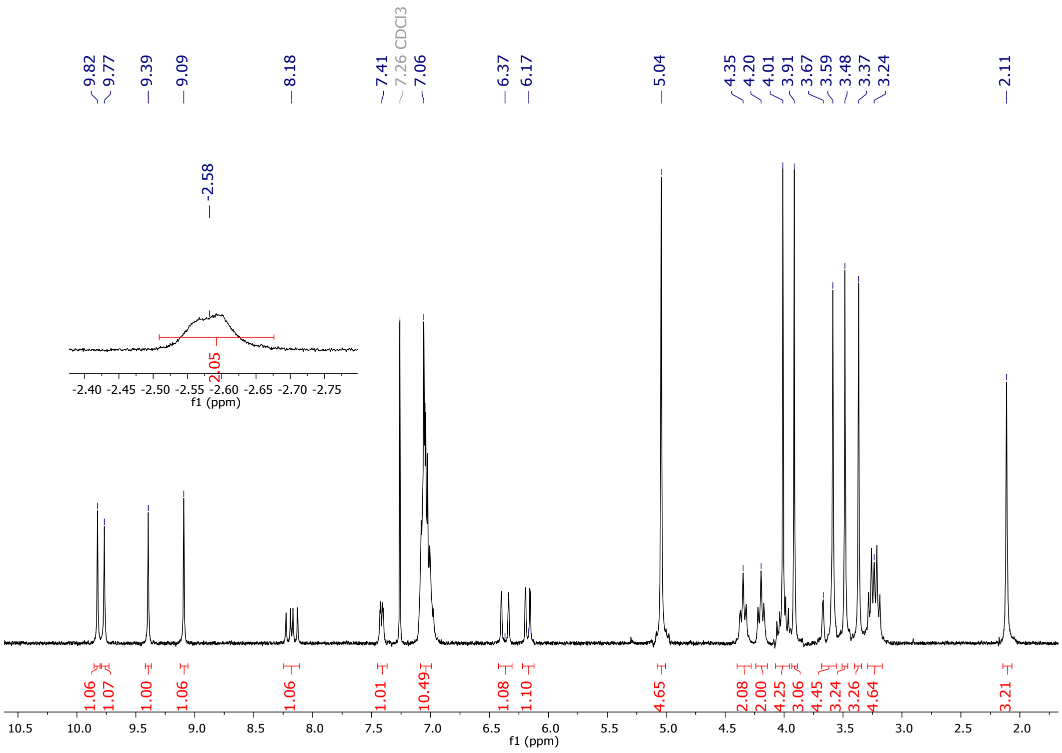

Figure S32. ^{13}C-NMR spectrum of **4A** in $CDCl_3$, 300 MHz

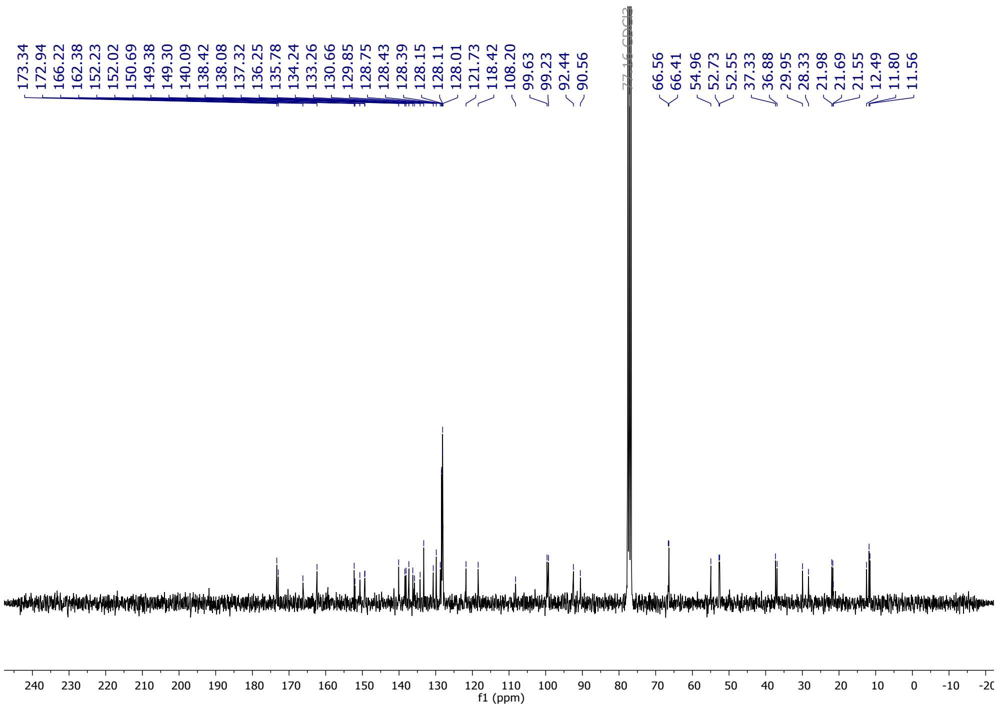

Figure S33. DEP-135 spectrum of **4A** in $CDCl_3$, 300 MHz

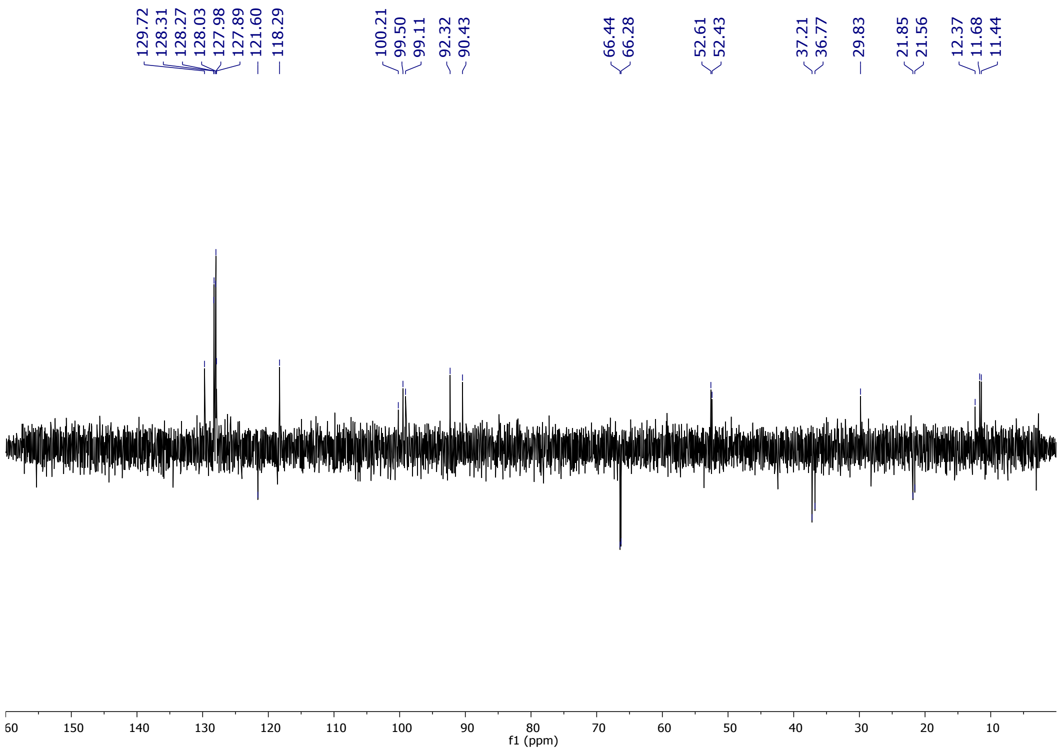

Figure S34. NOESY spectrum of **4A** in $CDCl_3$, 300 MHz

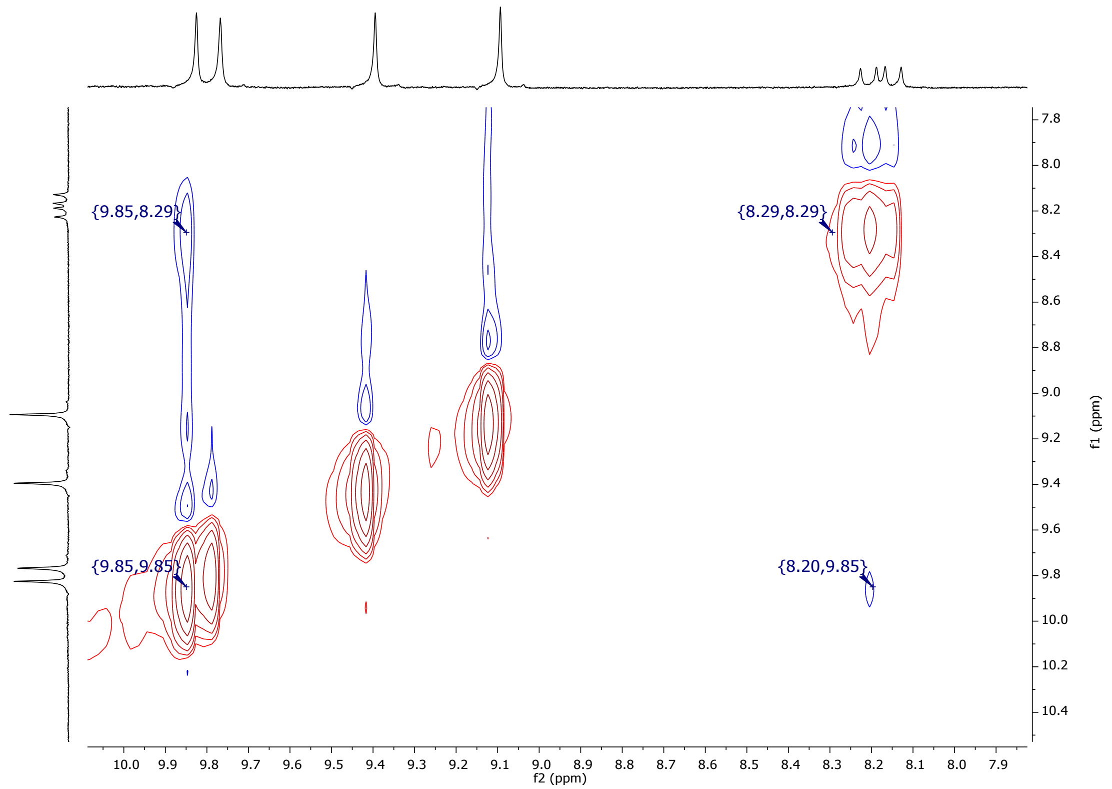

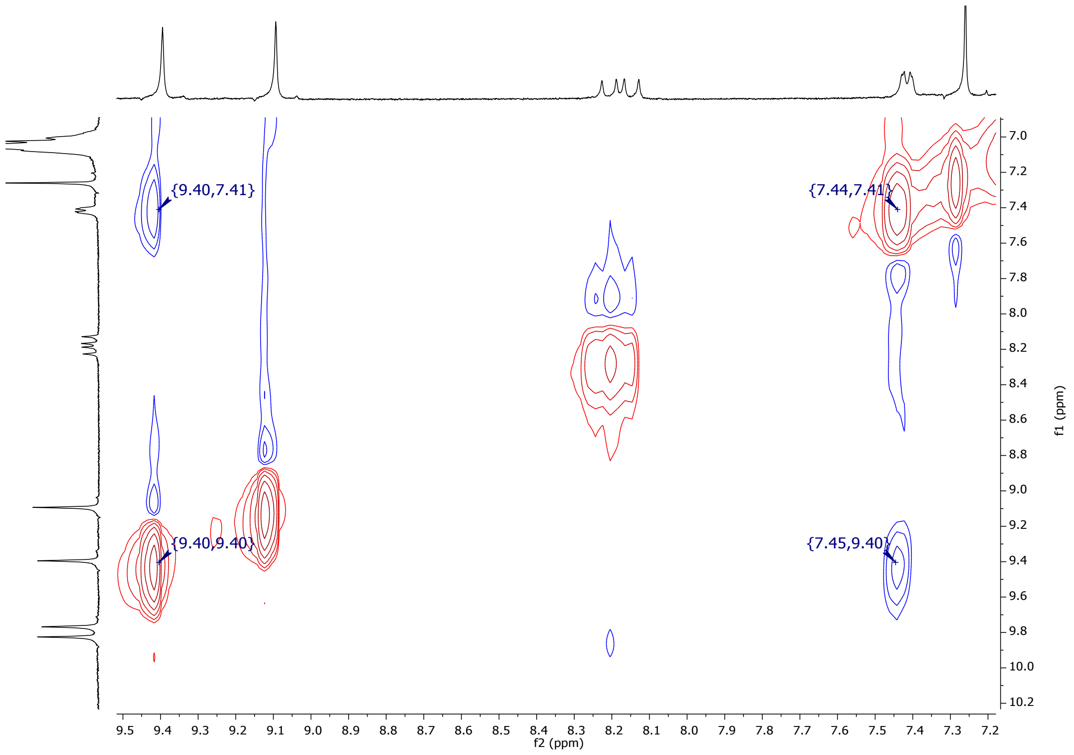

Figure S35. Analytic HPLC of **4A** with detection at 406 nm

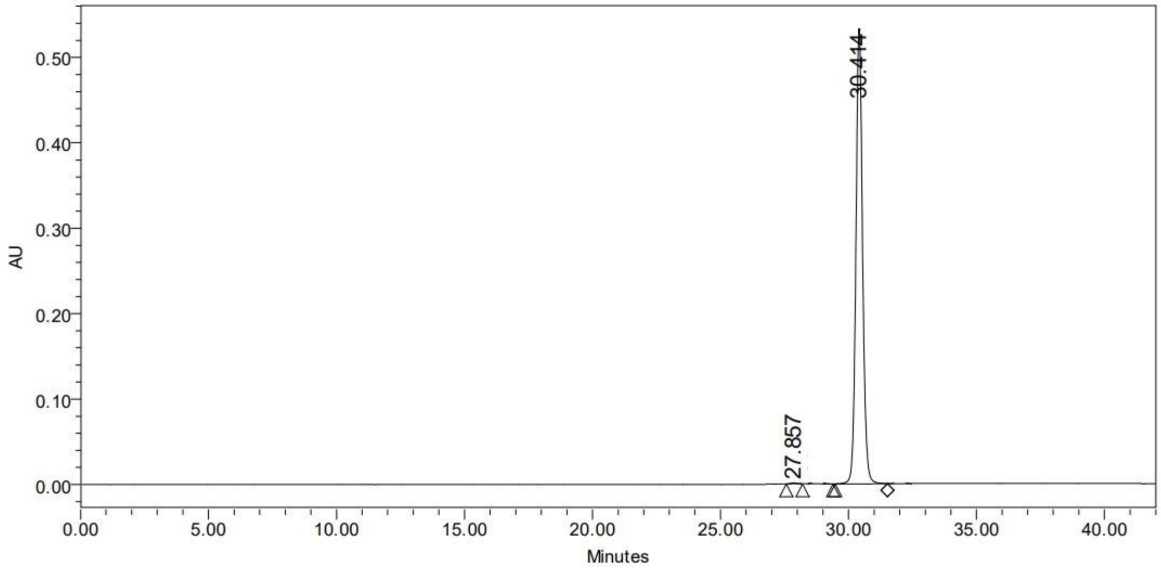

Figure S36. FT-IR spectrum of **4B**

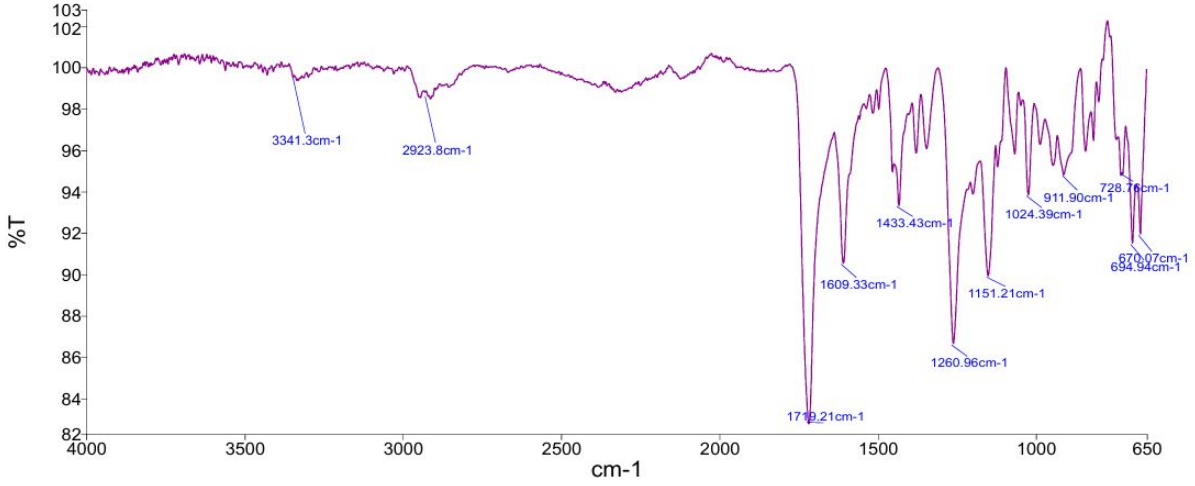

Figure S37. ESI-HRMS spectrum of **4B** (positive detection mode)

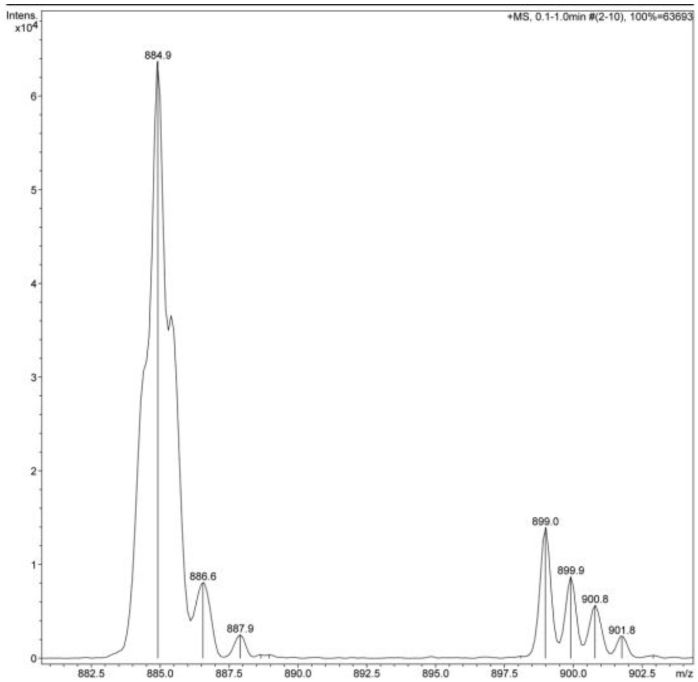

Figure S38. ^{1}H-NMR spectrum of **4B** in $CDCl_3$, 300 MHz

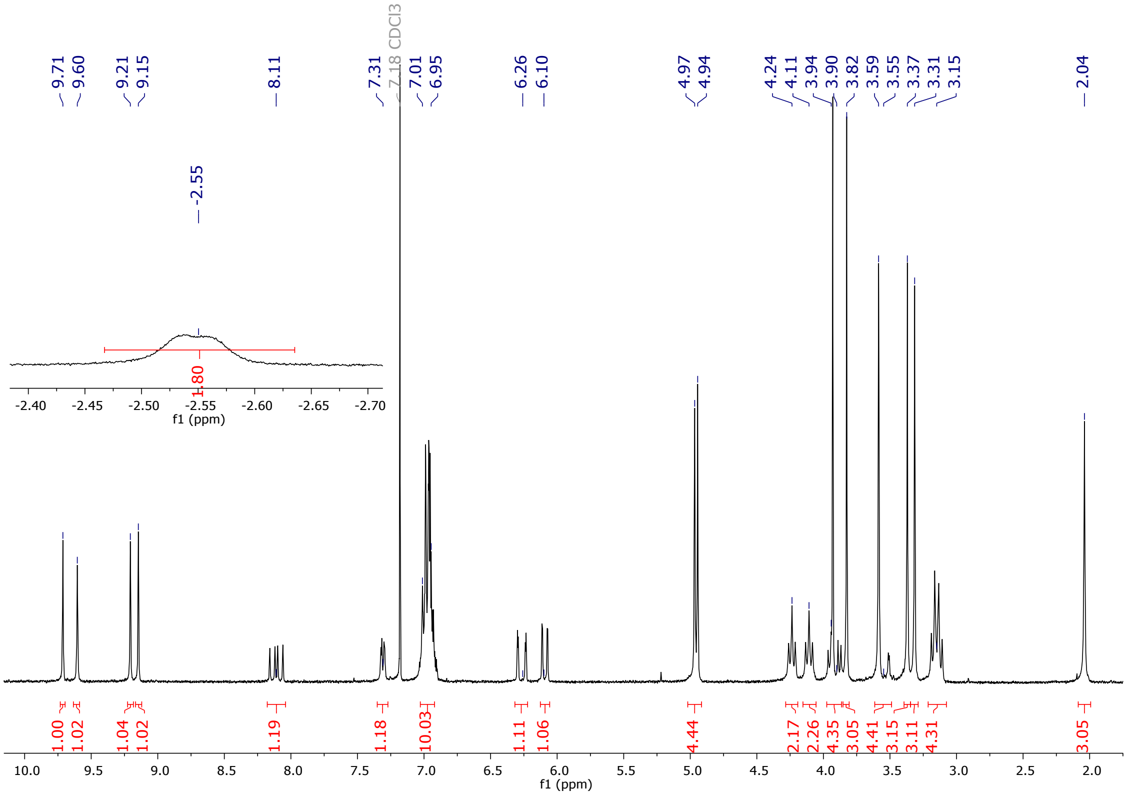

Figure S39. ^{13}C-NMR spectrum of **4B** in $CDCl_3$, 300 MHz

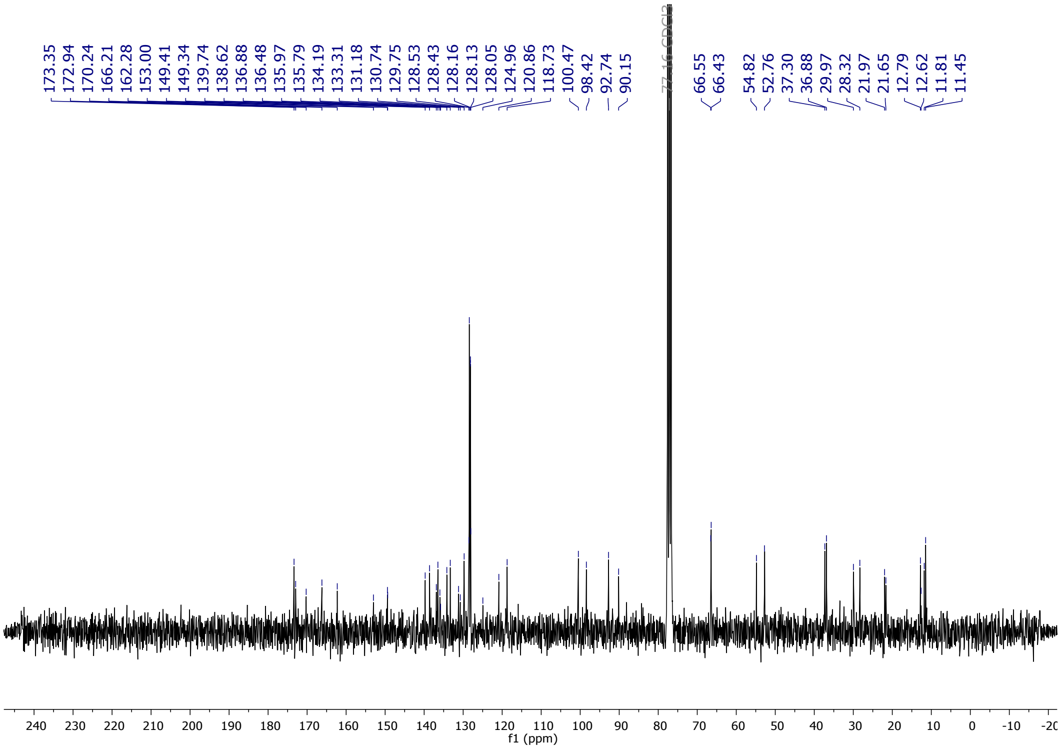

Figure S40. NOESY spectrum of **4B** in $CDCl_3$, 300 MHz

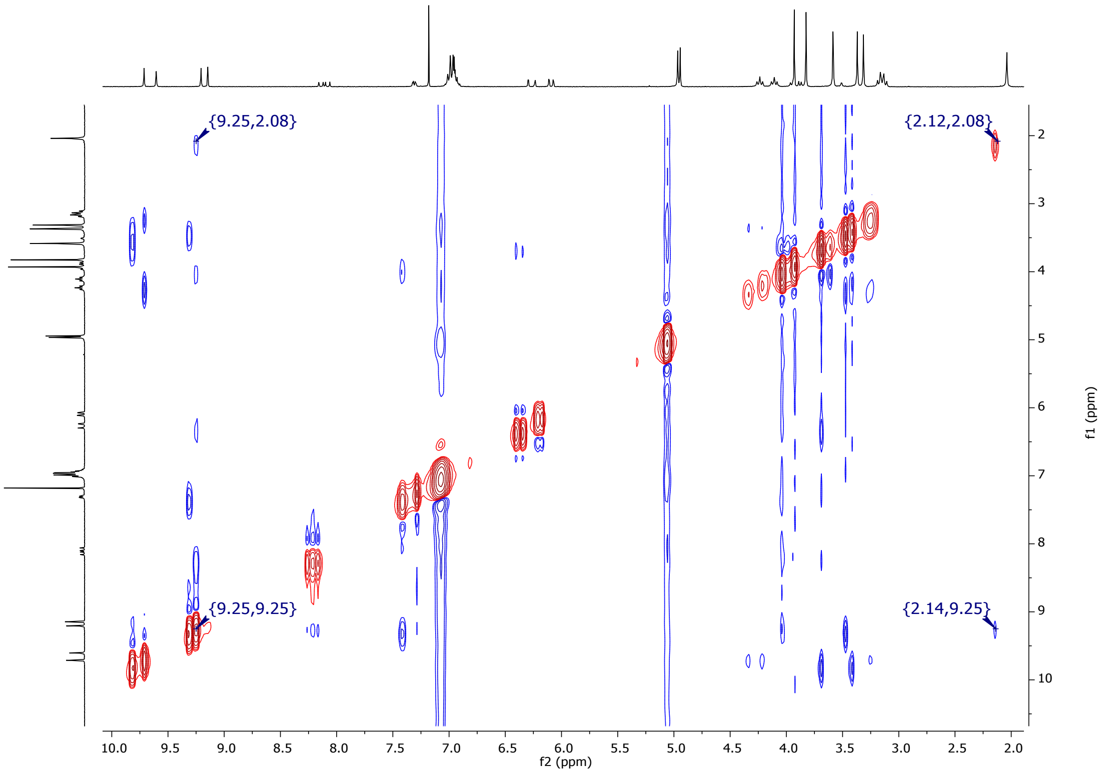

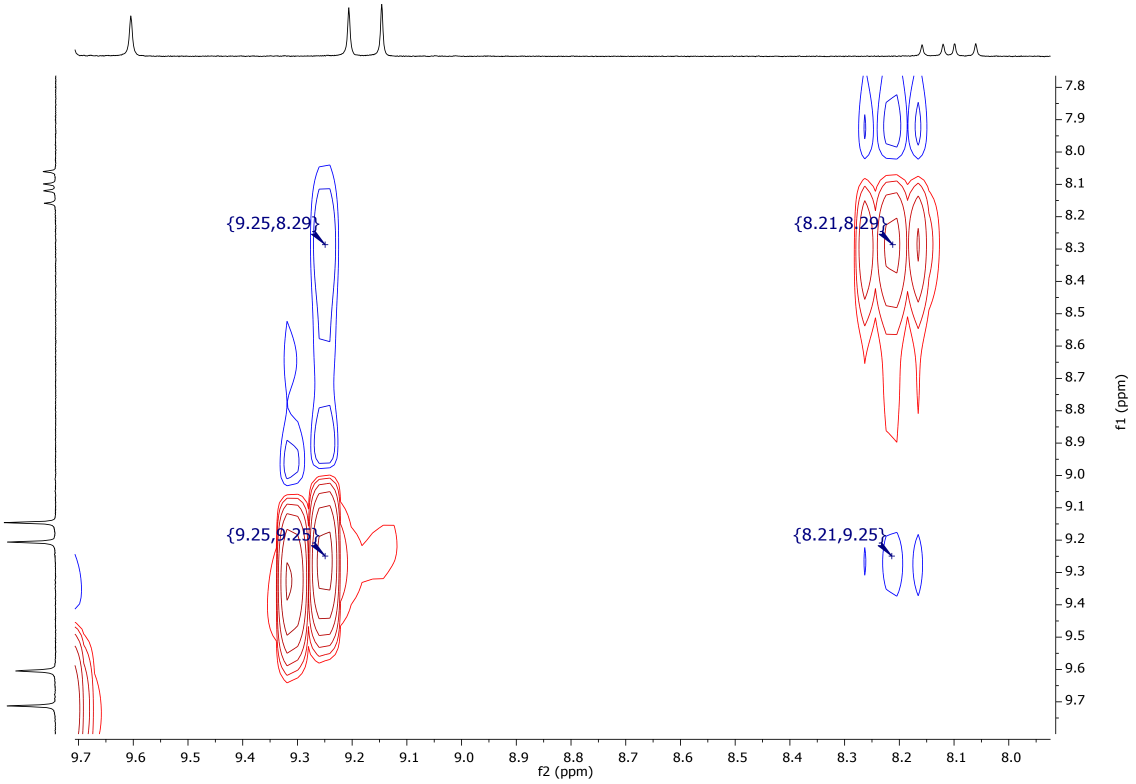

Figure S41. Analytic HPLC of **4B** with detection at 406 nm

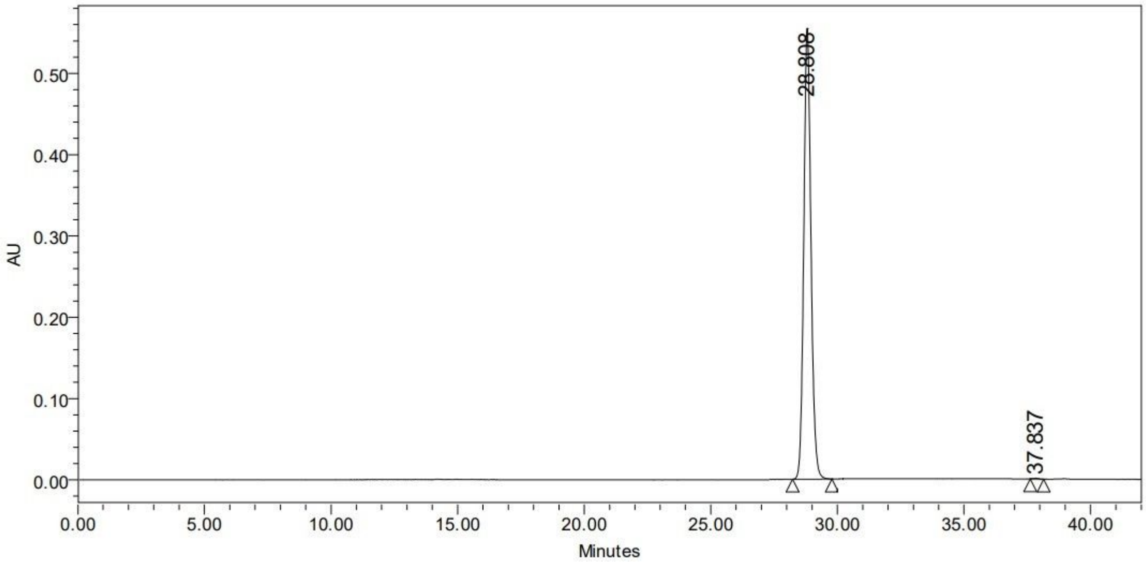

Figure S42. FT-IR spectrum of **Ru-1**

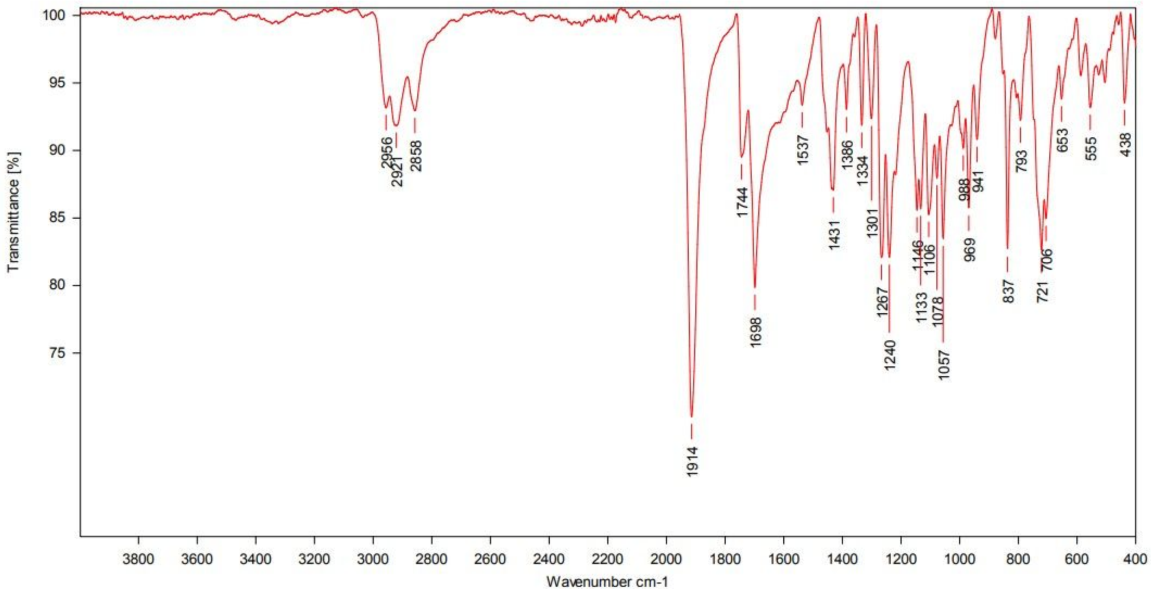

Figure S43. FAB-MS spectrum of **Ru-1** (positive detection mode)

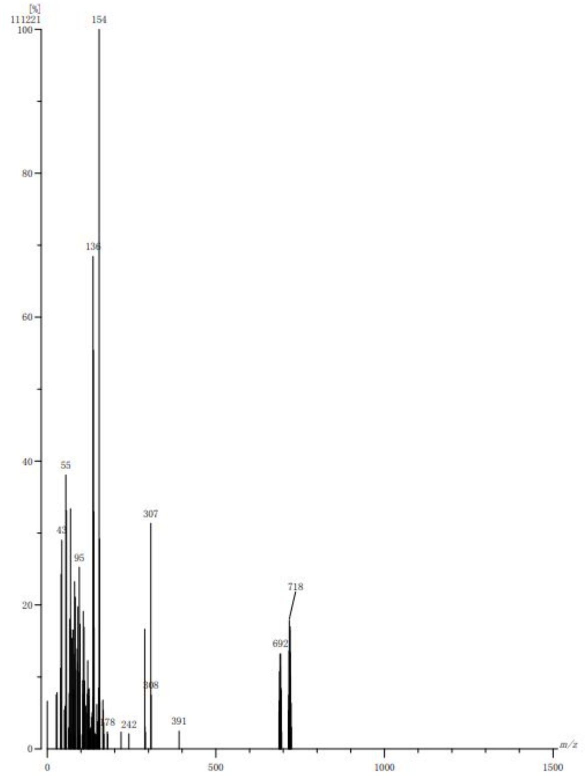

Figure S44. ^{1}H-NMR spectrum of **Ru-1** in CD_3CN, 500 MHz

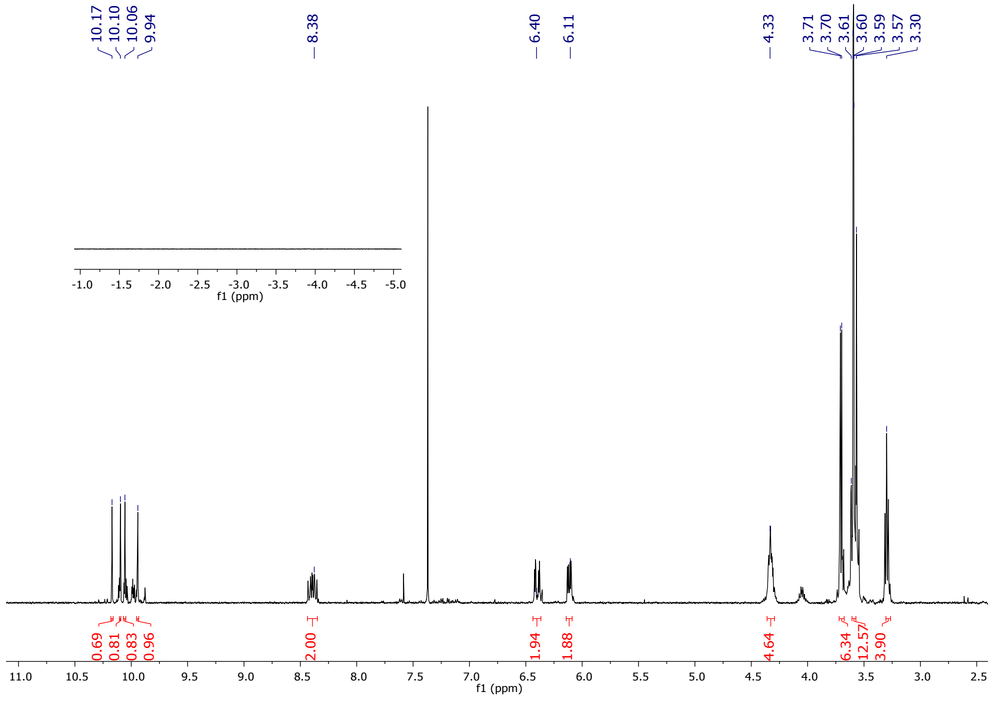

Figure S45. ^{13}C-NMR spectrum of **Ru-1** in CD_3CN, 125 MHz

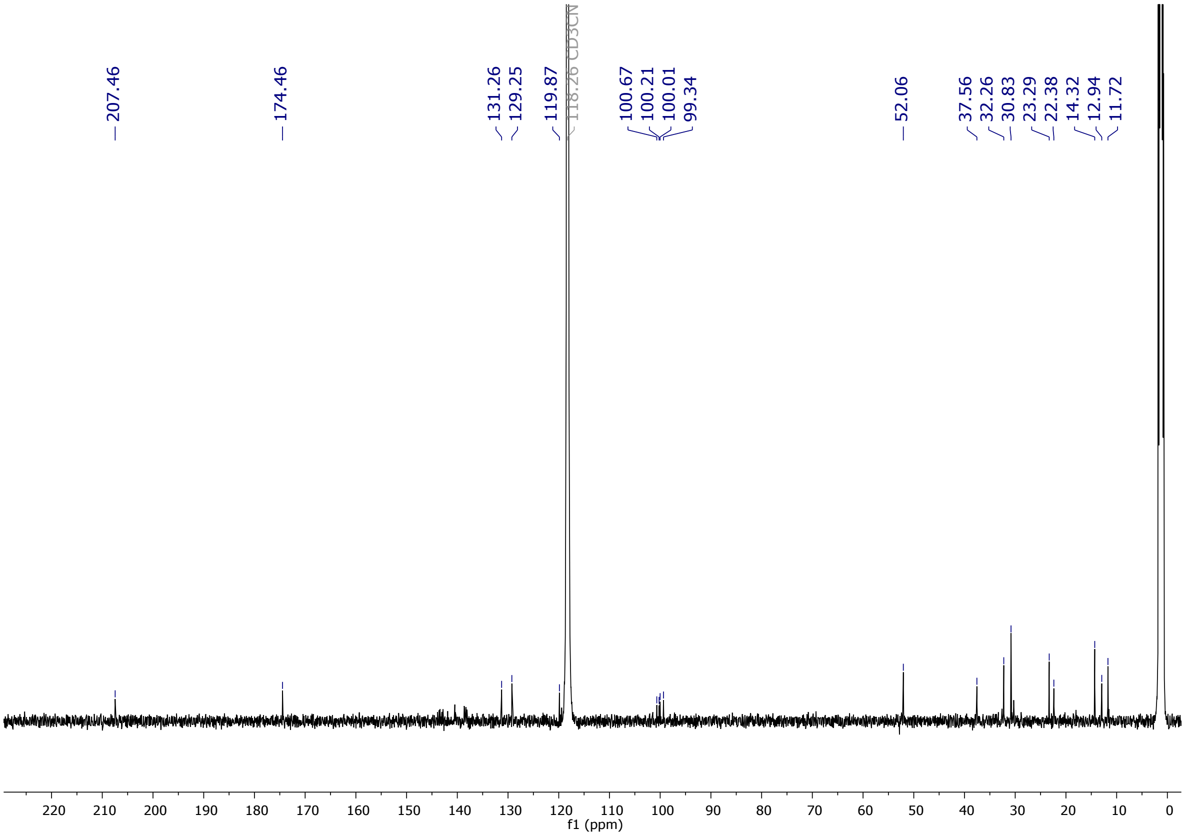

Figure S46. Analytic HPLC of **Ru-1** with detection at 404 nm

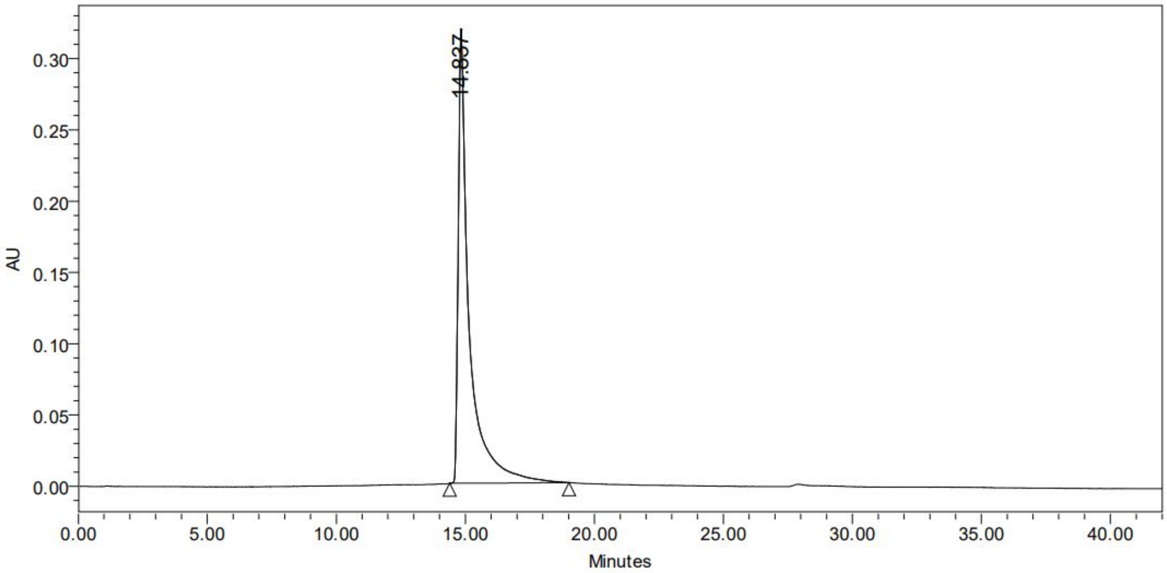

Figure S47. FT-IR spectrum of **Ru-2**

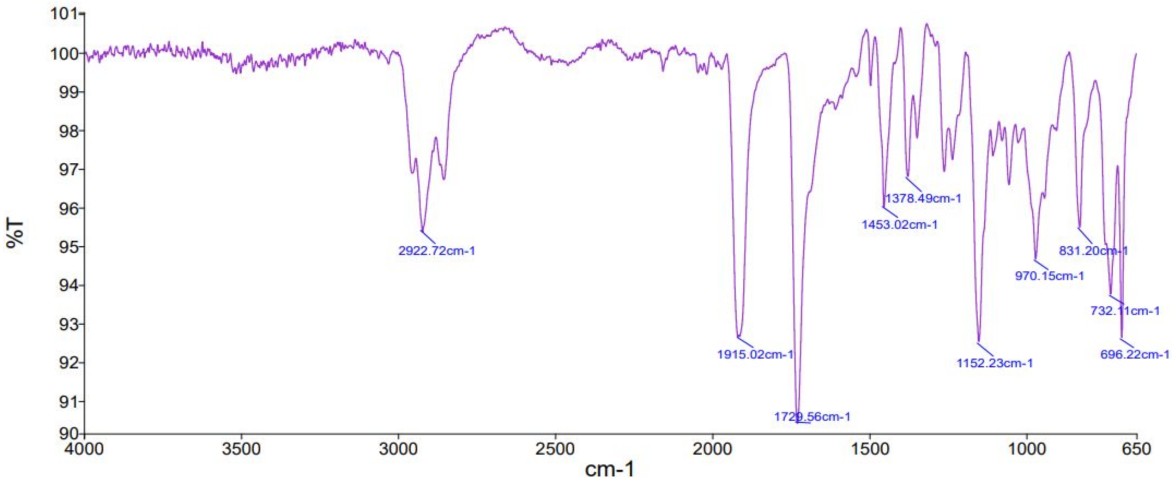

Figure S48. ESI-HRMS spectrum of **Ru-2** (positive detection mode)

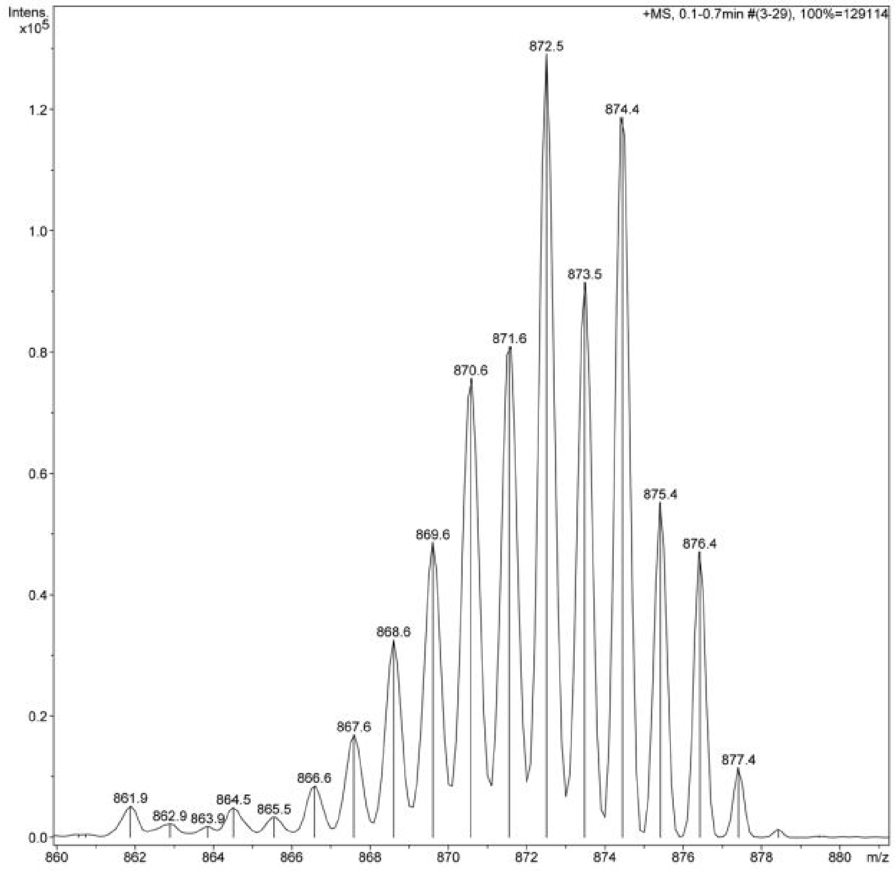

Figure S49. ^{1}H-NMR spectrum of **Ru-2** in CD_3CN, 500 MHz

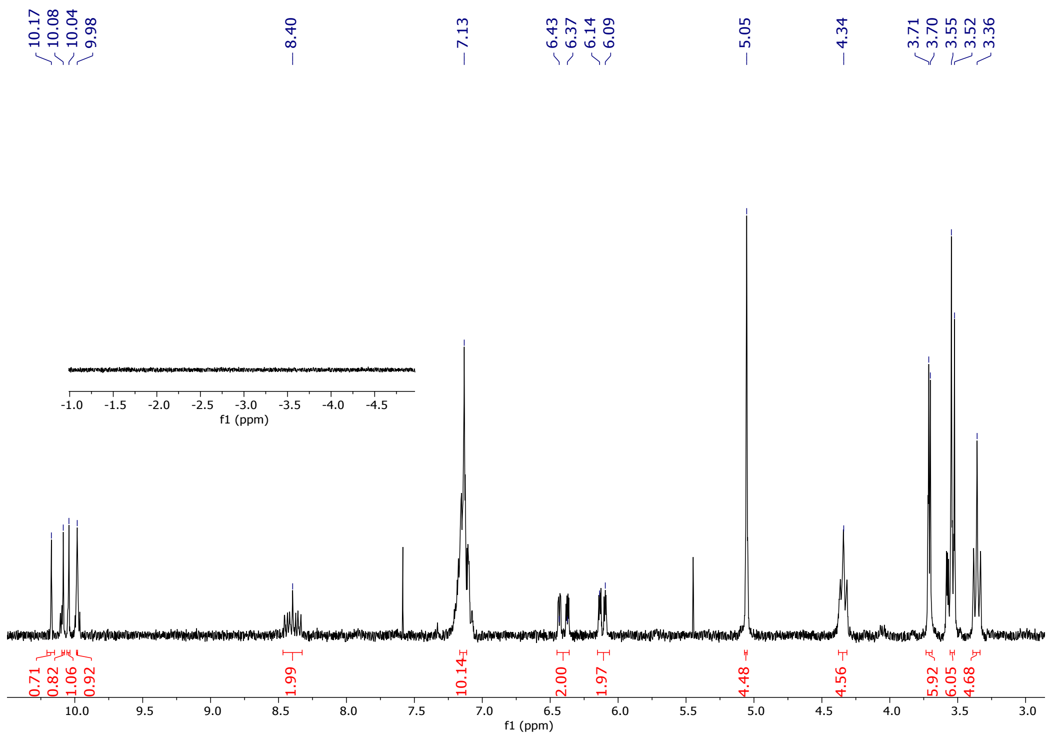

Figure S50. ^{13}C-NMR spectrum of **Ru-2** in CD_3CN, 125 MHz

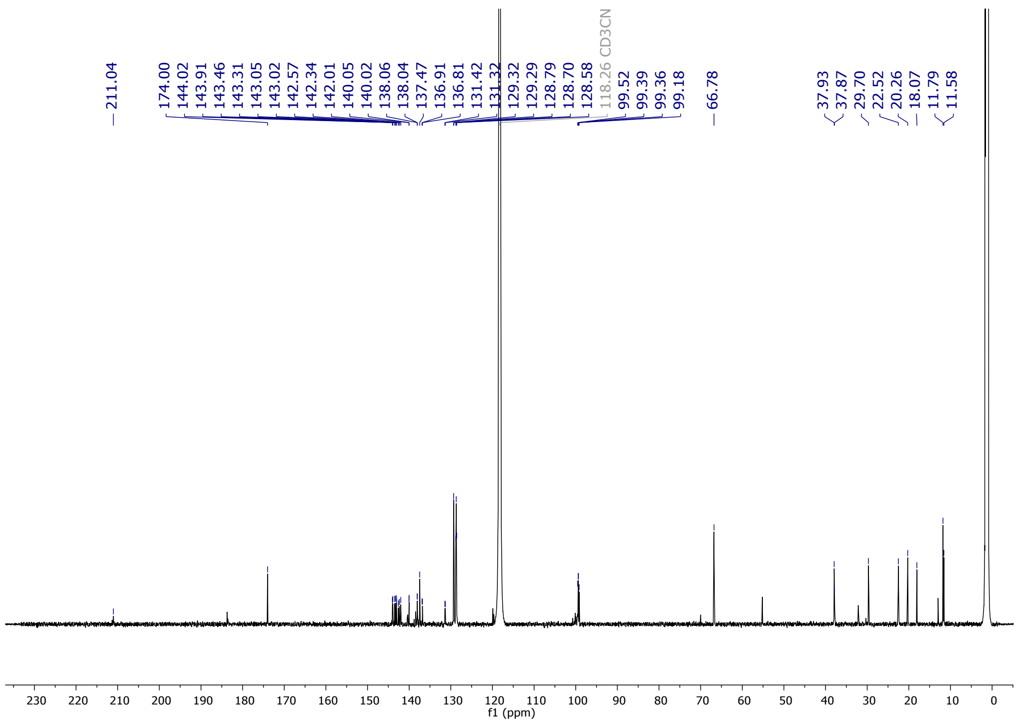

Figure S51. Analytic HPLC of **Ru-2** with detection at 402 nm

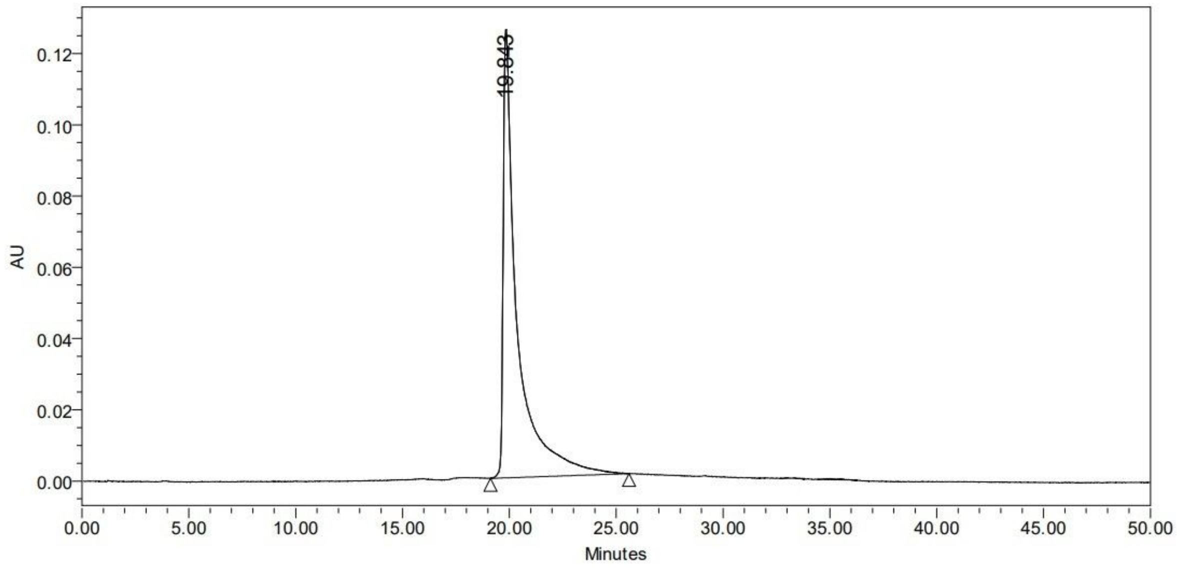

Figure S52. FT-IR spectrum of **Ru-3A**

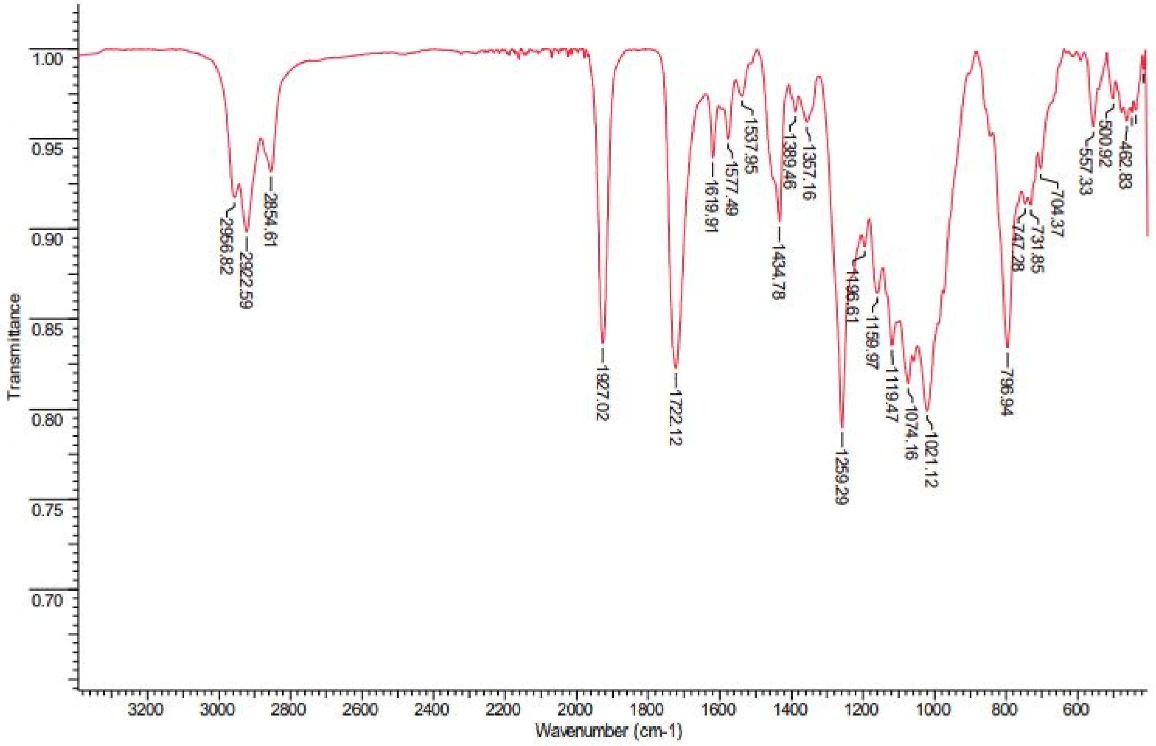

Figure S53. ESI-HRMS spectrum of **Ru-3A**

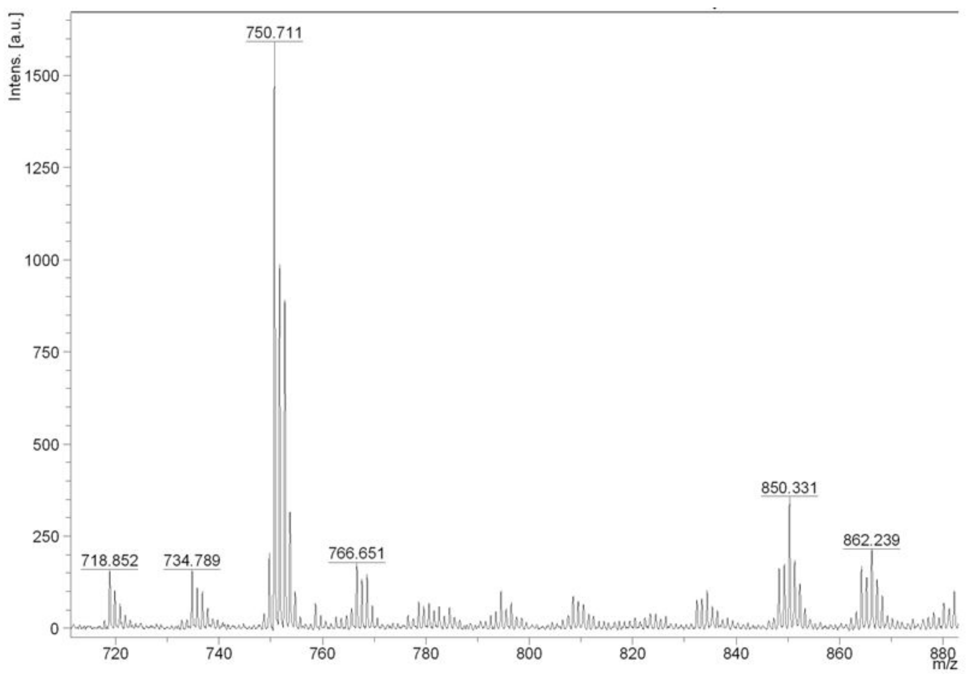

Figure S54. ^{1}H-NMR spectrum of **Ru-3A** in acetone-d_6, 300 MHz

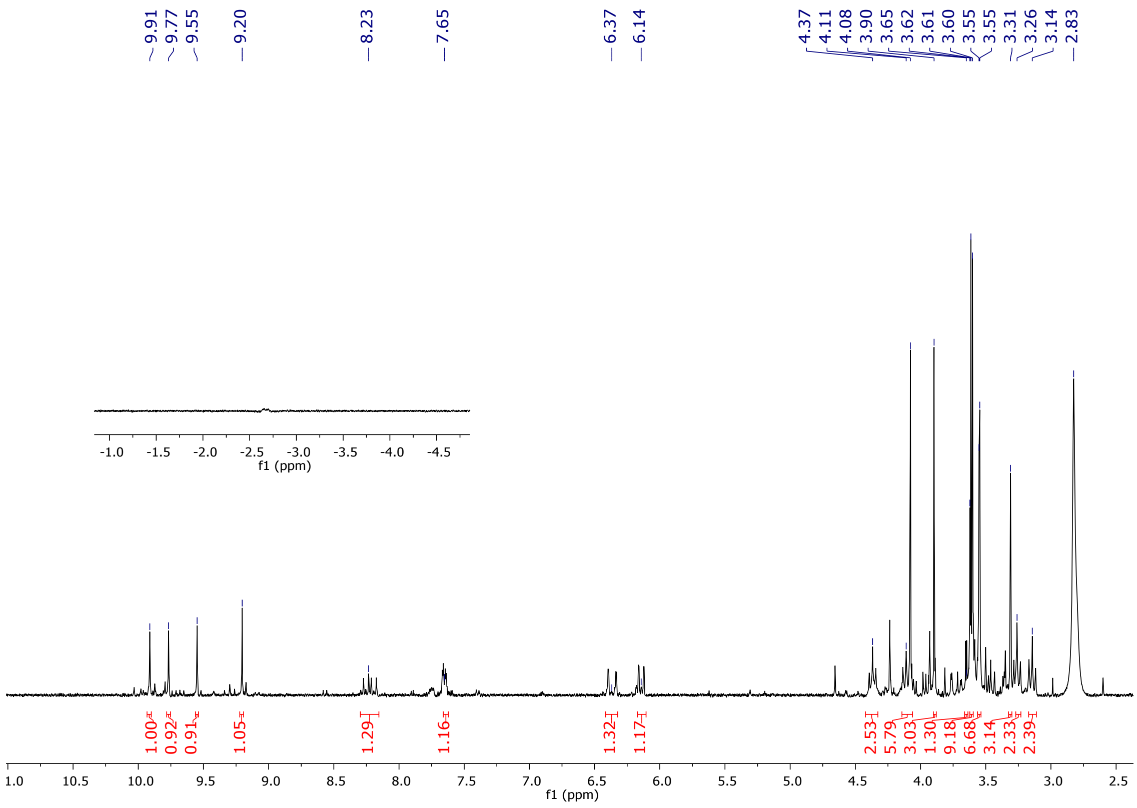

Figure S55. ^{13}C-NMR spectrum of **Ru-3A** in acetone-d_6, 75 MHz

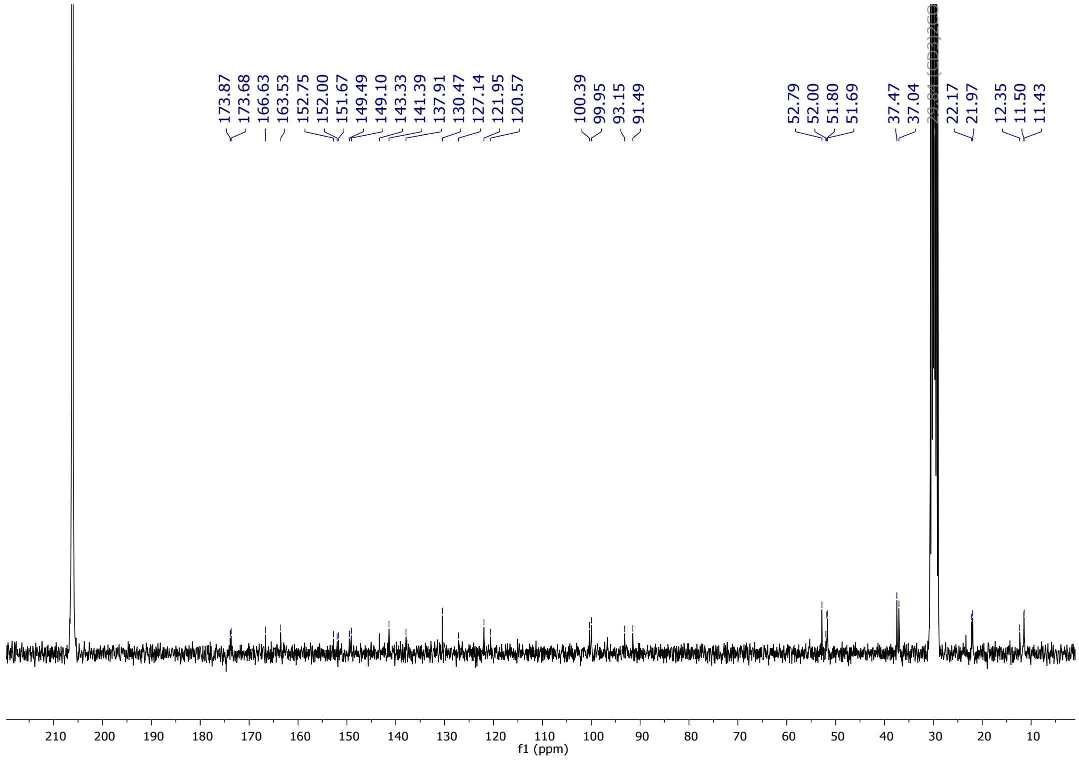

Figure S56. Analytic HPLC of **Ru-3A** with detection at 402 nm

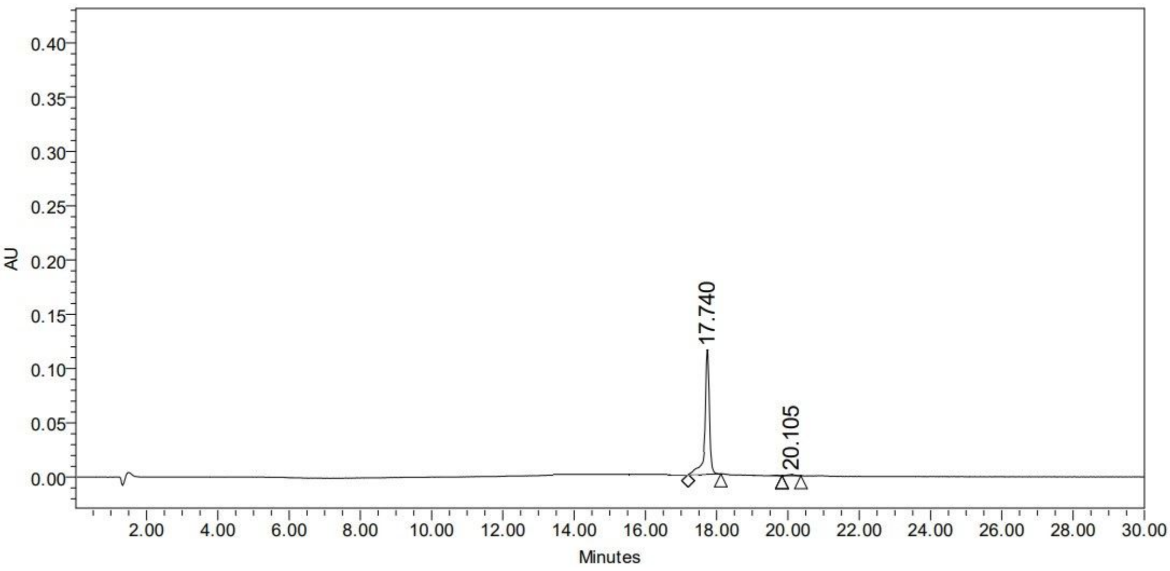

Figure S57. FT-IR spectrum of **Ru-3B**

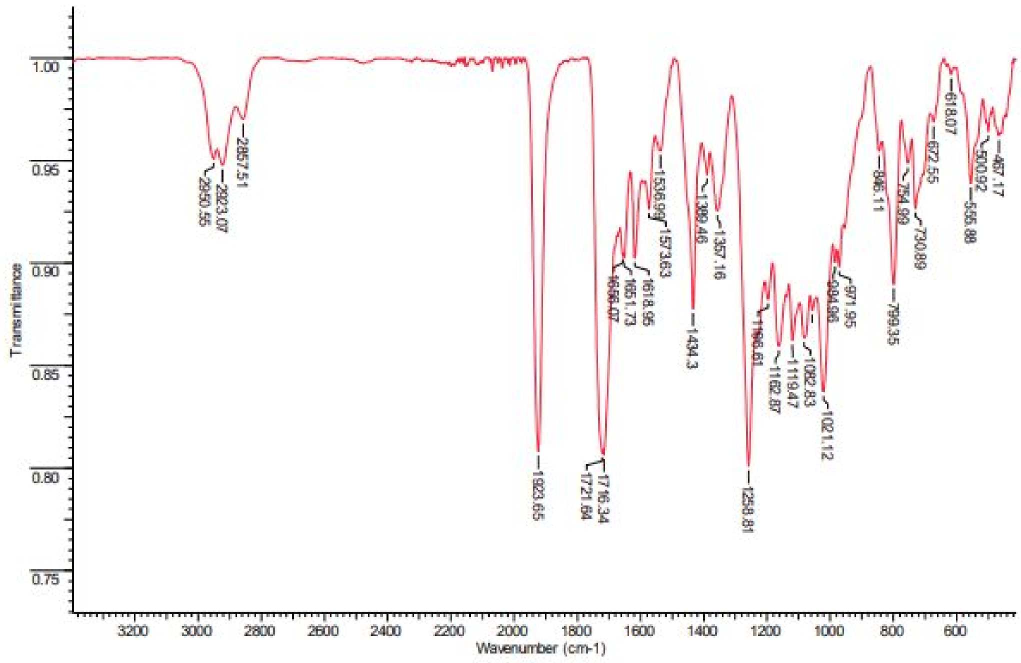

Figure S58. ESI-HRMS spectrum of **Ru-3B** (positive detection mode)

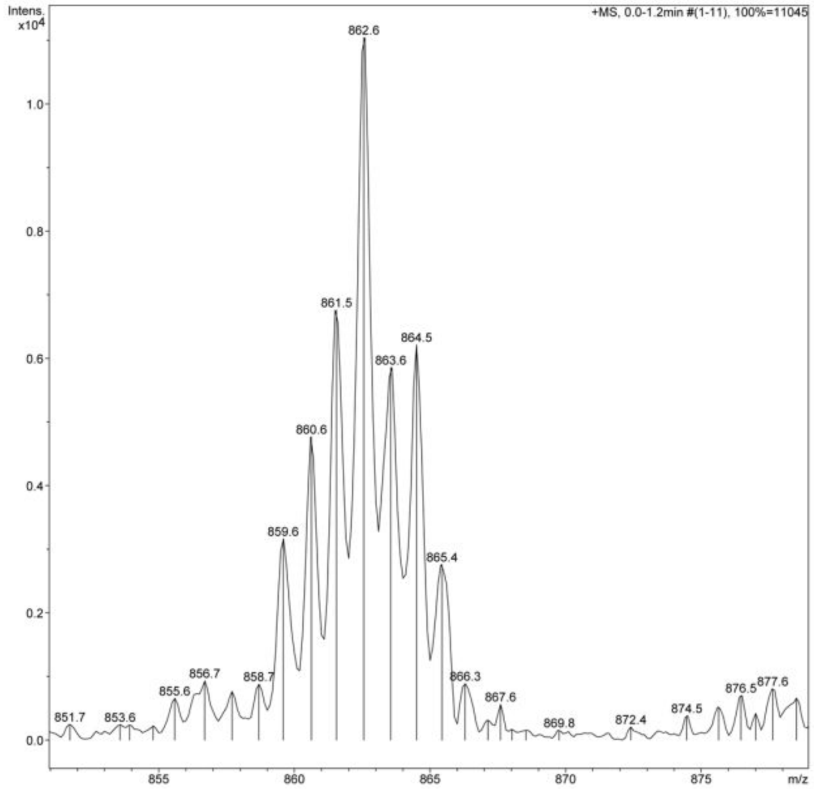

Figure S59. ^{1}H-NMR spectrum of **Ru-3B** in acetone-d_6, 300 MHz

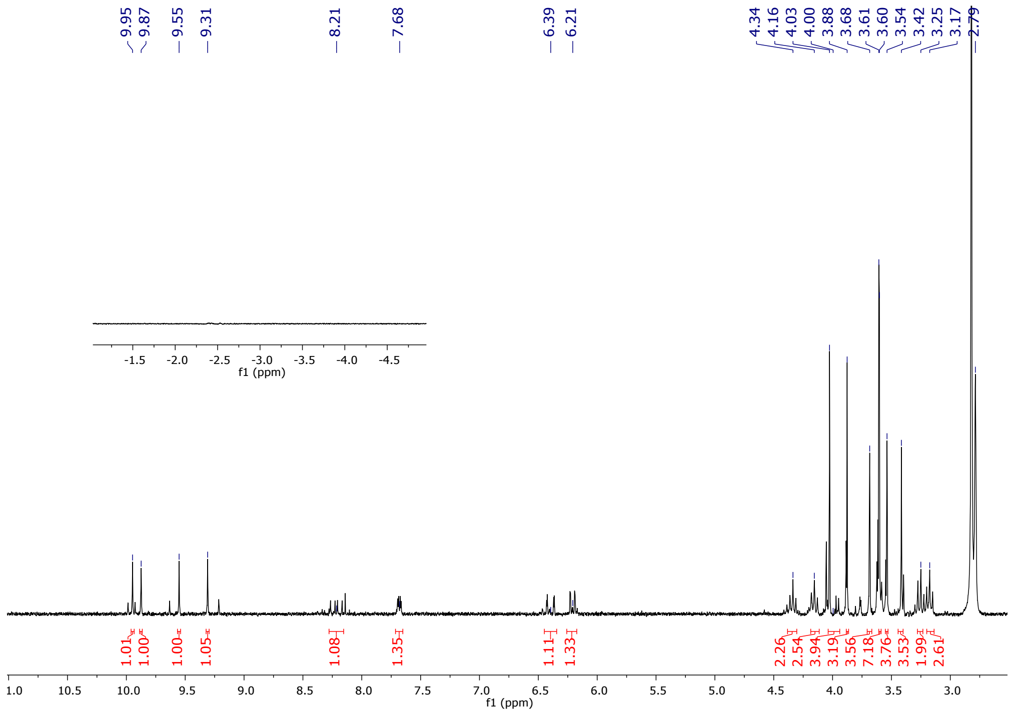

Figure S60. ^{13}C-NMR spectrum of **Ru-3B** in Acetone-d_6, 75 MHz

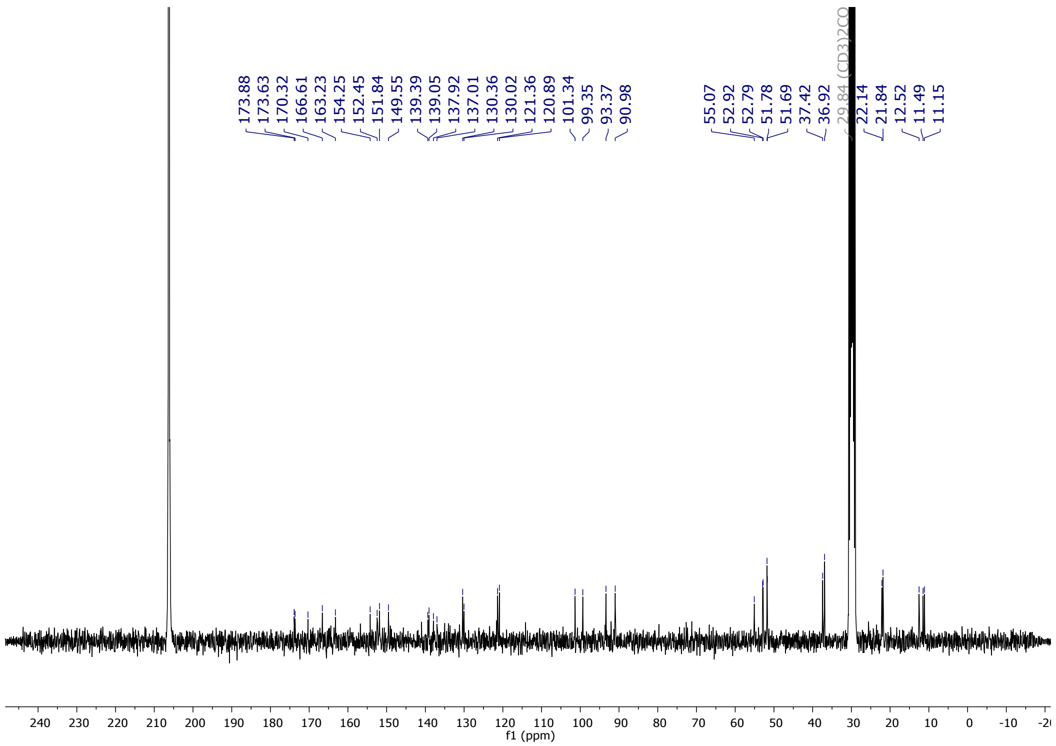

Figure S61. Analytic HPLC of **Ru-3B** with detection at 402 nm

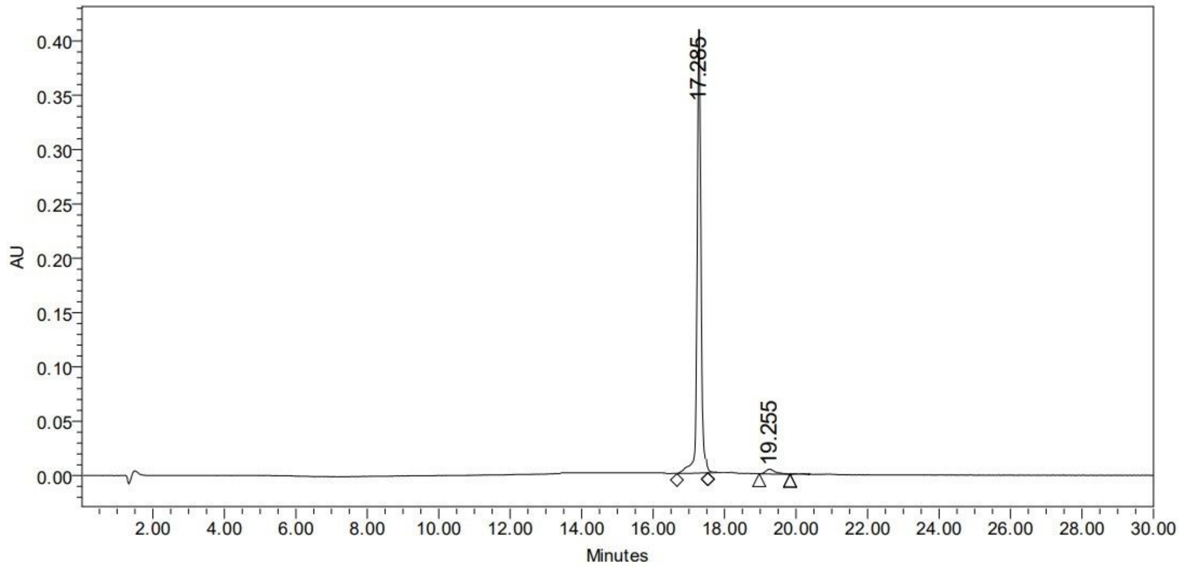

Figure S62. Stability test of **1** in DMSO at 37 °C and 1×10^{-5} M

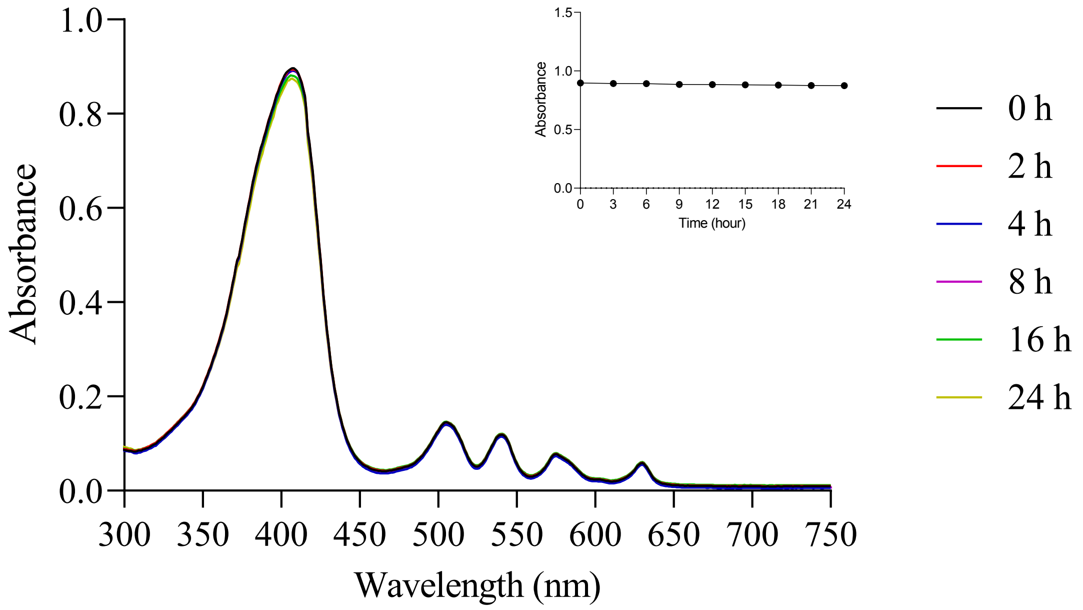

Figure S63. Stability test of **2** in DMSO at 37 °C and 1×10^{-5} M

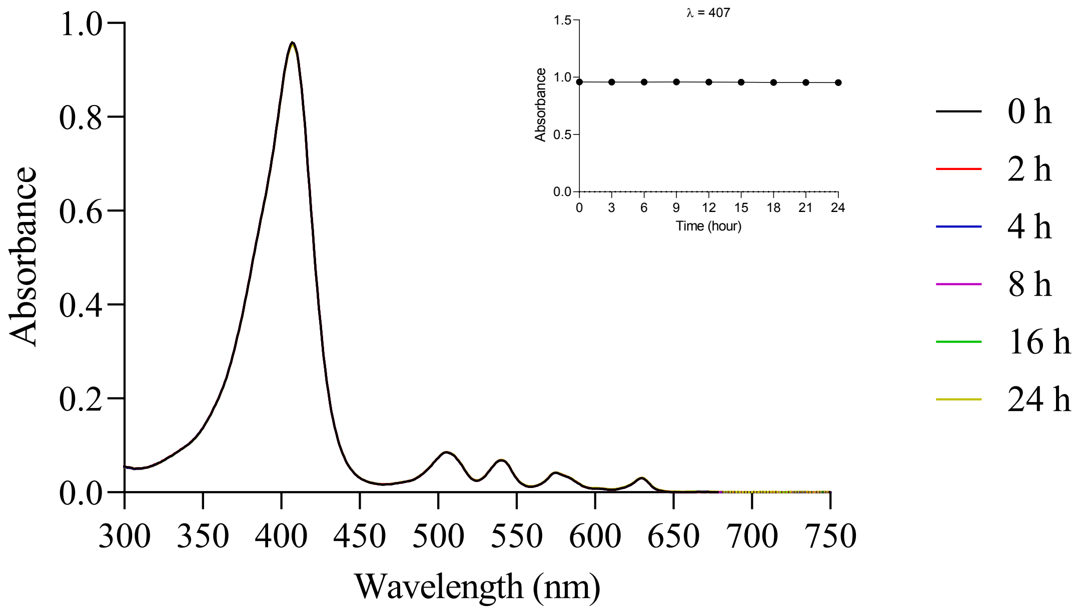

Figure S64. Stability test of **Ru-2** in DMSO at 37 °C and 1×10^{-5} M

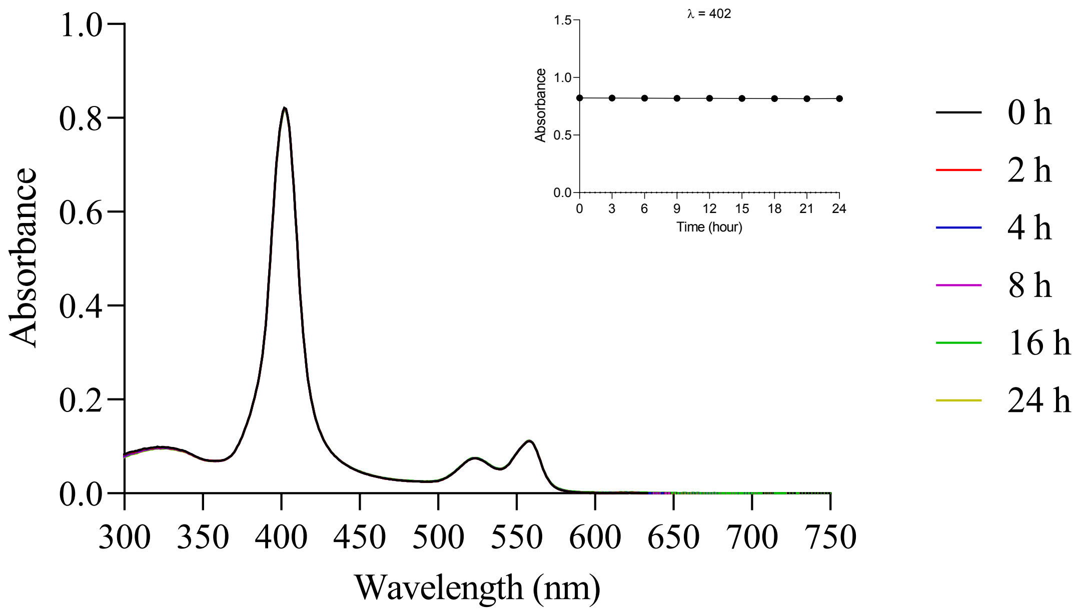

Figure S65. Stability test of **3A** in DMSO at 37 °C and 1×10^{-5} M

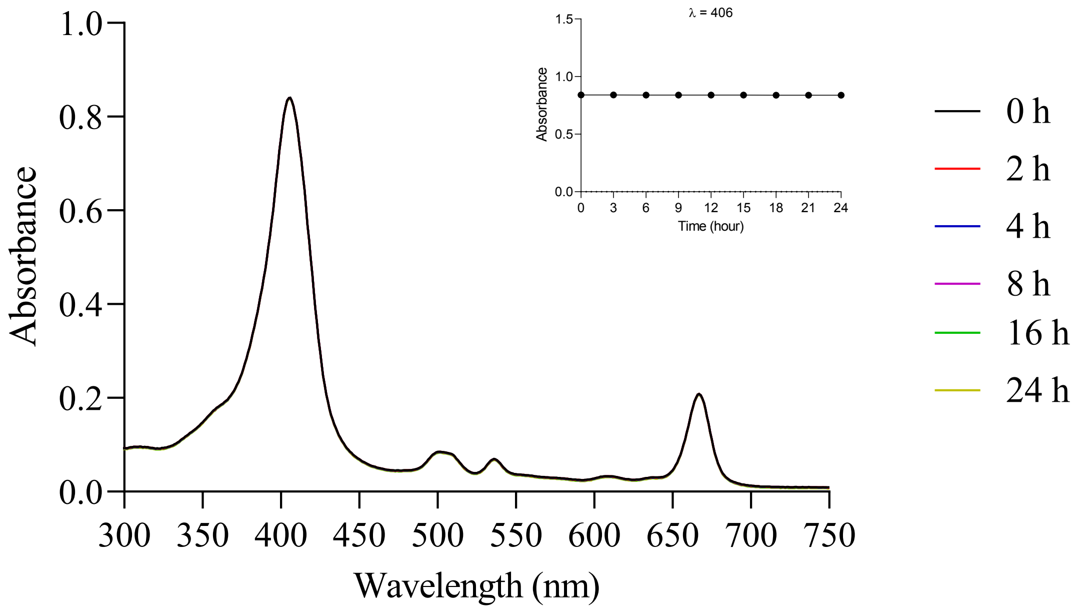

Figure S66. Stability test of **Ru-3A** in DMSO at 37 °C and 1×10^{-5} M

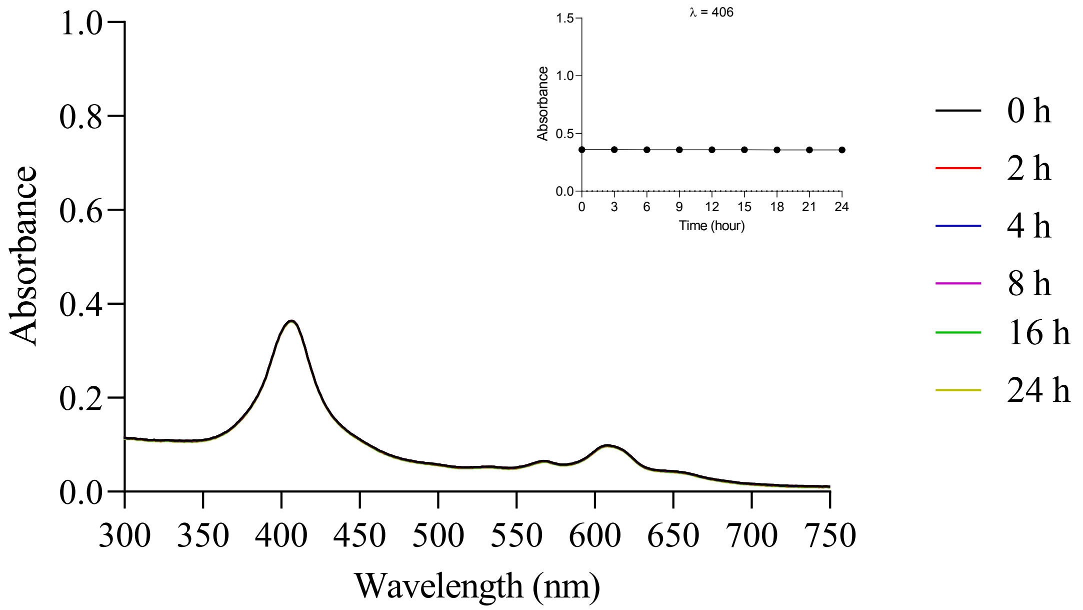

Figure S67. Stability test of **3B** in DMSO at 37 °C and 1×10^{-5} M

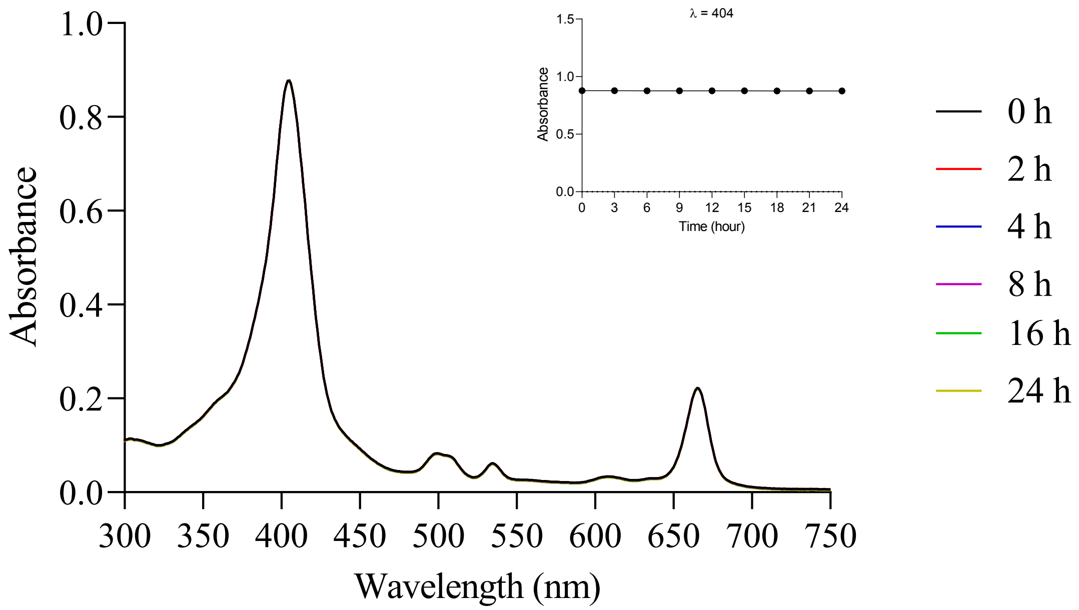

Figure S68. Stability test of **Ru-3B** in DMSO at 37 °C and 1×10^{-5} M

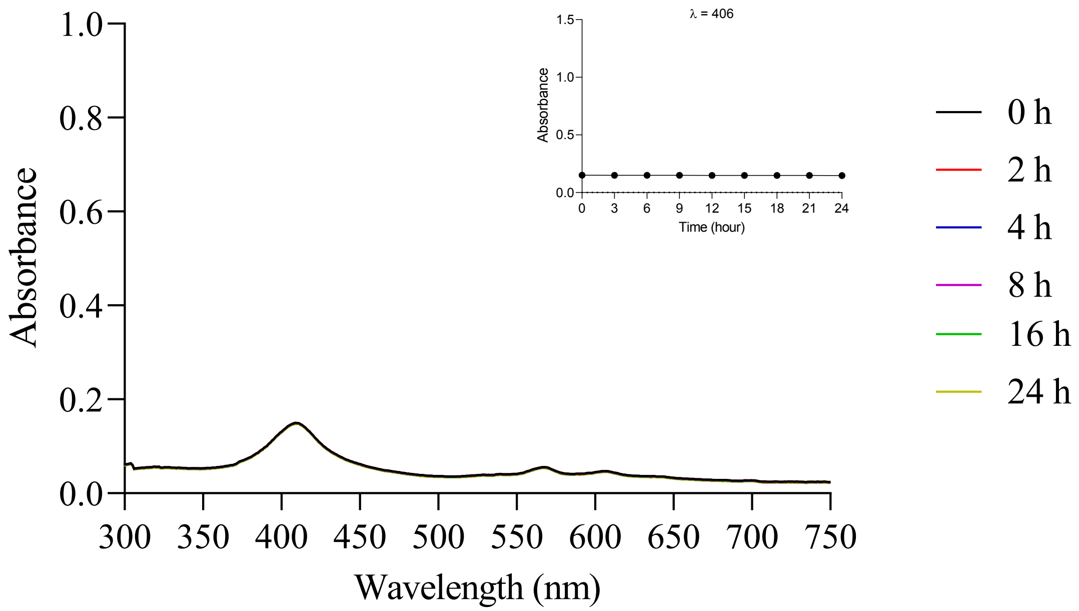

Figure S69. Stability test of **4A** in DMSO at 37 °C and 1×10^{-5} M

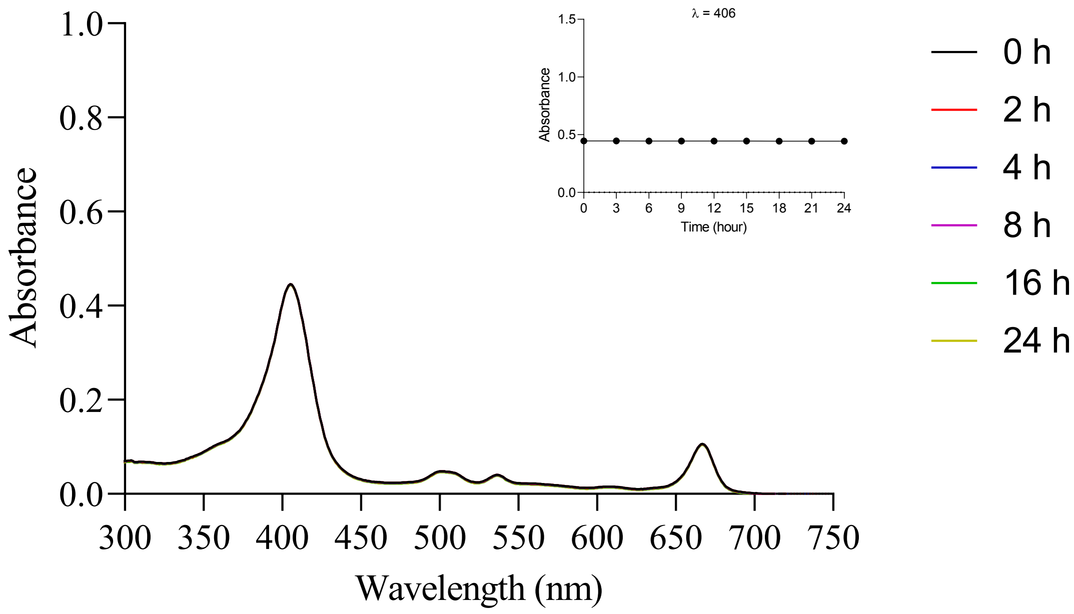

Figure S70. Stability test of **4B** in DMSO at 37 °C and 1×10^{-5} M

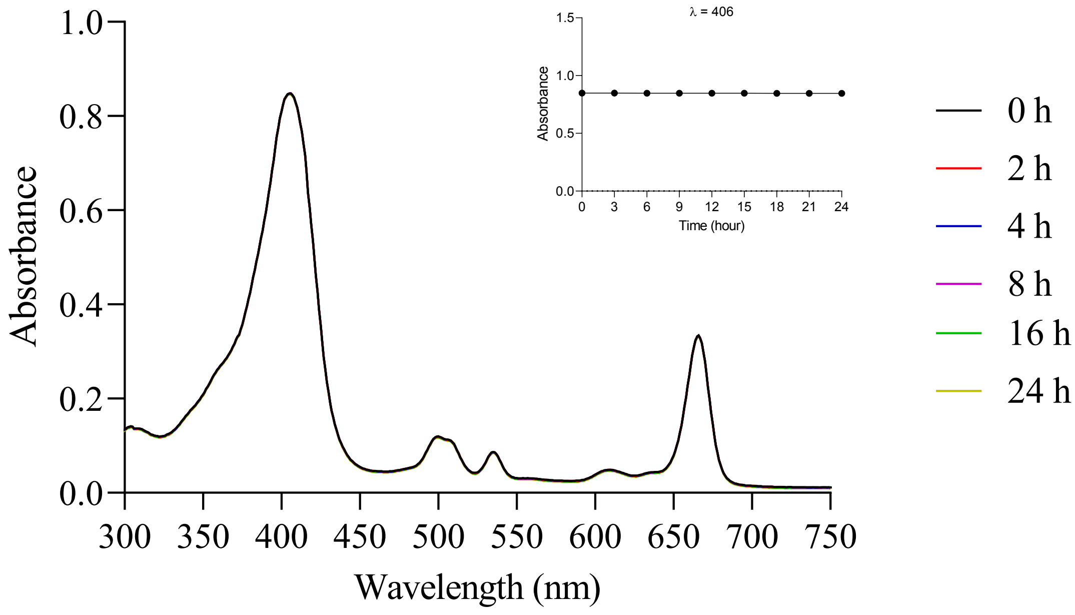

Figure S71. Stability test of **1** in PBS/DMSO(0.1%) at 37 °C and 1×10^{-5} M

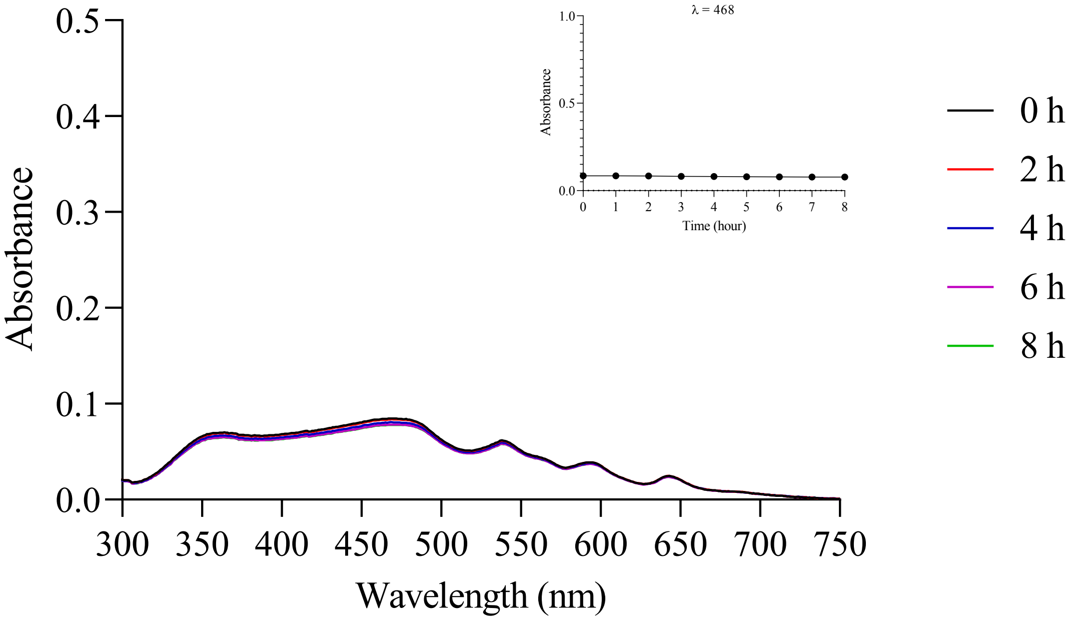

Figure S72. Stability test of **2** in PBS/DMSO(0.1%) at 37 °C and 1×10^{-5} M

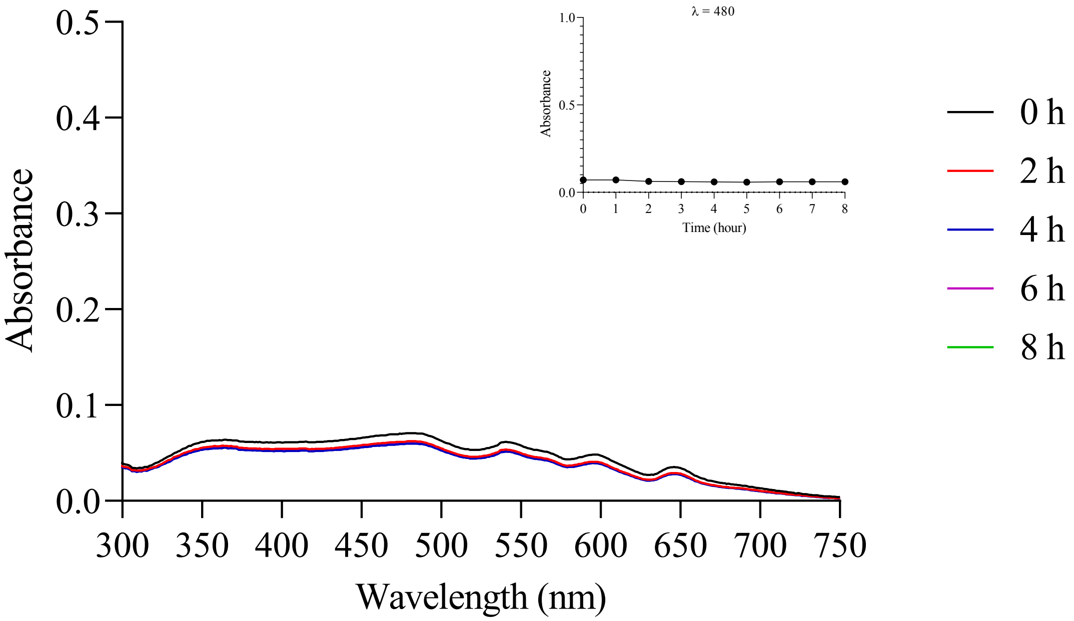

Figure S73. Stability test of **Ru-2** in PBS/DMSO(0.1%) at 37 °C and 1×10^{-5} M

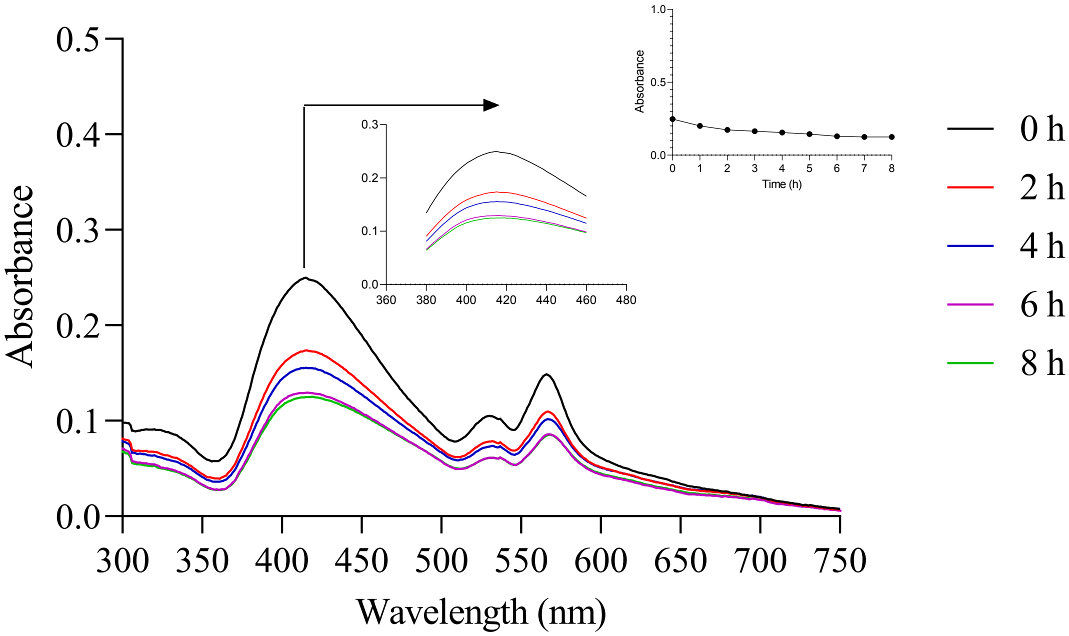

Figure S74. Stability test of **3A** in PBS/DMSO(0.1%) at 37 °C and 1×10^{-5} M

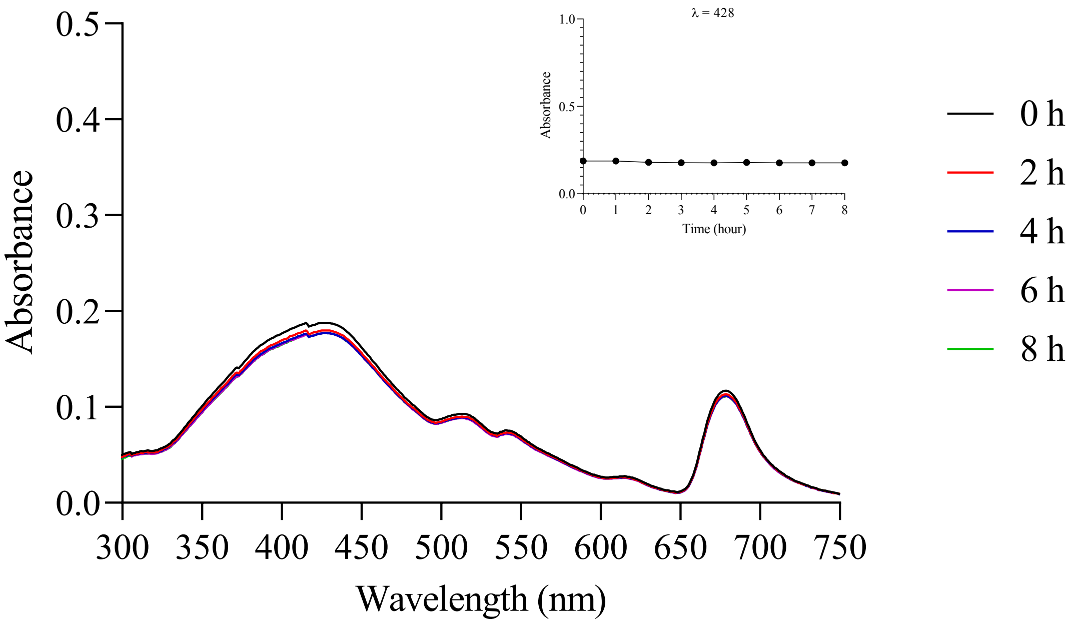

Figure S75. Stability test of **Ru-3A** in PBS/DMSO(0.1%) at 37 °C and 1×10^{-5} M

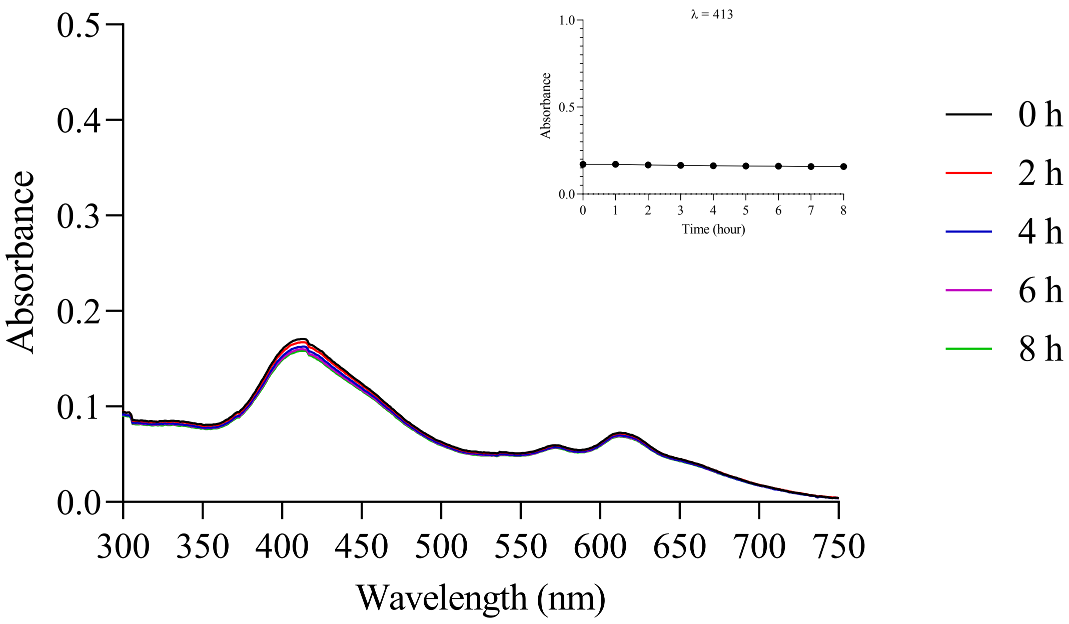

Figure S76. Stability test of **3B** in PBS/DMSO(0.1%) at 37 °C and 1×10^{-5} M

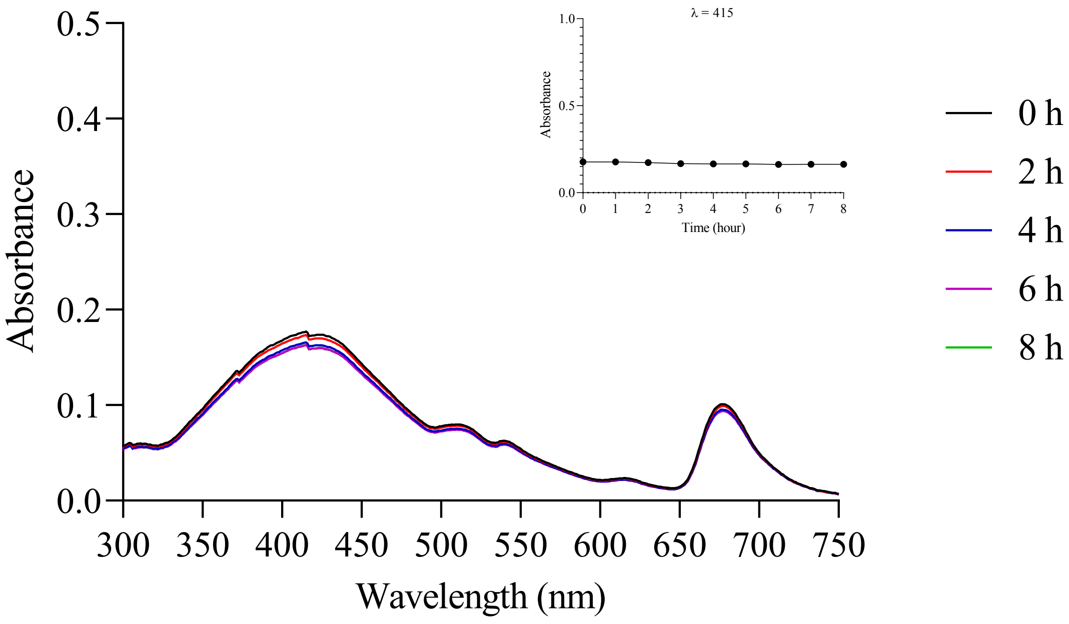

Figure S77. Stability test of **Ru-3B** in PBS/DMSO(0.1%) at 37 °C and 1×10^{-5} M

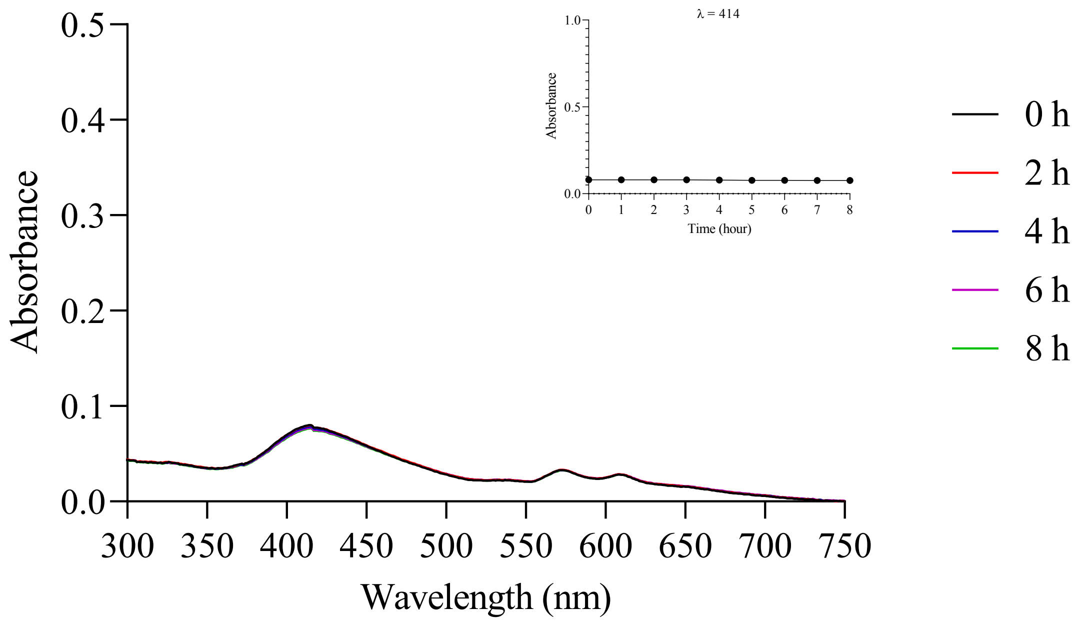

Figure S78. Stability test of **4A** in PBS/DMSO(0.1%) at 37 °C and 1×10^{-5} M

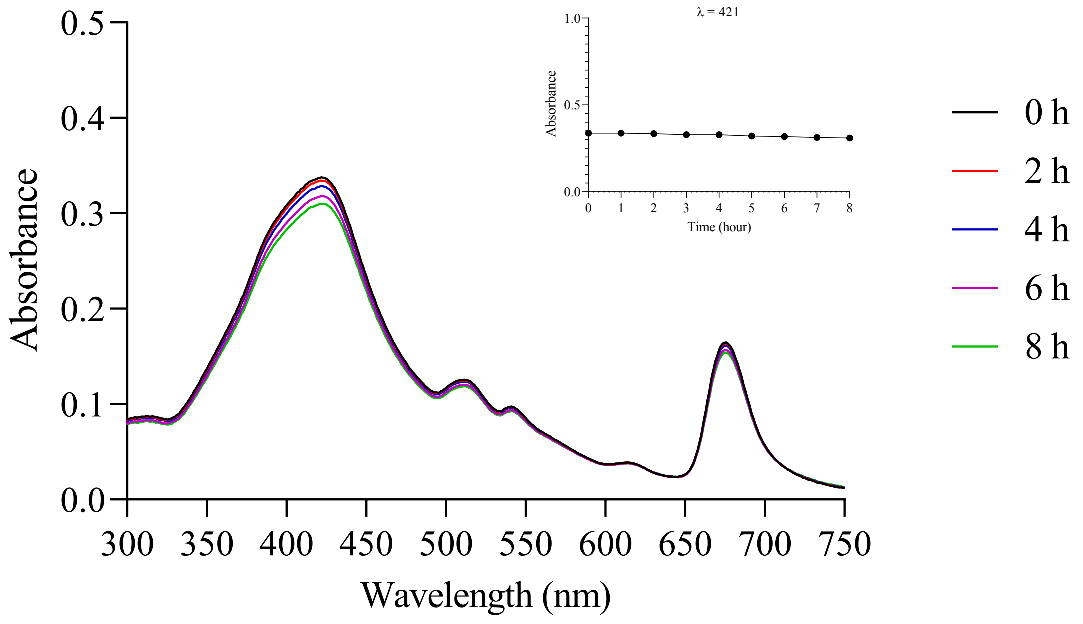

Figure S79. Stability test of **4B** in PBS/DMSO(0.1%) at 37 °C and 1×10^{-5} M

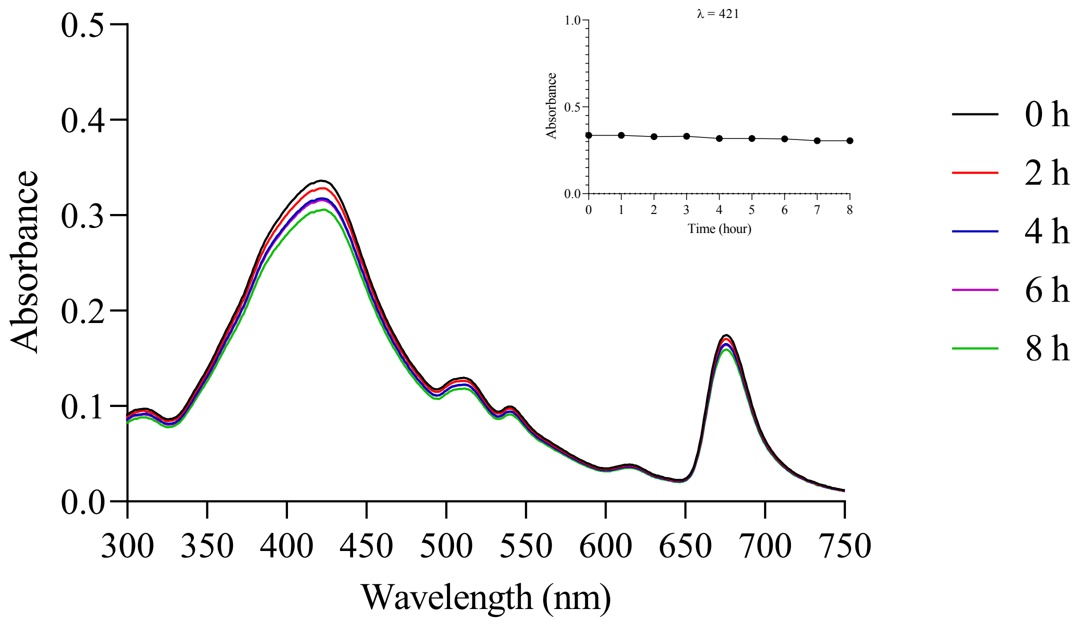

Figure S80. Photodegradation of compound **1** in DMSO using white light

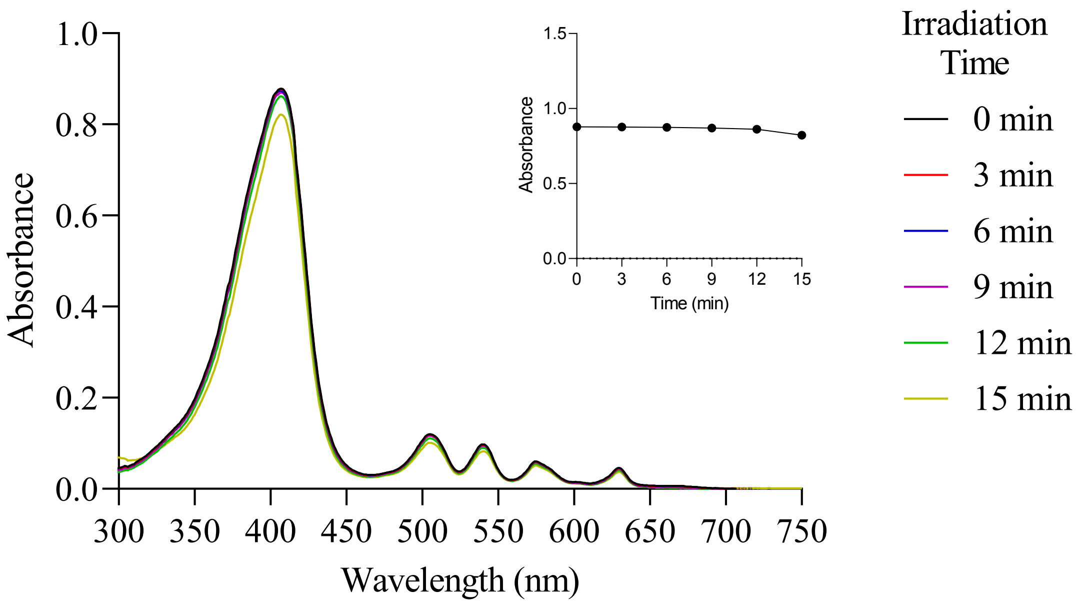

Figure S81. Photodegradation of compound **2** in DMSO using white light

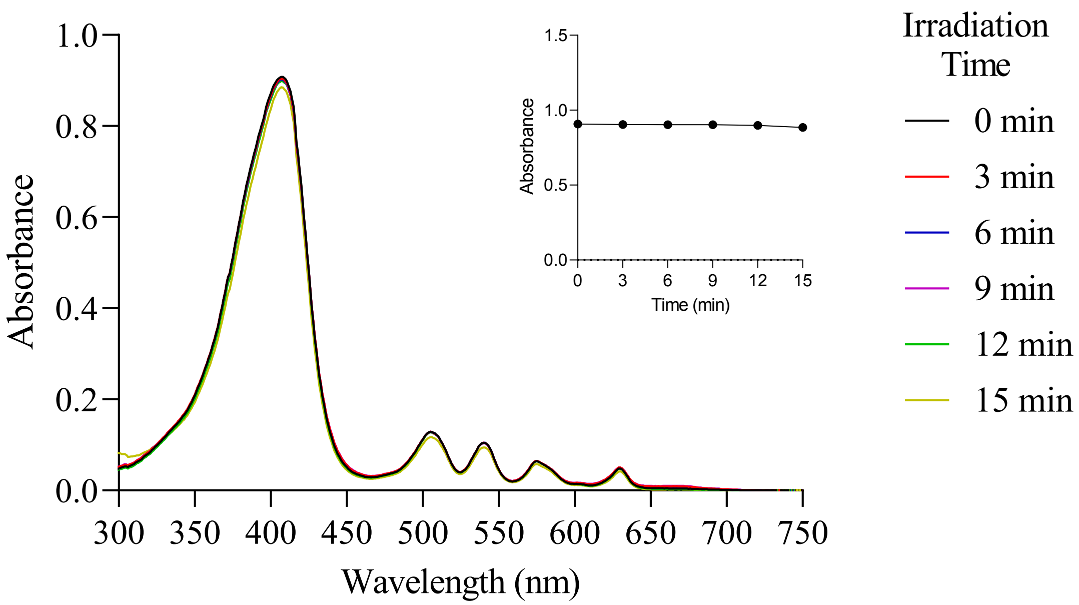

Figure 82. Photodegradation of compound **Ru-2** in DMSO using white light

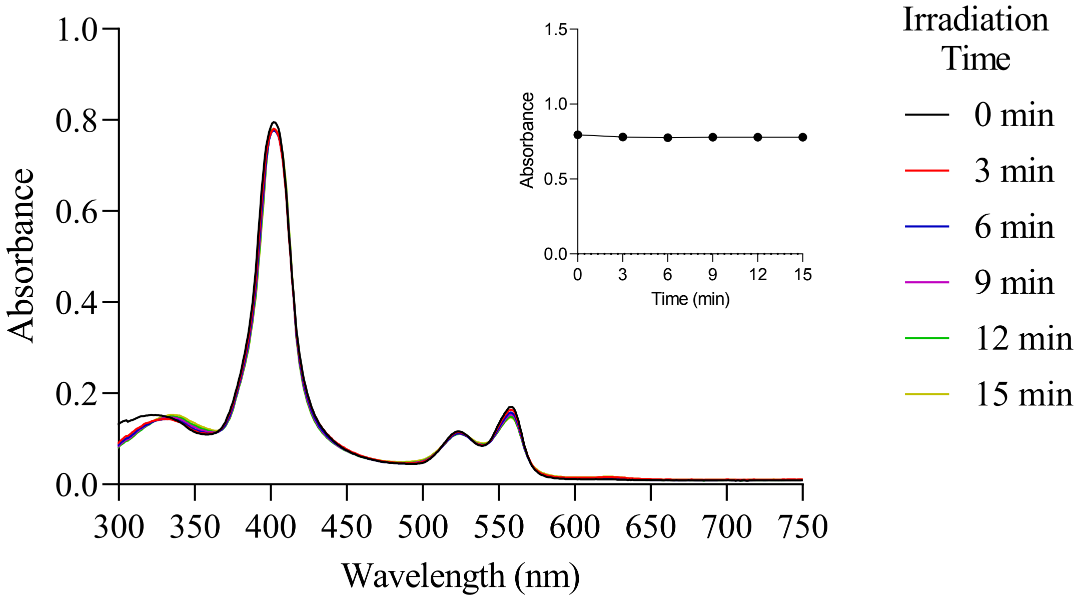

Figure S83. Photodegradation of compound **3A** in DMSO using white light

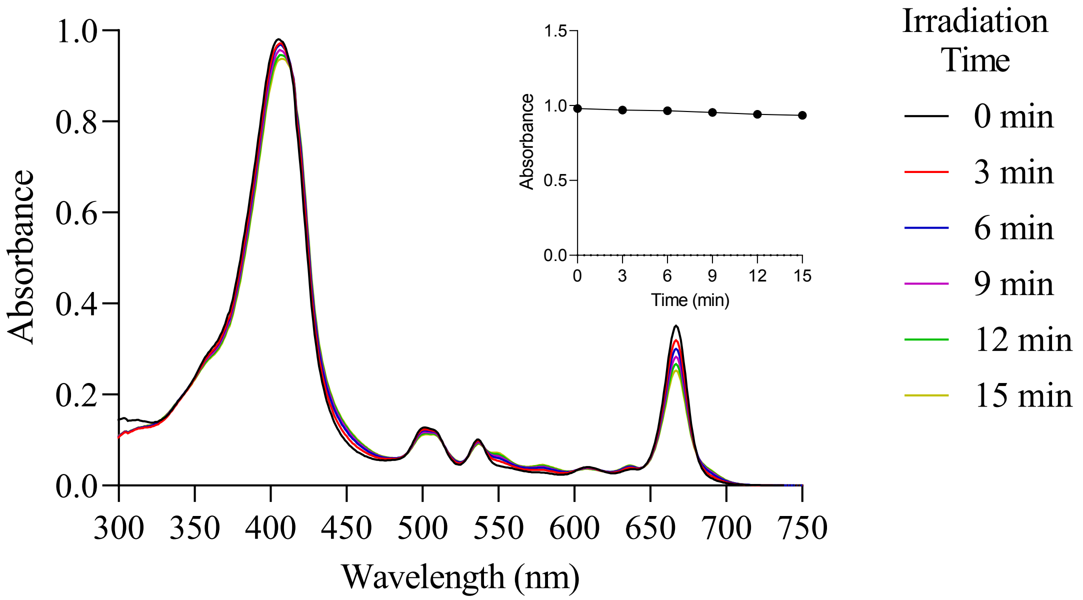

Figure S84. Photodegradation of compound **Ru-3A** in DMSO using white light

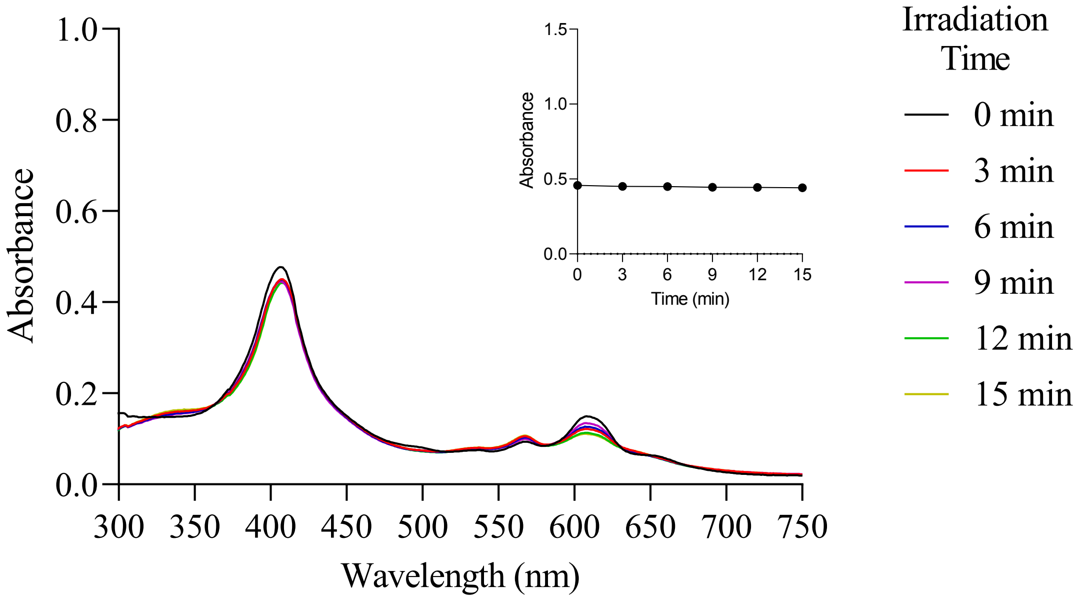

Figure S85. Photodegradation of compound **3B** in DMSO using white light

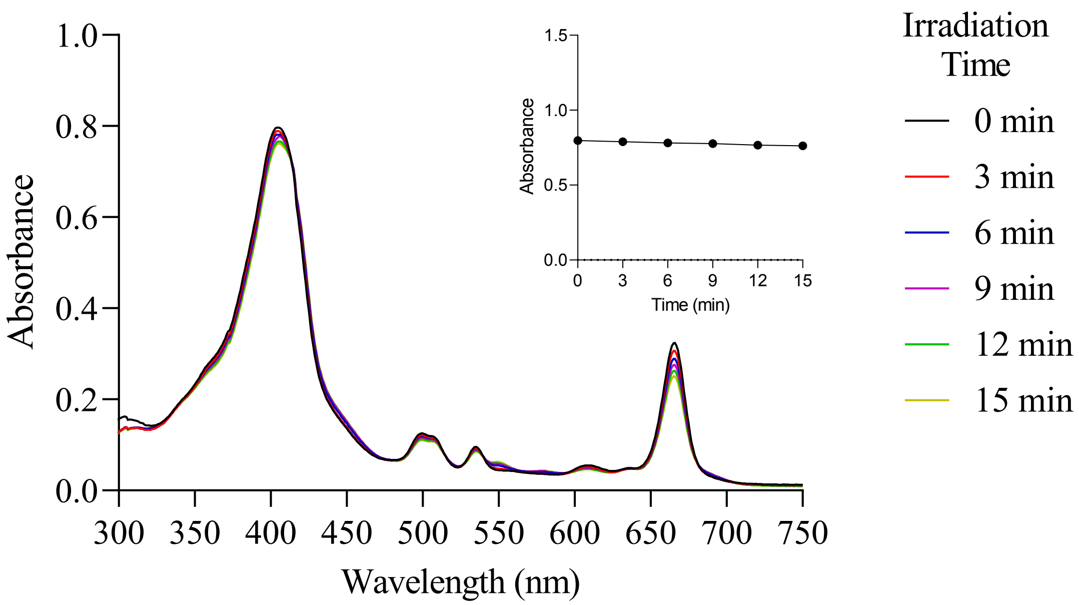

Figure S86. Photodegradation of compound **4A** in DMSO using white light

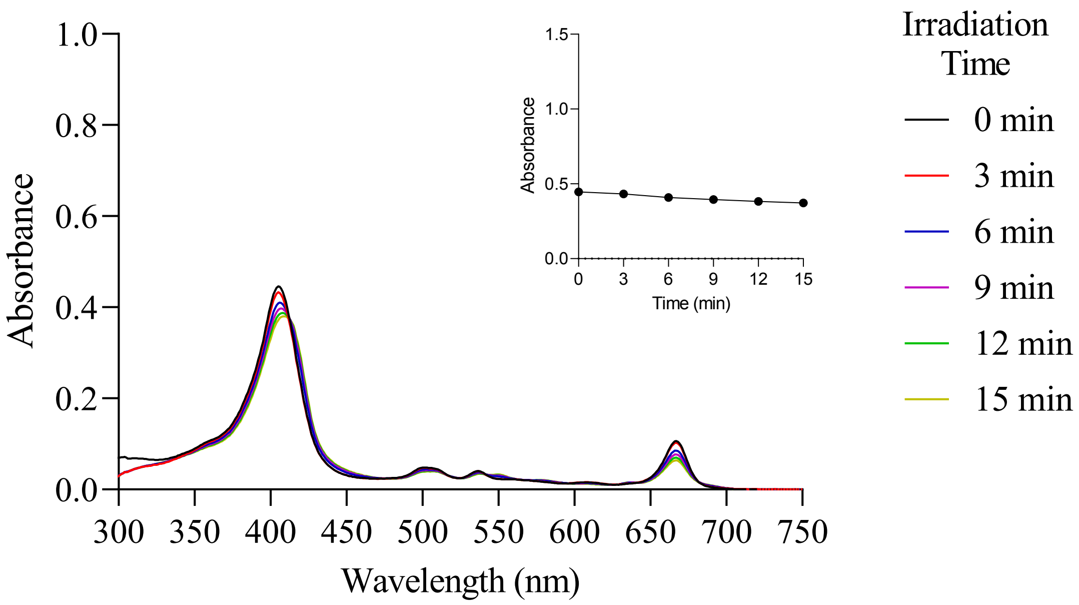

Figure S87. Photodegradation of compound **4B** in DMSO using white light

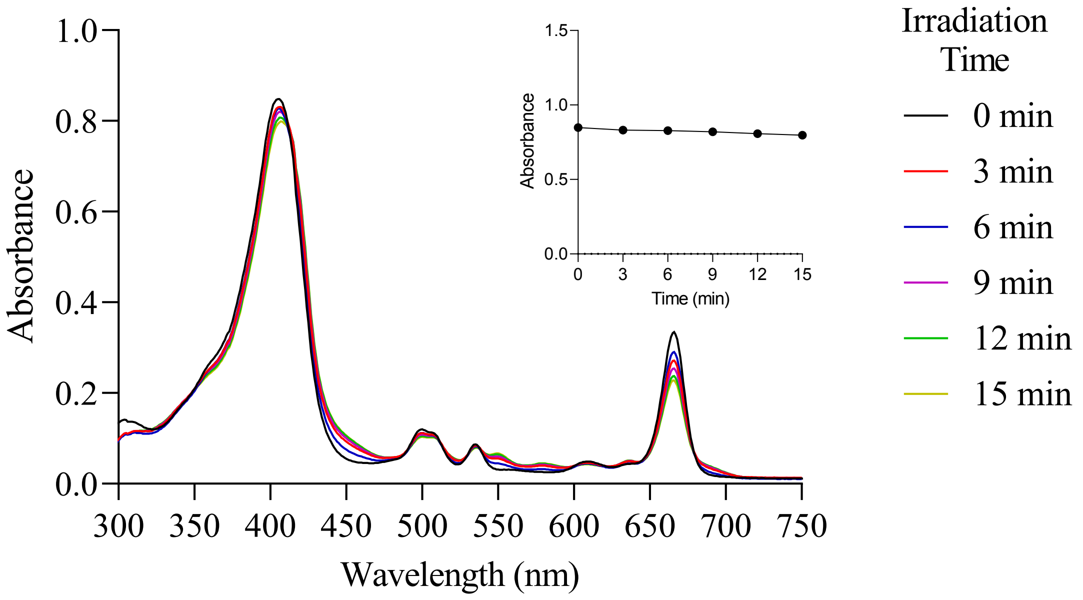

Figure S88. Absorption and emission spectra in DMSO for **1** excitation at 503 nm

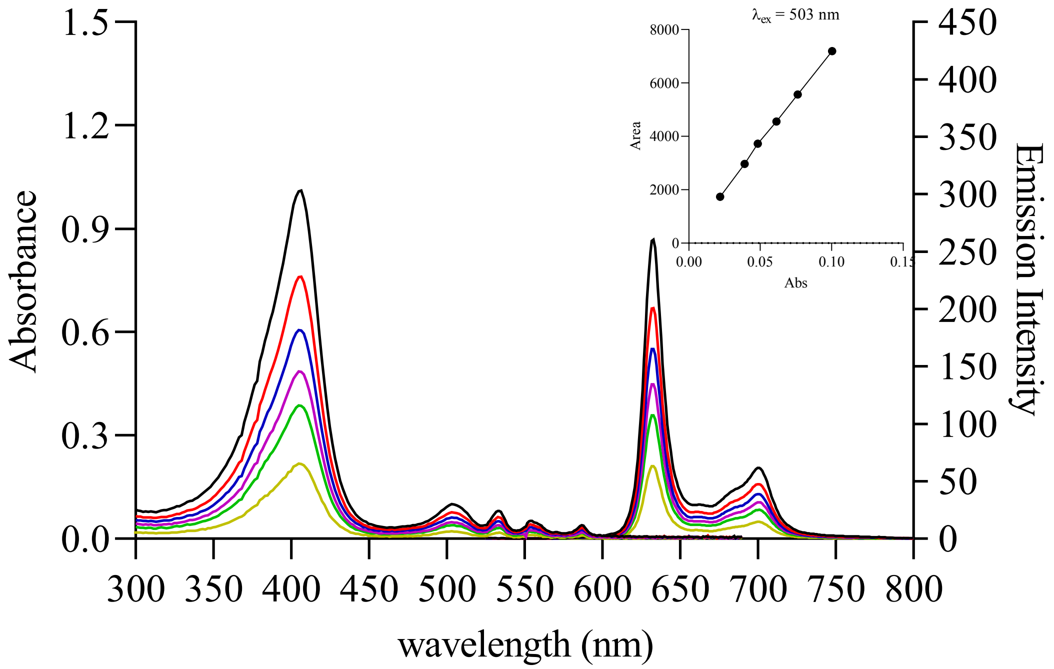

Figure S89. Absorption and emission spectra in DMSO for **2** excitation at 503 nm

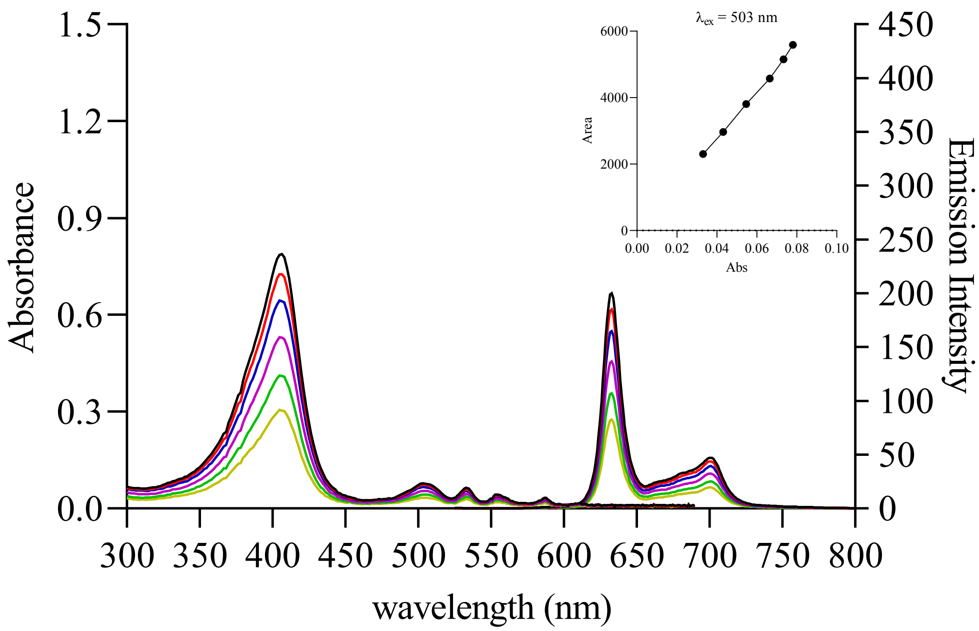

Figure S90. Absorption and emission spectra in DMSO for **3A** excitation at 503 nm

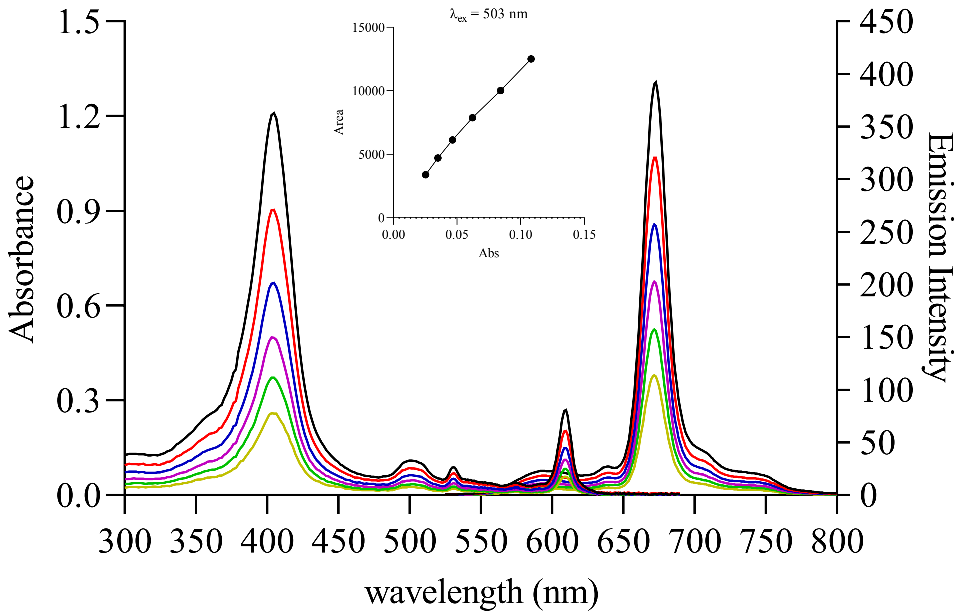

Figure S91. Absorption and emission spectra in DMSO for **3B** excitation at 503 nm

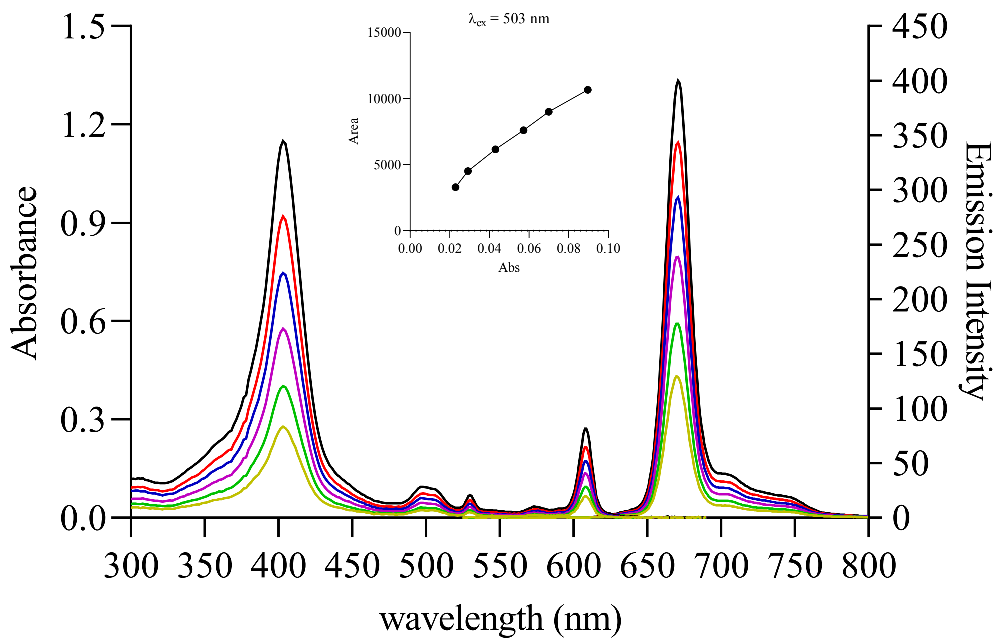

Figure S92. Absorption and emission spectra in DMSO for **4A** excitation at 503 nm

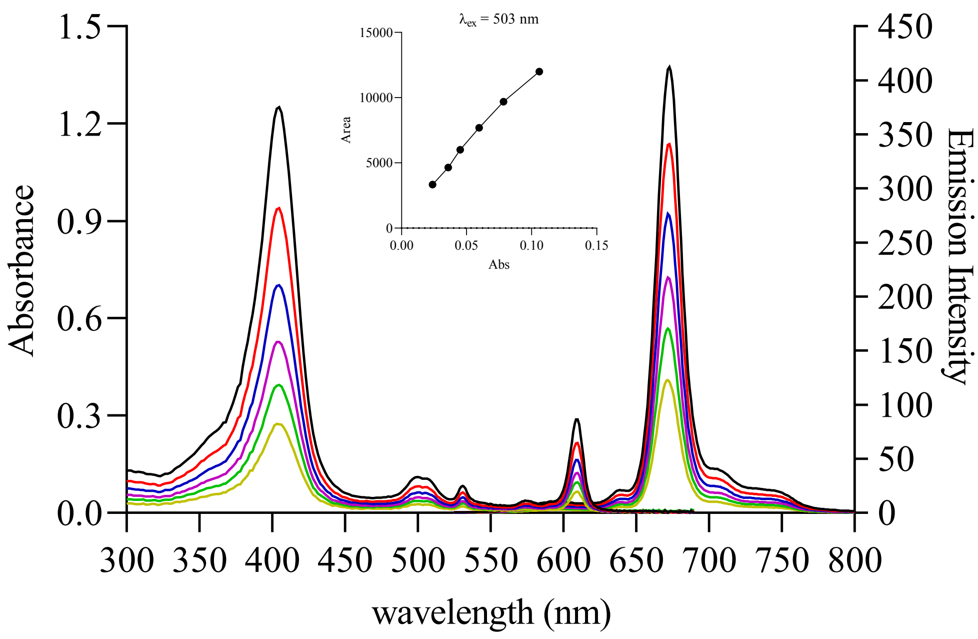

Figure S93. Absorption and emission spectra in DMSO for **4B** excitation at 503 nm

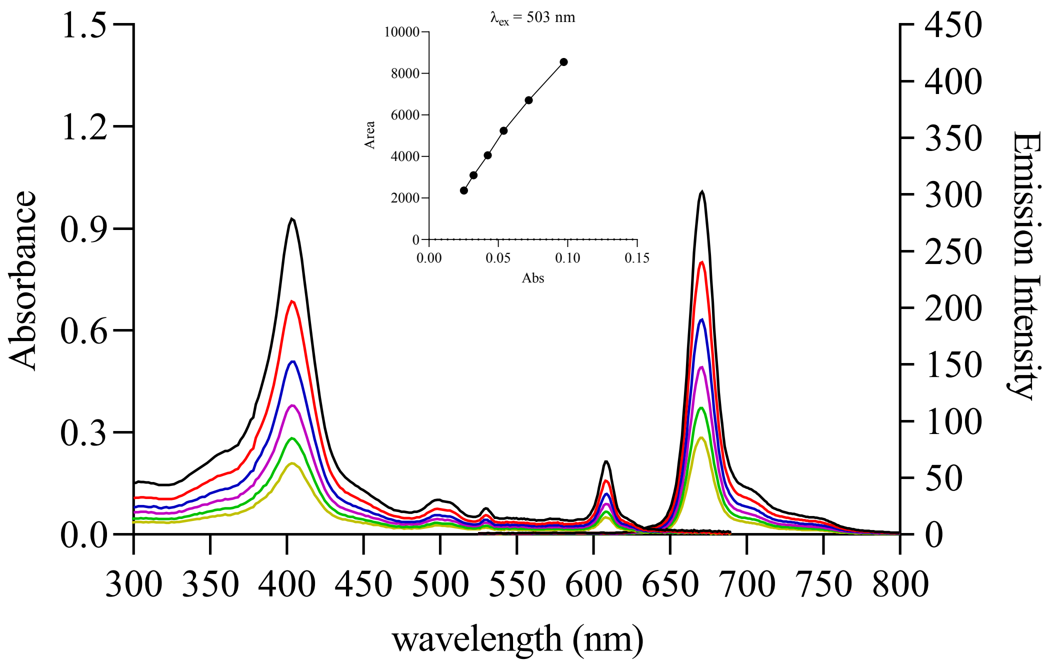

Figure S94. EPR spectra upon irradiation for 5 and 15 min of **1** in the presence of TEMP varying the oxygen concentration: (a) 5 % of oxygen, (b) 45 % of oxygen, (c) 96 % of oxygen.

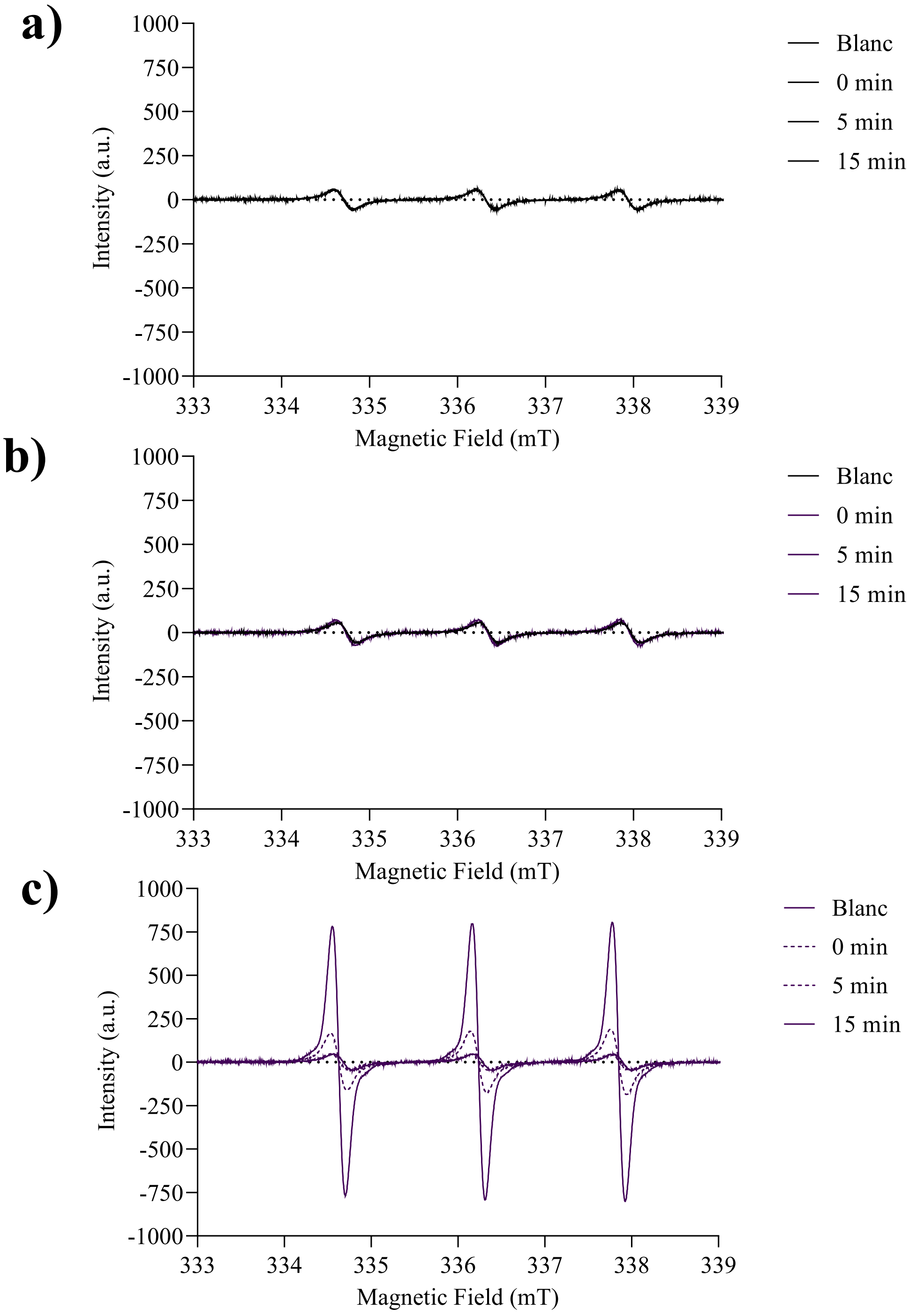

Figure S95. EPR spectra upon irradiation for 5 and 15 min of **3B** in the presence of TEMP varying the oxygen concentration: (a) 5 % of oxygen, (b) 45 % of oxygen, (c) 96 % of oxygen.

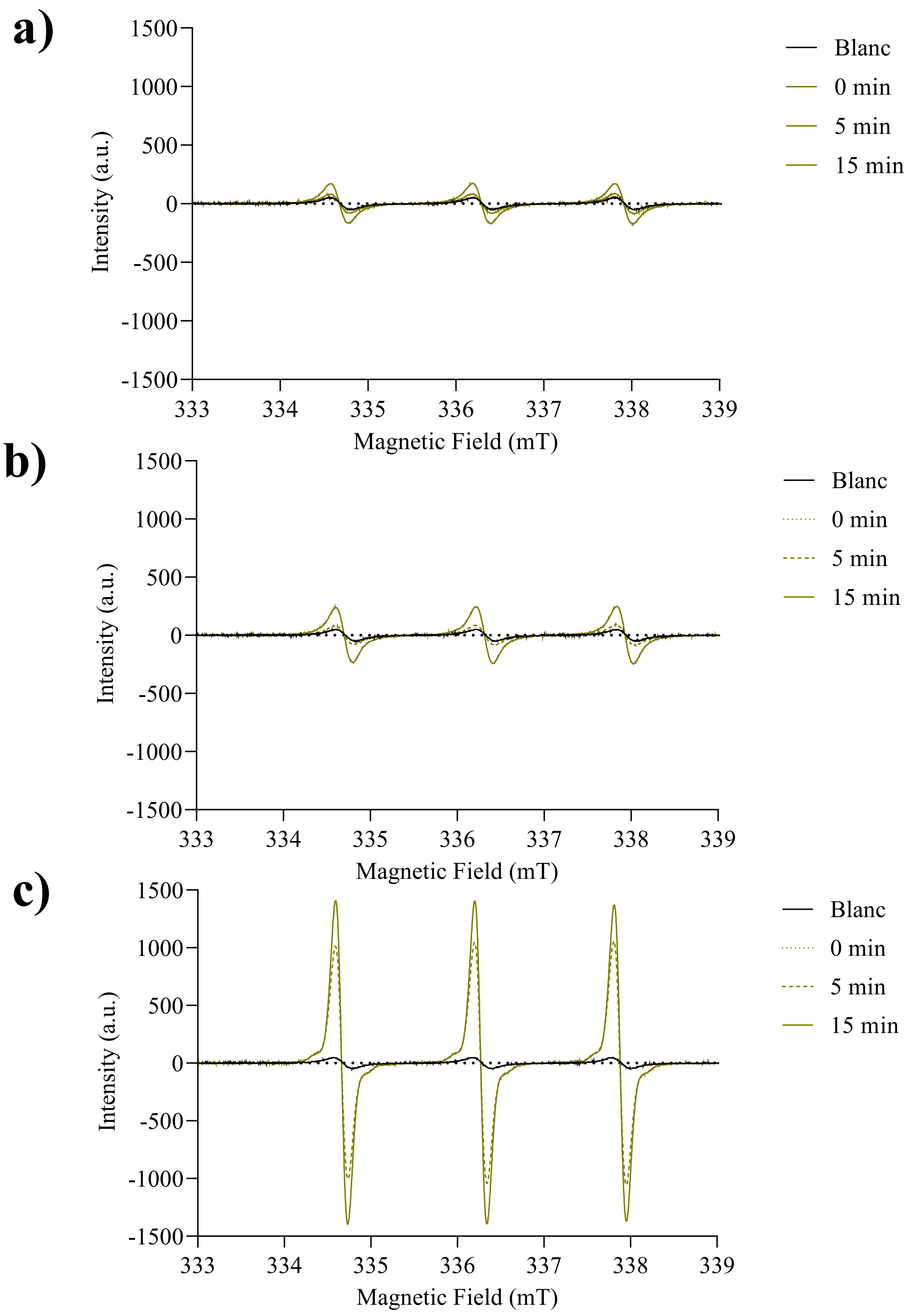

Figure S96. EPR spectra upon irradiation for 5 and 15 min of **Ru-3B** in the presence of TEMP varying the oxygen concentration: (a) 5 % of oxygen, (b) 45 % of oxygen, (c) 96 % of oxygen.

a)

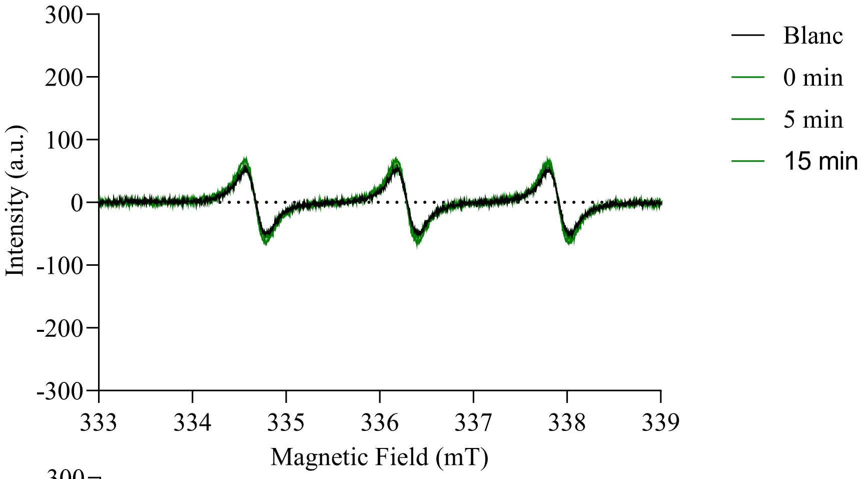

b)

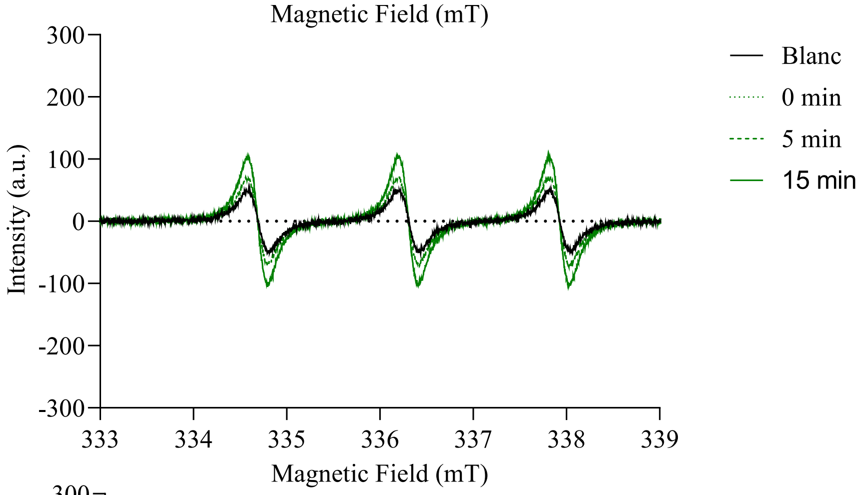

c)

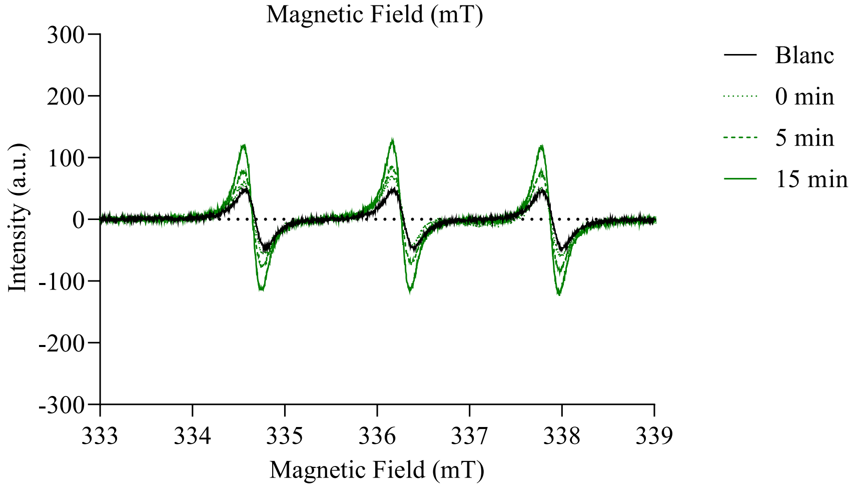

Figure S97. Cell viability curves for the compounds **1** (left) and **Ru-1** (right) in the AGS cell line.

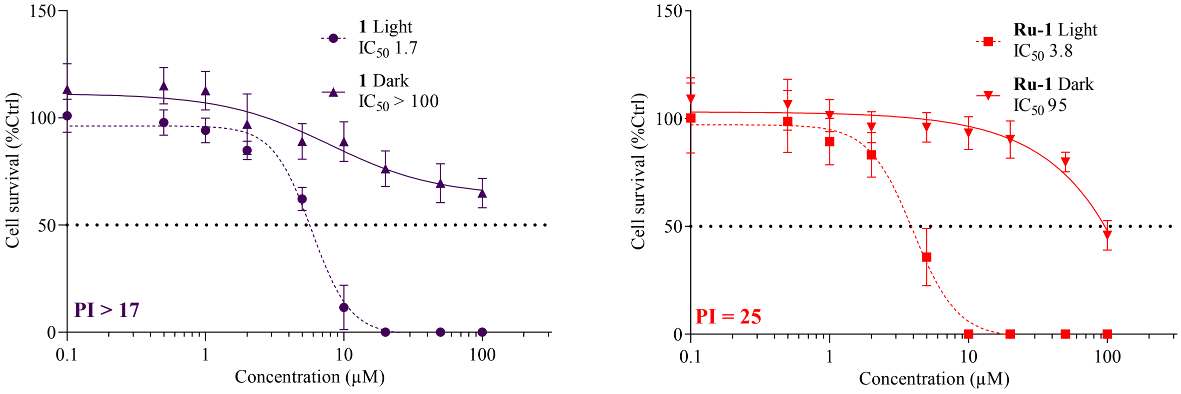

Figure S98. Cell viability curves for the compounds **2** (left) and **Ru-2** (right) in the AGS cell line.

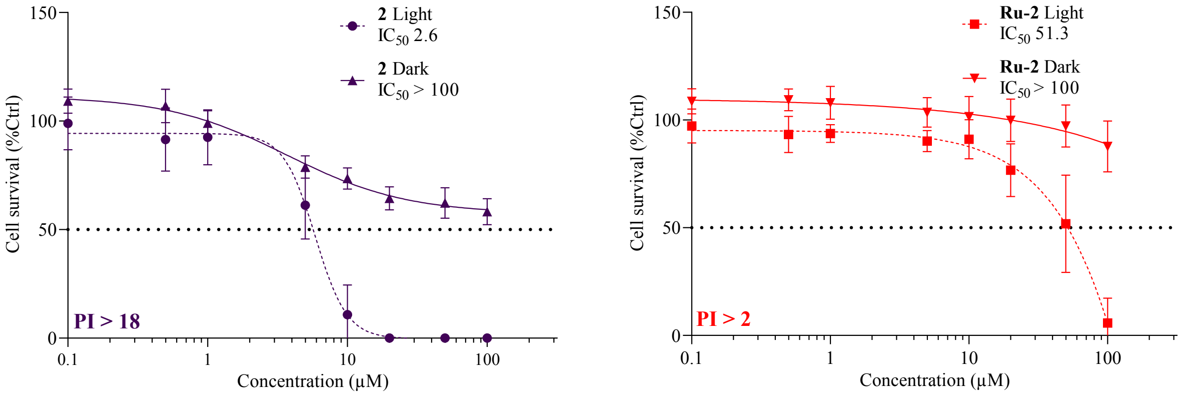

Figure S99. Cell viability curves for the compounds **3A** (left) and **Ru-3A** (right) in the AGS cell line.

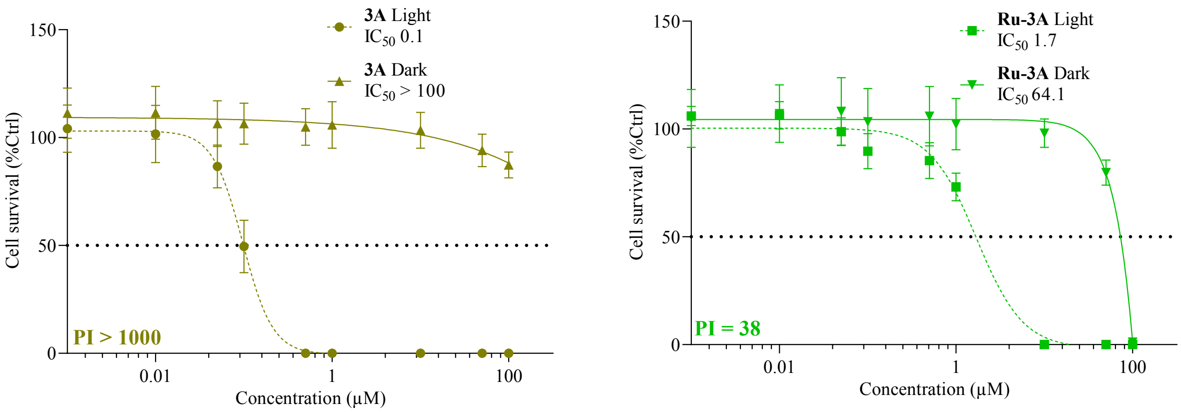

Figure S100. Cell viability curves for the compounds **3B** (left) and **Ru-3B** (right) in the AGS cell line.

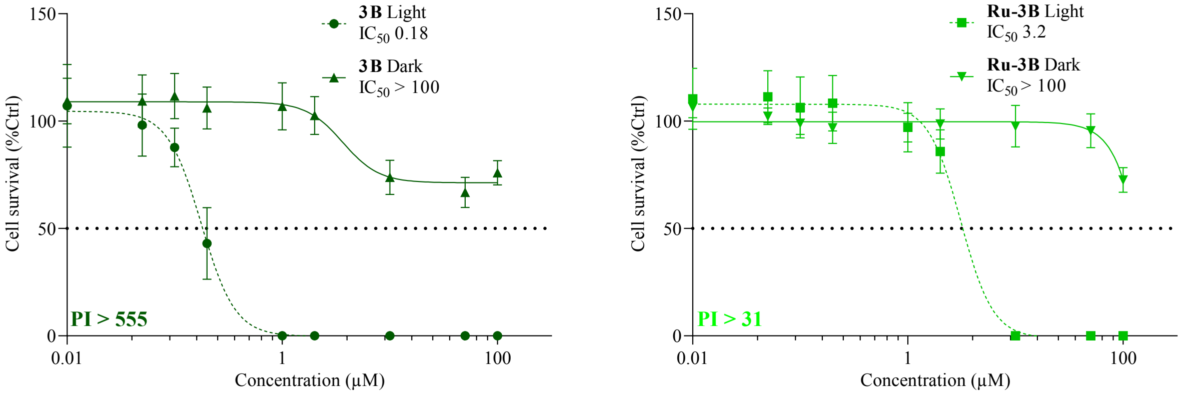

Figure S101. Cell viability curves for the compounds **4A** (left) and **4B** (right) in the AGS cell line.

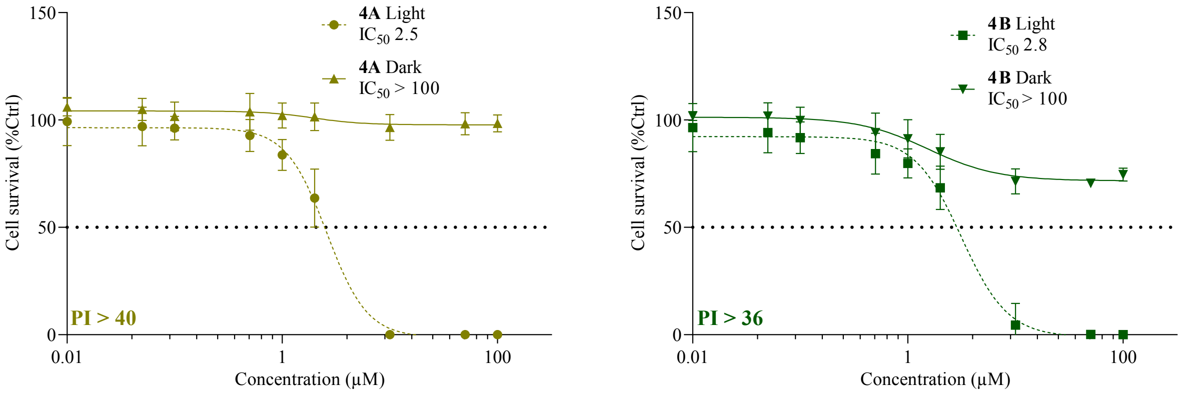

Figure S102. Relative LC3BI/II expression

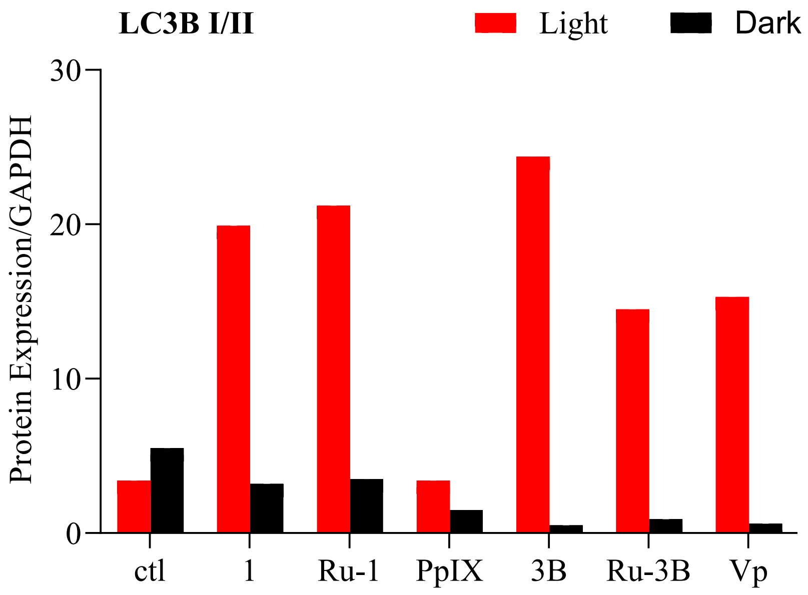

Tables

Table S1. Crystallographic data for **Ru-1** and **3B**

Compound	Ru-1	3B
**Emp. Formula**	$C_{37}H_{38}N_4O_6Ru$	$C_{42}H_{44}N_4O_8$
**FW (g/mol)**	736	732
**Temperature (K)**	298 (2)	101 (2)
**λ (Å)**	0.71073	0.71073
**Crystal system**	Monoclinic	Orthorhombic
**Space Group**	C2/c	*Fdd2*
**a (Å)**	22.896(3)	27.1094(7)
**b (Å)**	13.2913(14)	73.7996(18)
**c (Å)**	26.651(3)	7.4298(2)
**α (Å)**	90	90
**β (Å)**	114.532(4)	90
**γ (Å)**	90	90
**Volumen ($Å^3$)**	7378.3(15)	14864.5(7)
**Z**	8	16
**ρ_{cal} (mg×m^3)**	1.395	1.310
**Abs. Coeff. (mm^{-1})**	0.547	0.746
**F(000)**	3192	6208
**θ range (°)**	2.74 to 30.18	2.39 to 74.35
**Reflections collected / unique**	132312/10693	84611/6876
**[R(int)]**	[0.1318]	[0.0961]
**Completeness (%)**	98.9	99.7
**Data / restraints / parameters**	10693/205/567	6876/103/548
**GoF on F^2**	1.018	1.076
**R1 [I>2σ(I)]**	0.0717	0.1050
**wR2 [I>2σ(I)]**	0.1715	0.1409
**Final R-index**	0.0717	0.1050

Table S2. Spectroscopic properties and $^{1}O_2$ quantum yields in DMSO

**Compound**	**Spectroscopic properties**				
	**λabs/nm (ε/M^{-1} cm^{-1}×10^{-5})**	**λex/nm**	**λem/nm**	**Φ_f/%**	**Φ_Δ/%**
**VP**	358(32359), 436(60256), 578(12023),628(5754),690(25704)	503	693	0.85	77
**1**	406(30199), 506(2691), 540(2138), 576(1318), 630(933)	503	633	0.80	34 ± 0.764
**Ru-1**	404(57544), 524(12023), 558(18621)	573	n.d	0	28 ± 1.000
**2**	408(83176), 506(7413), 540(5888), 576(3631), 630(2570)	503	633	0.80	34 ± 1.528
**Ru-2**	402(57544), 524(8913), 558(12882)	573	n.d	0	30 ± 1.756
**3A**	406(83176), 502(11220), 536(8710), 608(3981), 666(32359)	503	673	1.2	46 ± 2.082
**Ru-3A**	406(67608), 536(6456), 568(8710), 606(17378)	573	n.d	0	15 ± 2.372
**3B**	404(107152), 500(11749), 534(8511),608(4169),666(35481)	503	670	1.2	42 ± 2.500
**Ru-3B**	406(42658), 534(4571), 568(6918), 606(10471)	573	n.d	0	11 ± 2.021
**4A**	406(102329), 502(9332), 536(7413), 608(3981), 666(26303)	503	674	1.0	34 ± 1.041
**4B**	406(97724), 500(12589), 534(8710), 608(3981), 666(38904)	503	670	1.0	33 ± 2.517

λ_{abs} absorption maximum, λ_{em} emission maximum, Φ_f luminescence quantum yield, Φ_Δ $^{1}O_2$ quantum yield at 274 nm and n.d. not detectable.
